# HOX genes promote cell proliferation and are potential therapeutic targets in adrenocortical tumours

**DOI:** 10.1038/s41416-020-01166-z

**Published:** 2020-11-20

**Authors:** Jeffrey C. Francis, Jennifer R. Gardiner, Yoan Renaud, Ritika Chauhan, Yacob Weinstein, Celso Gomez-Sanchez, Anne-Marie Lefrançois-Martinez, Jérôme Bertherat, Pierre Val, Amanda Swain

**Affiliations:** 1 Division of Cancer Biology, The Institute of Cancer Research, 237 Fulham Road, London, UK; 2 Genétique Reproduction & Développement, CNRS UMR 6293, Inserm U1103, Université Clermont Auvergne, 63001 Clermont-Ferrand, France; 3 Tumour Profiling Unit, The Institute of Cancer Research, 237 Fulham Road, London, UK; 4 The Shraga Segal Dept. of Microbiology, Immunology and Genetics, Faculty of Health Sciences, Ben Gurion University of the Negev, Beer Sheva, 84105 Israel; 5 Division of Endocrinology, Medical Service, G.V. (Sonny) Montgomery VA Medical Center, 1500 E. Woodrow Wilson Dr, Jackson, MS 39216 USA; 6 Institut Cochin, Inserm U1016, CNRS UMR 8104, Université Paris Descartes, UMR-S1016, 75014 Paris, France

**Keywords:** Adrenal tumours, Cancer models

## Abstract

**Background:**

Understanding the pathways that drive adrenocortical carcinoma (ACC) is essential to the development of more effective therapies. This study investigates the role of the transcription factor HOXB9 and other HOX factors in ACC and its treatment.

**Methods:**

We used transgenic mouse models to determine the role of *Hoxb9* in adrenal tumour development. Patient transcriptomic data was analysed for the expression of HOX genes and their association with disease. Drug response studies on various adrenocortical models were done to establish novel therapeutic options.

**Results:**

Our human ACC dataset analyses showed high expression of *HOXB9*, and other HOX factors, are associated with poorer prognosis. Transgenic overexpression of *Hoxb9* in the adrenal cortex of mice with activated *Ctnnb1* led to larger adrenal tumours. This phenotype was preferentially observed in male mice and was characterised by more proliferating cells and an increase in the expression of cell cycle genes, including *Ccne1*. Adrenal tumour cells were found to be dependent on HOX function for survival and were sensitive to a specific peptide inhibitor.

**Conclusions:**

These studies show *Hoxb9* can promote adrenal tumour progression in a sex-dependent manner and have identified HOX factors as potential drug targets, leading to novel therapeutic approaches in ACC.

## Background

Tumours of the adrenal cortex are relatively common with a prevalence of 1–10%. Most of these are benign adenomas, however, in rare cases (up to 2 per million per year) adrenocortical carcinoma (ACC) can develop, which is an aggressive disease with a low 5-year survival (up to 35% of diagnosed patients and less than 15% in patients with metastatic disease) (reviewed in refs. ^1,2^). Current treatments include surgery for non-metastatic disease and mitotane therapy, which has shown limited efficacy in advanced patients. Understanding the pathways that drive ACC progression is essential to the development of more effective treatments and for the prediction of individual outcomes.

Relatively little is known about the molecular pathways that drive ACC growth and progression. Recent next-generation sequencing studies have revealed recurrent alterations present in patients with ACC.^3–5^ These studies have identified mutations in the WNT signalling pathway to be one of the most frequent alterations, with *CTNNB1-*activating mutations present in up to 16% and inactivating changes in *ZNRF3*, a pathway repressor, in up to 21%.^3^ Other pathways that are altered in ACC patients include epigenetic regulation and p53/RB1 and PKA signalling.^5^

Studies in mice have shown that targeting an activating mutation in *Ctnnb1* to the adrenal cortex leads to tumour formation, highlighting the relevance of this model to study this disease.^6^ WNT signalling has been implicated in adrenal development and homoeostasis. Genetic studies in mice have shown that a decrease in WNT signalling, either through deletion of *Ctnnb1* or pathway inhibition, in adrenal cortical cells led to adrenal aplasia, degeneration and zonation defects, depending on the deletion stage and cell type.^7–9^ These studies also indicate the role of WNT signalling in subcapsular stem/progenitor cell proliferation and maintenance.^10^

Factors involved in organ development have been implicated in neoplasia in many tissues. HOX genes are a family of homeobox transcription factors that are major regulators of embryogenesis and have been implicated in oncogenesis.^11^ In mammals there are four clusters of HOX genes (*HOXA*, *HOXB*, *HOXC* and *HOXD*) with 39 members identified in humans. Gene expression studies have shown that several HOX genes are expressed in the developing and adult adrenal glands, while the HOX cofactor PBX1 is required for adrenal formation.^12–15^ Emerging evidence has revealed that aberrant HOX gene expression in adult tissues can lead to malignancy, including in lung, ovarian, cervical, prostate and breast carcinoma,^16–21^ and can interact with other signalling pathways, such as WNT/β-catenin, to promote tumour progression.^22–26^

Here, we show that high expression of *HOXB9*, which is expressed in adrenal cortical cells during development, is associated with patient survival in ACC. A genetic mouse model of overexpression of *Hoxb9* in adrenal cortical cells shows a bigger X zone. Male mice with an activating *Ctnnb1* mutation and *Hoxb9* overexpression develop tumours with increased proliferation. Our study implicates HOX genes in ACC formation and identifies them as possible drug targets in this disease.

## Methods

### Mouse strains

The mutant *Ctnnb1* mice were generated by breeding two previously described strains, Cyp11a1:Cre and the stabilised *Ctnnb1* conditional allele.^27,28^ The Sf-1:Hoxb9 transgenic mice were generated by injecting a BAC construct into one-cell mouse embryos, as described previously.^27^ The BAC construct was generated by inserting, through homologous recombination, the mouse *Hoxb9* cDNA plus the bovine growth hormone polyadenylation signal sequences into the SacII site in the 5′ untranslated region of the mouse *Sf-1* locus contained in a BAC plasmid, as described previously.^27^

All animals were bred on a mixed genetic background. All mouse work was carried out in accordance with the Institute of Cancer Research guidelines and with the UK Animals (Scientific Procedures) Act 1986. Animals were housed in a specific pathogen-free environment using an Optimouse system (air speed 4.3 m/s average, a 12/12 light cycle (7:30–19:30), temperature 21 + /− 1 °C, room humidity 55% +/− 10%), and fed lab diet 5002 (International Product Supplies), with corn-cob bedding 1014 (International Product Supplies), and with card tunnel and woodblock enrichment. Animals were euthanised by exposure to carbon dioxide or isoflurane followed by cervical dislocation. At least four animals of each sex were analysed for each genotype, using animals from the same litters as controls.

### Immunohistochemistry (IHC)

Antibody staining was performed as previously described (details in the Supplementary Methods section). For adrenal sections, staining was done on samples from at least three mice of the same genotype.

### Western blot

Cells were lysed in RIPA buffer (Sigma, Welwyn Garden Cit, UK) with protease and phosphatase inhibitors (Cell Signaling Technology, Leiden, The Netherlands). Samples were run on 4–12% Bis-Tris protein gels with MOPS buffer and transferred to nitrocellulose membranes. The membrane was blocked in 5% milk/TBST (TBS, 0.1% Tween-20) for 1 h, and primary antibodies incubated in 2.5% milk/TBST overnight at 4 °C. Membranes were washed with TBST, incubated with HRP-conjugated secondary antibodies for 1 h at room temperature, washed again and chemiluminescence detected (GE Healthcare, Chalfont St Giles, UK). Primary antibodies used were; HOXB9 (Santa Cruz sc-398500), Sf-1 (Abcam ab65815) and Vinculin (Sigma V4505).

### Quantification of cell proliferation and cell death

The number of proliferating cells was calculated by counting the number of nuclear Ki67-stained cells and shown as a percentage of the total number of cells stained with nuclear haematoxylin. Apoptotic cell death of mouse tissue was quantified by IHC stains of sections with an antibody against active Caspase 3. Cells were counted from at least four high-power randomly selected fields per animal. Three animals of each genotype were analysed. An ANOVA or *t* test was used to test if there was a significant difference in the number of proliferating or apoptotic cells between each group.

### In vitro culture of adrenal cells

H295R cells, a kind gift from Peter King (Queen Mary, University of London), were STR profiled to confirm their identity and were maintained in DMEM/F12 supplemented with 2.5% Nu-Serum I, ITS + 1 and GlutaMAX. ATC1 and ATC7 cells were established from adrenal tumours from transgenic mice with Large T antigen of SV40 controlled by the cortical adrenal-specific *Akr1b7* promoter, and were a kind gift from Antoine Martinez.^29^ ATC1 and ATC7 cells were maintained in DMEM/F12 supplemented with 2.5% FBS, 2.5% horse serum and ITS. PC3 cells were maintained in RPMI1640 with 10% FBS. All cell lines were used at low passage number (<20). ABC cells were derived from a mouse *Ctnnb1* mutant adrenal gland tumour and grown in Y-media (DMEM/F12, 10% FBS, penicillin/streptomycin, L-Glutamine, 5 μg/ml Insulin, 0.4 μg/ml hydrocortisone, 10 ng/ml EGF, 10 μg/ml Gentamicin, 250 ng/ml amphotericin, 4.81 ng/ml Cholera toxin, 5 μM Y27632). Briefly, adrenal glands were dissected, minced and incubated in dissociation media (DMEM/F12, ITS, 10 μg/ml EGF, 10 mg/ml hydrocortisone, 0.5 mg/ml collagenase, 0.1 mg/ml hyaluronidase, 100 units/ml DNase I, 10 μM Y27632) for 2 h. The cells were washed, incubated in 0.05% Trypsin/EDTA with 10 μM Y27632 for 8 min at 37 °C and dissociated by vigorous pipetting and trypsin inactivated with Y-media. Cells were incubated in 1 mg/ml DNase for 5 min at 37 °C, washed with PBS and filtered through 70-μm filter.

### siRNA knockdown in H295R cells

To perform gene knockdown, 6 × 10^5^ H295R cells were seeded in six-well plates and the next day siRNA reagents non-targeting ON-TARGETplus SMARTpool (D-001810-10-05, Horizon Discovery, Cambridge, UK), PBX1 ON-TARGETplus SMARTpool (L-019680-00-0005), HOXA10 ON-TARGETplus SMARTpool (L-006336-00-0005), HOXA11 ON-TARGETplus SMARTpool (L-012108-00-0005), or HOXA13 ON-TARGETplus SMARTpool (L-011052-00-0005) were transfected with RNAiMAX (Thermo Fisher Scientific, Hemel Hempstead, UK) following the manufacturer’s protocol. Cells were incubated for a further 24 h and re-plated for the cell growth assay or 48 h for RNA extraction. For the growth assay, 2000 cells were plated per well of a 96-well plate and CellTitre-Glo luminescence (Promega, Southampton, UK) measurements were taken at the indicated days.

### Lentiviral overexpression of HOXB9

H295R cells overexpressing HOXB9 were made by transduction with lentiviral particles generated from pLenti-GIII-CMV-HOXB9-GFP-2APuro (LV183492, Applied Biological Materials, Richmond, Canada) and control cells with pLenti-CMV-GFP-2A-Puro (LV590, Applied Biological Materials). Lentivirus was produced by transfecting 7 × 10^6^ 293T cells in 10-cm plates with the packaging plasmids psPAX2 and pMD2.G using Lipofectamine 3000 (Thermo Fisher Scientific). Viral supernatants were collected 24- and 48-h post-transfection, centrifuged at 2000 rpm, and filtered through a 45-μm filter.

### Generation of a HOX-targeting peptide

Genscript Biotech (Leiden, The Netherlands) synthesised a peptide targeting the HOX–PBX protein interaction and a control peptide, using sequences based on previous publications.^30^ The targeting HTL001 peptide sequence WYKWMKKAARRRRRRRRR and the CXR9 control peptide WYPAMKKHHRRRRRRRRR, both with D-isomer modification of C- and N-terminals were dissolved in DMSO.

### Cell viability assay

Peptide drug survival assays were performed in 96-well plates. PC3 cells were plated at 500 cells per well; H295R, ATC1, ATC7 and ABC cells were plated at 2000 cells per well. Drug was added 24 h after seeding, and cells were continuously exposed to the drug for 5 days, after which cell viability was estimated using CellTitre-Glo luminescence (Promega).

### Apoptosis assay

H295R cells were plated in a 96-well plate at 10,000 cells per well. The next day the IC_50_ concentration of 5 μM HTL001, 5 μM CXR9 or DMSO was added, incubated for 24 h and apoptosis estimated using the Caspase 3/7 Glo assay (Promega) following the manufacturer’s protocol.

### RNA isolation and quantitative RT-PCR

RNA was isolated using the RNeasy kit (Qiagen, Manchester, UK) and cDNA was made using 500 ng RNA with SuperScript IV reverse transcriptase (Thermo Fisher Scientific), following the manufacturer’s instructions. Quantitative RT-PCR was carried out using Taqman gene expression assays (probe sequence in the Supplementary Methods section). Experiments on animal tissue were done from at least 3 mice of the same genotype.

### Transcriptomic analysis

For RNA-seq analysis of adrenal tumours, mRNA libraries for were prepared using 500 ng of total RNA of each sample with NEBNext Ultra II Directional RNA Library Prep kit (E7760, NEB, Hitchin, UK) and NEBNext Poly(A) mRNA magnetic beads (E7490, NEB) following the manufacturer’s protocol. RNA samples were run on two lanes of a HiSeq2500 using RAPID onboard clustering and single read 50 cycles. All samples were run at 16.6% per lane to achieve 25 million clusters. TopHat RNA-seq alignment software (v2.1.0) was used to align reads to the reference mouse genome (GRCm38). Once the reads were aligned, HTSeq-count (HTSeq v0.6.1) was used to count the number of reads mapping unambiguously to genomic features in each sample. The raw count data were normalised to correct for library size and RNA composition bias. Differential gene expression analysis of the count data was performed in R using the Bioconductor package DESeq2 (v1.18.1). Gene set enrichment analysis (GSEA) was carried out with male *Ctnnb1* mutant and double-mutant RNA-seq data using the Broad Institute Java plug-in with the Hallmarks gene set.^31^ STRING protein network analysis was carried out using the differentially expressed genes between male *Ctnnb1* mutants and double mutants (genes with adjusted *P* < 0.1 and *P* < 0.05).

### Patients cohort datasets

The clinical datasets used in this paper are derived from two cohorts of patients. Cochin’s cohort (49 ACC, 101 ACA and 4 NAd, GSE49280) was analysed by Affymetrix microarray (HG-U133 Plus 2.0).^3^ Our collaborators provided data analysed after RMA normalisation and inter-array normalisation. The other transcriptome (79 patients) is from the TCGA database (The Cancer Genome Atlas) and it relies on Illumina HiSeq 2000 RNA Sequencing Version 2.^5^ Expression data were standardised by the Relative Standard Error of the Mean (RSEM) algorithm and transformed into Log2 in order to refocus and symmetrise values’ distribution. For each cohort, patients’ survival data were available and distribution in the good (C1B) and poor prognosis (C1A) groups, which was defined by our collaborators on the basis of unsupervised clustering. Gene proliferation signature was extracted from Wassef et al.^32^ (Supplementary Table S1) and was defined as the mean expression of the 61 genes that compose it. Statistical analyses were performed in R using the lmerTest_3.1-0 and lsmeans_2.30-0 libraries. Paired comparisons (C1A vs C1B) were analysed by a Wilcoxon test. Multiple comparisons (NAd, ACA and ACC) were analysed by ANOVA followed by Tukey’s test. Overall and disease-free survival analyses were conducted with R package « survival_2.44-1.1 » and displayed as Kaplan–Meier Curves. Pearson correlations were computed using R package ≪ Hmisc_4.2-0 ≫. Graphs were generated using R library ≪ ggplot2_3.1.1 ≫.

## Results

### Hoxb9 overexpression in adrenal cortical cells

**Fig. 1:**
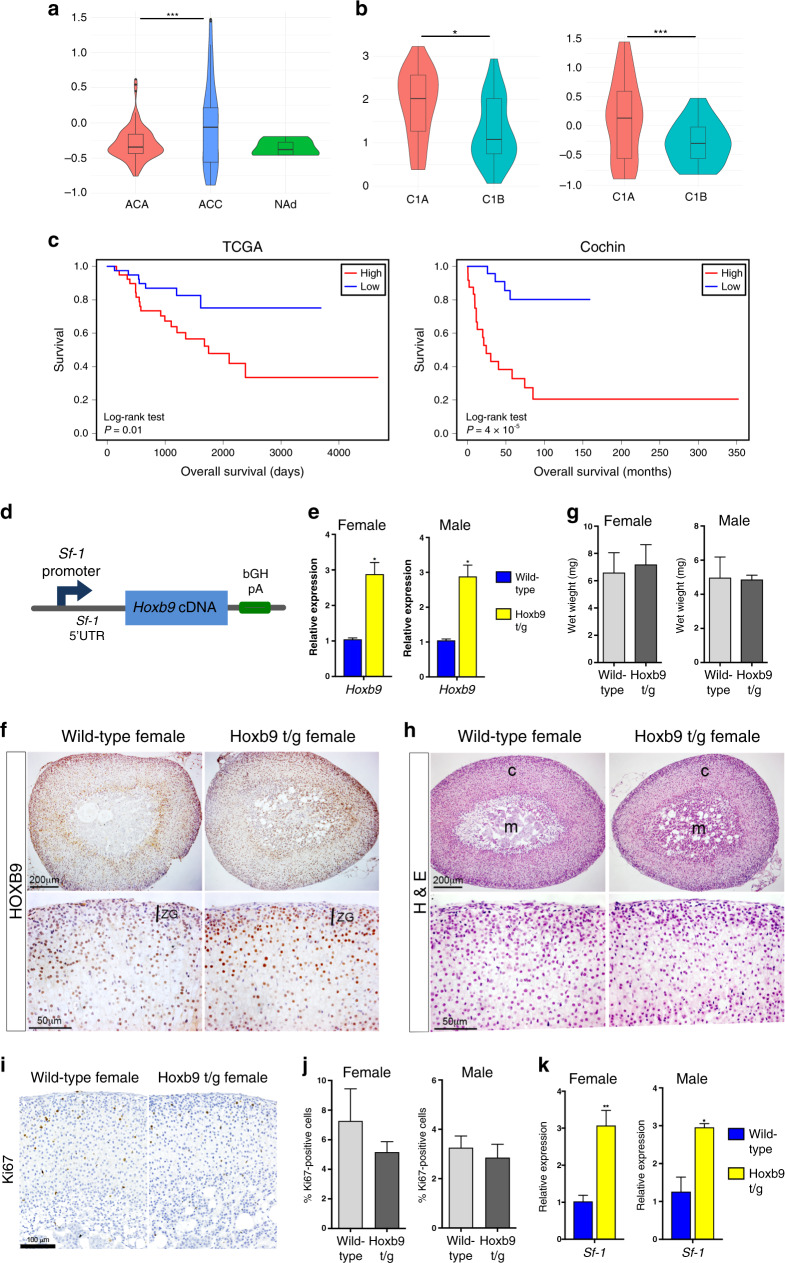
*HOXB9* is associated with aggressive ACC and the generation of a transgenic mouse overexpressing *Hoxb9* in adrenal cells. **a** *HOXB9* gene expression in normal human adrenals (NAd), adrenocortical adenoma (ACA) samples, and adrenocortical carcinoma (ACC) samples from the Cochin cohort. Statistical analysis is one-way ANOVA Turkey’s pairwise test, ***P = 0.0004. **b** *HOXB9* expression in ACC samples from patients with aggressive C1A or indolent C1B-type disease from TCGA and Cochin cohorts. Statistical analysis is a Wilcoxon test, ***P = 0.00053, *P = 0.04. **c** Kaplan–Meier survival curves for ACC patients from the TCGA and Cochin cohorts that had either high or low *HOXB9* expression. **d** Schematic of the Sf-1:Hoxb9 transgenic construct used to increase *Hoxb9* expression in adrenal glands. bGH pA is a bovine growth hormone polyA sequence. **e** qRT-PCR of *Hoxb9* on wild-type and Hoxb9 t/g adrenal glands. The data represent mean ± SD from three biological repeats. **f** HOXB9 immunohistochemistry on sections of female wild-type and Hoxb9 t/g adrenal glands. ZG is zona glomerulosa. **g** Wet weights of male and female adrenal glands from wild-type and Hoxb9 t/g animals. The data represent mean ± SD from four samples. **h** Haematoxylin and eosin (H&E) stain on sections from wild-type and Hoxb9 t/g adrenal glands. c is the cortex, m is the medulla. **i** Ki67 immunohistochemistry on sections of wild-type and Hoxb9 t/g female adrenal glands. **j** Bar chart of the percentage of Ki67-positive cells in wild-type and Hoxb9 t/g male and female adrenal glands. The data represent the mean ± SD from three biological repeats. **k** qRT-PCR of *Sf-1* on wild-type and Hoxb9 t/g adrenal glands. The data represent the mean ± SD from three biological repeats. Student’s *t* test, **P < 0.01, *P < 0.05. Hoxb9 t/g indicates Sf-1:Hoxb9 transgenic.

HOX genes have been implicated in the initiation and progression of many cancers.^11^ Our and other studies have shown that *Hoxb9* is expressed at the early stages of adrenal cortical development.^14,33^ To investigate if *HOXB9* expression is associated with ACC, we analysed patient gene expression data from the Cochin cohort that contains normal adrenal (NAd), adrenocortical adenoma (ACA) and ACC samples.^3^ In this dataset, *HOXB9* expression was higher in ACC samples with the difference with ACA being significant, but not with NAd (Fig. 1a). Consensus clustering of mRNA expression has been used to subgroup ACC patients into those that have aggressive disease, C1A, and those with the indolent disease, C1B. In both the Cochin dataset and in ACC samples from The Cancer Gene Atlas (TCGA), *HOXB9* expression was significantly higher in C1A compared to C1B (Fig. 1b). Consistent with *HOXB9* expression being associated with aggressive disease, analyses of TCGA and Cochin patients into high and low *HOXB9* expression showed that ACC patients with high *HOXB9* expression had a poorer survival prognosis (Fig. 1c). Together these data suggest that elevated *HOXB9* expression in ACC may play a role in tumour progression to aggressive disease.

**Fig. 2:**
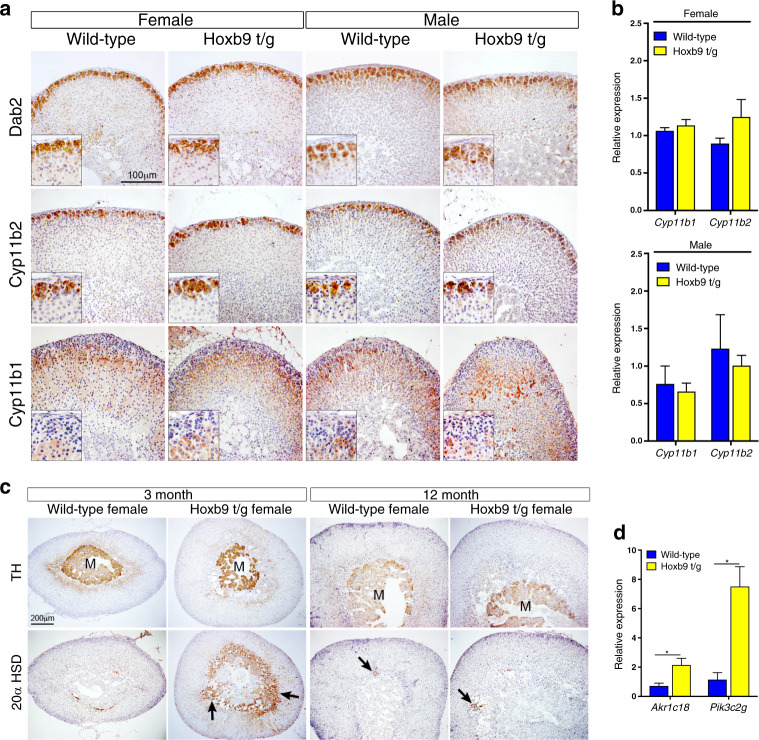
*Hoxb9* transgenic animals have a larger X zone. **a** Dab2, Cyp11b2 and Cyp11b1 immunohistochemistry on sections from wild-type and Hoxb9 t/g male and female adrenal glands. **b** qRT-PCR of *Cyp11b1* and *Cyp11b2* on wild-type and Hoxb9 t/g adrenal glands. The data represent the mean ± SD from three biological repeats. **c** Tyrosine Hydroxylase (TH) and 20α-HSD immunohistochemistry on sections from wild-type and Hoxb9 t/g 3- and 12-month-old female adrenal glands. **d** qRT-PCR of *Akr1c18* and *Pik3c2g* on female wild-type and Hoxb9 t/g adrenal glands. The data represent the mean ± SD from three biological repeats. m is the medulla, arrows indicate positive cells. Student’s *t* test, *P < 0.05. Hoxb9 t/g indicates Sf-1:Hoxb9 transgenics.

To investigate the effect of high levels of *HOXB9* in adrenal cortical cells, we generated transgenic mice carrying a BAC construct that contained the *Hoxb9* cDNA inserted into the *Sf-1* locus (Sf-1:Hoxb9 mice, referred to as Hoxb9 t/g, Fig. 1d). Quantitative RT-PCR (qRT-PCR) expression analysis showed an increase of *Hoxb9* levels in the adult adrenals of transgenic mice (Fig. 1e). This was confirmed in antibody staining studies, which showed an expanded domain of HOXB9 expression, reflecting the pattern of the *Sf-1* promoter sequence driving *Hoxb9* (Fig. 1f and Supplementary Fig. S1A). In the normal adrenal HOXB9 was primarily expressed in zona fasciculata (ZF), while in transgenic animals expression was also found in the outer cortical zona glomerulosa (ZG) cells. HOXB9-expressing cells were also found in the medulla of transgenic glands from 3-month-old female mice that were not present in control animals (Supplementary Fig. S1B). Phenotypic analysis of the adrenals of transgenic mice at 3 and 18 months of age showed no obvious changes in size or structure compared to wild-type adrenal glands (Fig. 1g, h, Supplementary Fig. S1C, D). Ki67 staining revealed no difference in the number of proliferating cells between transgenic and wild-type animals (Fig. 1i, j and Supplementary Fig. S1E). We next analysed the expression of a known embryonic HOX target gene, *Sf-1*, markers of adrenal gland stem/progenitor cell function, and a WNT signalling target. In both female and male adrenal glands from transgenic mice, *Sf-1* expression was increased threefold, while there was no change in the expression of *Shh*, *Patched* (*Ptch*) or *Axin2* (Fig. 1k and Supplementary Fig. S1F). This suggests that elevated *Hoxb9* is not able to induce neoplastic development but can promote *Sf-1* expression in the adult gland. To determine if adrenal gland zonation was disrupted by elevated *Hoxb9* expression, we performed immunohistochemical (IHC) staining and qRT-PCR analyses with cell-type markers for ZG (Dab2 and Cyp11b2), ZF (Cyp11b1), adrenal medulla (TH) and X zone (20α-HSD, gene name *Akr1c18*, and *Pik3c2g*) on male and female adrenals (Fig. 2). These data showed that transgenic animals had no change in adrenal cortical ZG or ZF markers (Fig. 2a, b). Instead, glands from 3-month-old female transgenic mice had a bigger foetal derived X zone with Sf-1-positive cells infiltrating the medulla (Fig. 2c, d and S2A). The larger X zone in transgenic animals behaved as in the wild-type in that it was only found in prepubertal males (Supplementary Fig. S2B) and it regressed in older females (Fig. 2c).

### Elevated *Hoxb9* cooperates with mutant *Ctnnb1* during tumour formation

**Fig. 3:**
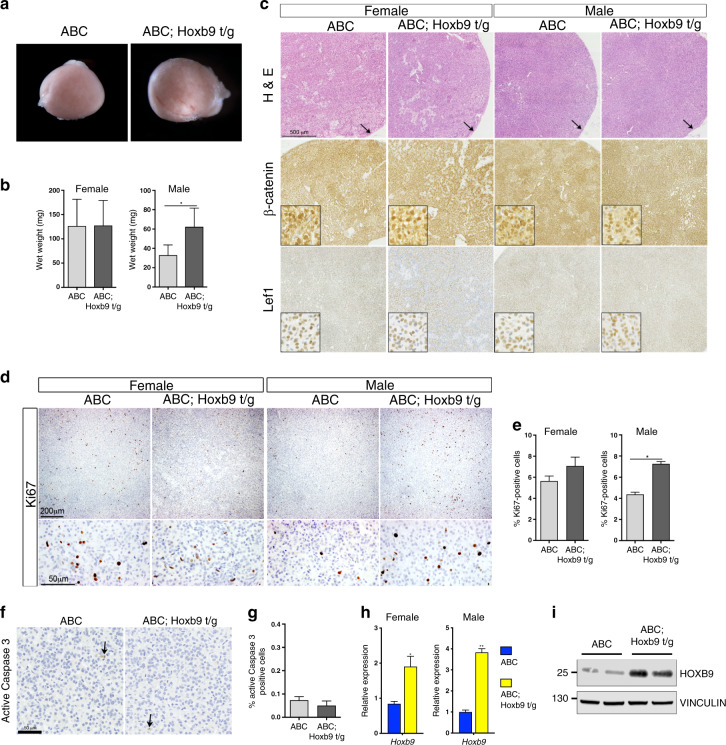
*Hoxb9* cooperates with *Ctnnb1* during tumour formation. **a** Bright-field images of male ABC and ABC; Hoxb9 t/g adrenal glands. **b** Wet weight of 6-month-old female and male ABC and ABC; Hoxb9 t/g adrenal glands. The data represent the mean ± SD from four tumours. **c** H&E, β−catenin and Lef1 immunohistochemistry on sections from ABC and ABC; Hoxb9 t/g adrenal tumours. Inset shows high-power magnification. Arrow indicates capsule. **d** Ki67 immunohistochemistry on sections from 6-month-old ABC and ABC; Hoxb9 t/g adrenal tumours. **e** Bar chart of the percentage of Ki67-positive cells in ABC and ABC; Hoxb9 t/g adrenals. The data represent the mean ± SD from three biological repeats. **f** Active Caspase 3 immunohistochemistry on sections from ABC and ABC; Hoxb9 t/g male tumours. Arrows indicate positive cells. **g** Bar chart of the percentage of Caspase 3-positive cells in ABC and ABC; Hoxb9 t/g male adrenals. The data represent the mean ± SD from three biological repeats. **h** qRT-PCR of *Hoxb9* on ABC and ABC; Hoxb9 t/g adrenal tumours. The data represent mean ± SD from three biological repeats. **i** Western blot analysis of Hoxb9 on ABC and ABC; Hoxb9 t/g adrenal tumours from female animals. Adrenals from two animals of each genotype are shown. Vinculin is used as a loading control. ABC indicates *Ctnnb1* mutant tumours, ABC; Hoxb9 t/g indicates double-mutant tumours. Student’s *t* test, **P < 0.01, *P < 0.05.

To investigate if HOXB9 can promote tumour formation we bred Sf-1:Hoxb9 mice with mice containing the activating conditional *Ctnnb1* deletion allele and a construct with Cre recombinase driven by *Cyp11a1*-regulatory sequences (*Ctnnb1* mutant mice, referred to as ABC). As expected, adrenals from *Ctnnb1* mutant mice showed tumour formation characterised by increased organ size, which was larger in the female (Figs. 1g and 3b). These tumours had a lack of zonal structure, loss of medullar cells and high expression of the ZG marker Dab2 throughout the tumour (Supplementary Fig. S3A, B). Six-month-old *Ctnnb1* mutant mice that also carried the Sf-1:Hoxb9 transgene (double-mutant mice, referred to as ABC; Hoxb9 t/g) showed an increase in adrenal size, which was restricted to male mice (Fig. 3a, b). Haematoxylin and eosin staining showed no obvious morphological difference between *Ctnnb1* and double mutants (Fig. 3c). As expected, antibody staining for β-catenin and the WNT signalling downstream marker Lef1 in *Ctnnb1* mutants showed high expression in the majority of cells (Fig. 3c). This staining pattern was unchanged in double-mutant tumours showing that elevated *Hoxb9* had no major effect on this pathway (Fig. 3c). Proliferation, as measured by Ki67 staining, was higher in 6-month-old double-mutant male mice, with no changes in apoptosis, as measured by Caspase 3 staining (Fig. 3d–g). No signs of invasive disease were observed in double-mutant animals (Fig. 3c, arrows). Adrenal tumours from double-mutant mice expressed higher levels of *Hoxb9* mRNA and protein than single *Ctnnb1* mutants, confirming expression of the transgene (Fig. 3h, i). As Sf-1:Hoxb9 adrenals showed an increase in *Sf-1* expression, we investigated the levels of this gene in the tumours. qRT-PCR analysis showed that *Sf-1* transcript was higher in both female and male double-mutant adrenal tumours relative to *Ctnnb1* mutants, but there was no difference in the levels of SF-1 protein expression between the genotypes (Supplementary Fig. S3C, D).

**Fig. 4:**
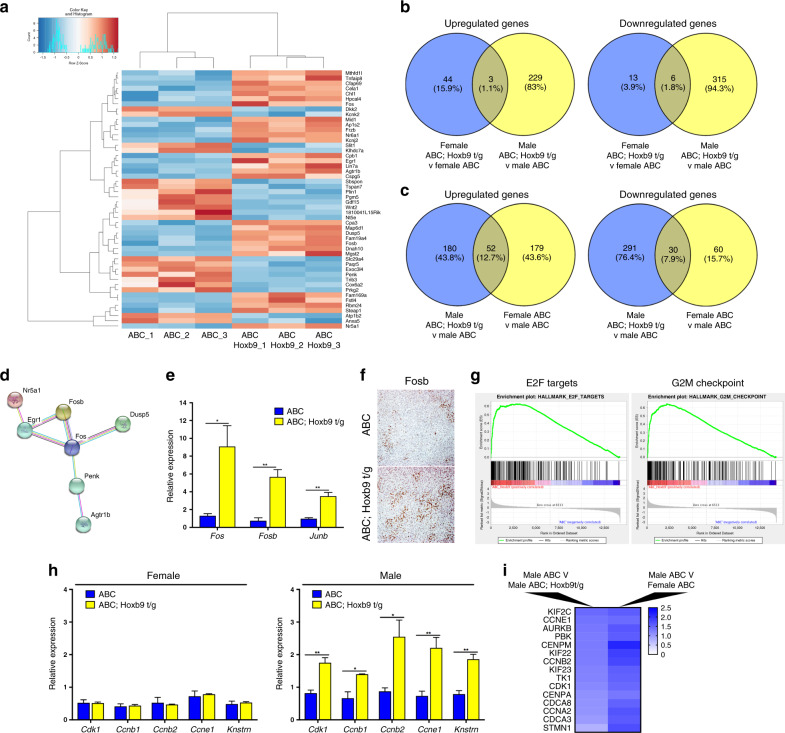
Elevated HOX expression leads to an increase in cell cycle gene expression. **a** Heatmap of the top 50 differentially expressed genes identified from RNA-seq data of male ABC; Hoxb9 t/g adrenal tumours compared to male ABC adrenal tumours. **b** Comparison of differentially expressed genes in male and female ABC; Hoxb9 t/g adrenal tumours compared to ABC adrenal tumours. **c** Comparison of differentially expressed genes in male ABC; Hoxb9 t/g tumours compared in male ABC tumours to the genes differentially expressed genes in female ABC tumours compared to male ABC tumours. **d** STRING database network of differentially expressed genes in male ABC; Hoxb9 t/g tumours compared to male ABC tumours identifies angiotensin signalling. **e** qRT-PCR of *Fos*, *Fosb* and *Junb* in male ABC and ABC; Hoxb9 t/g tumours. The data represent mean ± SD from three biological repeats. **f** IHC of Fosb in 3-month-old male ABC; Hoxb9 t/g and ABC adrenal tumours. **g** GSEA of RNA-seq data from male ABC; Hoxb9 t/g and male ABC adrenal tumours. E2F targets normalised enrichment score (NES) = 1.57, FDR *q* = 0.069, G2M checkpoint NES = 1.70, FDR *q* = 0.043. **h** qRT-PCR of *Cdk1*, *Ccnb1*, *Ccnb2*, *Ccne1* and *Knstrn* in female and male ABC and ABC; Hoxb9 t/g adrenal tumours. The data represent mean ± SD from three biological repeats. **i** Cell cycle genes upregulated in male ABC; Hoxb9 t/g tumours and female ABC tumours (compared to ABC males). Student’s *t* test, **P < 0.01, *P < 0.05. ABC indicates *Ctnnb1* mutant tumours, ABC; Hoxb9 t/g indicates double-mutant tumours.

To investigate the pathways activated in the double mutants we performed RNA-seq on RNA derived from adrenal tumours from 3-month-old *Ctnnb1* and double-mutant female and male mutant mice. Comparative analysis identified differentially expressed genes in the tumours of double-mutant mice compared to *Ctnnb1* mutants, with a higher number in males (533 genes altered, Benjamini–Hochberg adjusted *P* < 0.1) than in the same comparison in females (66 genes altered, Benjamini–Hochberg adjusted *P* < 0.1) (Fig. 4a and Supplementary Tables S2–S5). For both sexes, genes were differentially up- and downregulated (males 232 genes up, 322 genes down; females 47 up, 19 genes down), consistent with evidence suggesting that HOX proteins can act as transcriptional activators and repressors.

Comparative analysis of the differentially expressed genes between double mutants and *Ctnnb1* mutant female and male tumours showed very few common genes altered in both sexes (three genes upregulated, and six genes downregulated in both females and males) (Fig. 4b). Validating our qRT-PCR result, *Sf-1* was increased in double mutants of both sexes. Interestingly, many genes that were differential between male double mutants and *Ctnnb1* mutants were shared with those that were different between male and female *Ctnnb1* mutant animals (52 upregulated genes and 30 downregulated genes) (Fig. 4c). These data suggest that *Hoxb9* acts to promote tumorigenic pathways that are repressed in the male adrenal.

Pathway analysis of the differentially expressed genes using the STRING protein–protein interaction database identified angiotensin signalling enriched in double-mutant male tumours compared to *Ctnnb1* mutant tumours, including *Agtr1b, Egr1, Nr4a1* and members of the Fos/Jun family *Fos, Fosb and Junb* (Fig. 4d and Supplementary Table S4).^34^ Interestingly, *Cyp11b2* expression, a target of this pathway, was not changed in these tumours. qRT-PCR was used to validate these results for the Fos/Jun family, and antibody staining showed widespread expression of Fosb in double-mutant tumours (Fig. 4e, f). Gene set enrichment analysis of the RNA-seq data from male *Ctnnb1* and double-mutant tumours revealed enrichment in cell cycle genes in tumours with elevated *Hoxb9*, consistent with the increase in proliferation in double mutants (Fig. 4g and Supplementary Table S6). These data were validated using qRT-PCR for *Cdk1*, *Ccnb1*, *Ccnb2*, *Ccne1* and *Knstrn*, which showed an increase in these genes in male double mutants but not in females (Fig. 4h). We next checked if the pathways we identified were also altered in female *Ctnnb1* mutant tumours, compared to males of the same genotype, as these tumours shared a large number of altered genes with male double mutants (Fig. 4c). We found 15 cell cycle regulatory genes (E2F targets or G2M checkpoint Hallmark genes) upregulated in female *Ctnnb1* tumours compared to *Ctnnb1* male tumours, including *Ccne1* and *Cdk1* (Fig. 4i). These data suggest a core set of cell cycle genes that are elevated in tumours with high *Hoxb9* are also expressed at high levels in female *Ctnnb1* tumours, compared to males.

### HOX factors as potential drug targets in ACC

**Fig. 5:**
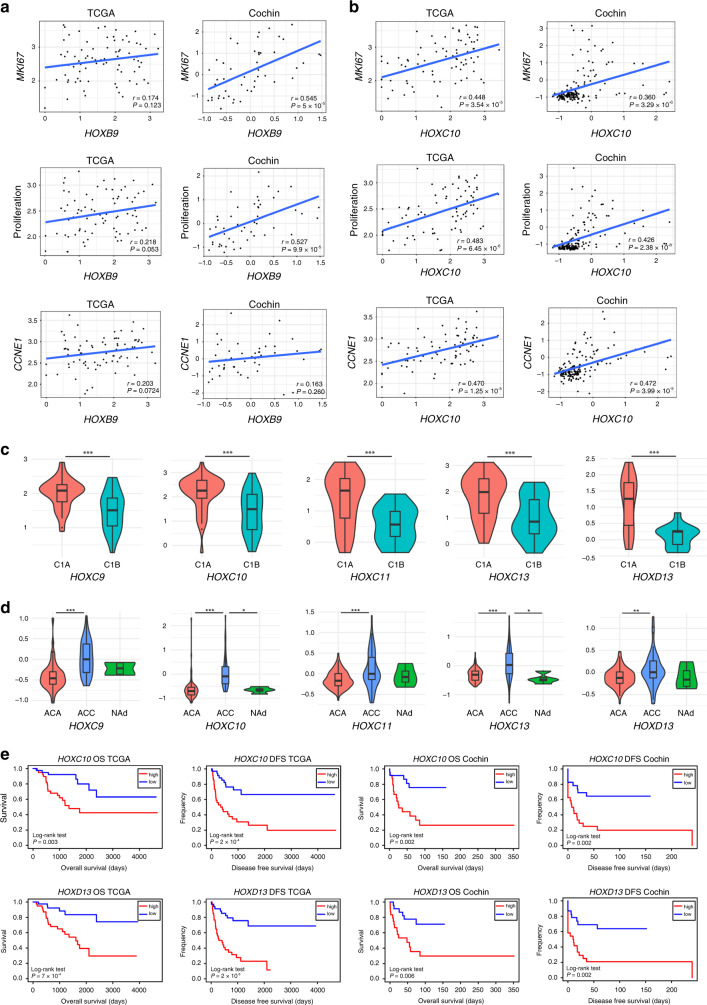
HOX gene expression is associated with aggressive ACC. **a** Correlation of *HOXB9* expression with *MKI67*, a proliferation gene signature, and *CCNE1* in ACC patient samples from the TCGA and Cochin cohorts. **b** Correlation of *HOXC10* expression with *MKI67*, a proliferation gene signature, and *CCNE1* in ACC patient samples from the TCGA and Cochin cohorts. **c** *HOX* gene expression in ACC samples from patients with aggressive C1A or indolent C1B-type disease from the TCGA cohort. Statistical analysis a Wilcoxon test, ***P < 0.001. **d** *HOX* gene expression in normal human adrenals (NAd), adrenocortical adenoma (ACA) samples, and adrenocortical carcinoma (ACC) samples from the Cochin cohort. Statistical analysis is one-way ANOVA Turkey’s pairwise test, ***P < 0.001, **P < 0.01, *P < 0.05. **e** Kaplan–Meier survival curves for ACC patients from the TCGA and Cochin cohorts that had either high or low *HOXC10* or *HOXD13* expression.

Our transgenic mouse data indicates that high *Hoxb9* expression can promote cell proliferation within a tumour context. We next wanted to determine if *HOXB9* can promote proliferation in human ACC cells. Consistent with our mouse studies, overexpression of *HOXB9* in the human adrenal cortical tumour cell line H295R led to a small but significant increase in cell number (Supplementary Fig. S4A, B, C). To determine if *HOXB9* expression correlates with proliferation in human samples we analysed its expression in two ACC datasets, TCGA and Cochin, with the proliferation markers *MKI67*, *CCNE1* and an established proliferation gene signature (Wassef et al.^32^) (Fig. 5a). We found a significant correlation of *HOXB9* with *MKI67* and the proliferation signature in the Cochin ACC dataset but not TCGA. To investigate if other HOX genes are implicated in proliferation in human ACC we performed these correlations with all members of the HOX gene family. Several HOX genes showed a significant correlation with all proliferation markers in both datasets, including *HOXC9, HOXC10, HOXC11, HOXC13* and *HOXD13* (*MKI67* *P* < 0.002, proliferation gene signature *P* < 0.001, CCND1 *P* < 0.05) (Fig. 5b, Supplementary Fig. S5, Supplementary Tables S7 and S8). An analysis of the correlation of HOX gene expression with the expression of all other HOX members in the TCGA ACC dataset showed *HOXB9* expression significantly correlated with the expression of these HOX genes (*HOXC9* *r* = 0.377; *P* = 0.000604, *HOXC10* *r* = 0.474; *P* = 9.81^−06^*, HOXC11* *r* = 0.427; *P* = 8.60^−05^*, HOXC13* *r* = 0.455; *P* = 1.16^−06^ and *HOXD13* *r* = 0.453; *P* = 2.69^−05^) (Supplementary Tables S7, S8 and Supplementary Fig. S6).

To establish if these genes were implicated in disease progression, we performed correlations between HOX gene expression and ACC C1A versus C1B status (Fig. 5c and Supplementary Fig. S7), and ACC versus ACA and NAd (Fig. 5d), and found that higher levels of HOX genes correlate with ACC and aggressive disease. Analysis of overall and disease-free survival between ACC patients with high and low HOX gene expression showed a correlation between high HOX levels and poor prognosis (Fig. 5e, Supplementary Figs. S8 and S9). These data argue that HOX genes can be drivers of aggressive ACC disease.

**Fig. 6:**
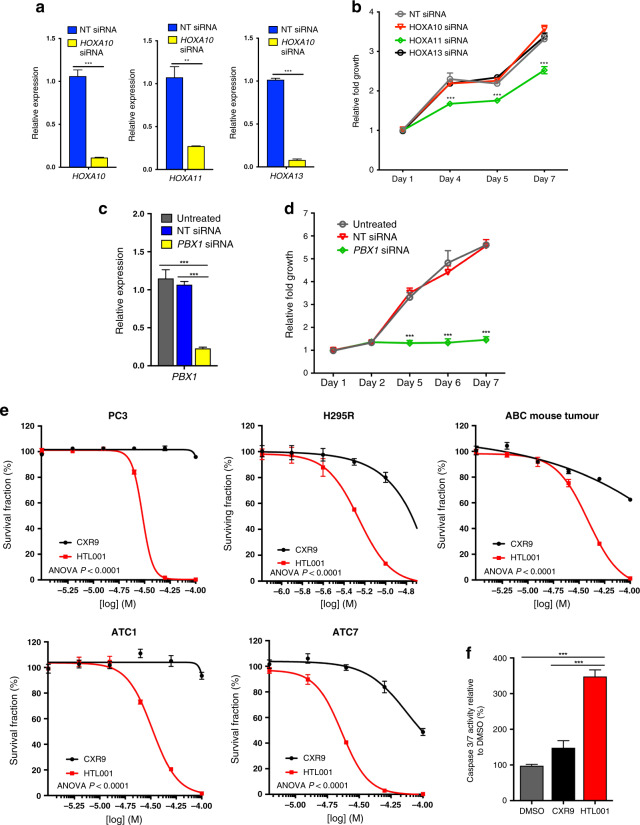
ACC cells are sensitive to inhibition of HOX–PBX function. **a** qRT-PCR of *HOXA10*, *HOXA11* or *HOXA13* in H295R cells treated with a non-targeting (NT) siRNA or a siRNA targeting *HOXA10*, *HOXA11* or *HOXA13*. The data represent the mean ± SD from three biological repeats. **b** Growth curve of H295R cells treated with a NT siRNA or a siRNA targeting *HOXA10*, *HOXA11* or *HOXA13*. **c** qRT-PCR of *PBX1* in untreated H295R cells, cells treated with a NT siRNA, or a siRNA targeting *PBX1*. The data represent the mean ± SD from three biological repeats. **d** Growth curve of untreated H295R cells, cells treated with a NT siRNA, or a siRNA targeting PBX1. **e** Survival fraction of PC3, H295R, ABC (*Ctnnb1* mutant) mouse adrenal cells, ATC1 and ATC7 cells treated with HTL001 or CXR9. Cell viability was measured using Cell TitreGlo. **f** Caspase 3/7 activity for H295R cells treated with 5 μM HTL001 (IC50), DMSO or 5 μM CXR9 for 24 h. The data represent the mean ± SD from three biological repeats. One-way ANOVA, **P < 0.01, ***P < 0.001.

To investigate if adrenal tumour growth is dependent on HOX genes, we performed siRNA knockdown studies of HOX genes expressed in H295R cells.^13^ Knockdown of *HOXA11*, but not *HOXA10* or *HOXA13*, led to reduced growth of H295R cells, supporting a role of HOX genes in promoting adrenal tumour cell proliferation (Fig. 6a, b). Analysis of *HOXA11* paralogues after *HOXA11* knockdown showed a modest reduction in *HOXC11* expression, while *HOXD11* expression was not detected in H295R cells in control or siRNA-treated samples (Fig. S4D). Our HOX gene expression correlation analysis showed a strong correlation of HOX genes within clusters, including *HOXA10* with *HOXA11* and *HOXA13*, and weaker associations with their paralogues (Supplementary Tables S7, S8 and Supplementary Fig. S6). Analysis of *HOXA11* expression in the TCGA ACC dataset showed that it significantly correlated with *Ki67* expression (*P* = 0.0023, *r* = 0.337) the proliferation gene signature (*P* = 0.00053, *r* = 0.381) and *CCNE1* (*P* = 0.0010, *r* = 0.363) expression in these tumours.

As we have shown that several HOX genes correlate with proliferation markers and aggressive disease in ACC we chose to target *PBX1*, a transcription factor that cooperates with HOX proteins to regulate target gene expression and has been implicated in adrenal development and function.^15^ siRNA knockdown of *PBX1* in H295R cells led to reduced levels of expression and to a marked reduction in cell proliferation (Fig. 6c, d). H295R cells harbour a *CTNNB1*-activating mutation, and our qRT-PCR studies showed that *PBX1* knockdown had no effect on WNT signalling, as measured by the expression levels of downstream targets *AXIN2* and *LEF1* (Supplementary Fig. S4E). In order to further investigate if HOX factors can act as drug targets in ACC, we used a developed antagonist peptide, which interferes with the interaction between HOX and PBX proteins (HTL001^30^). Our drug response studies showed that H295R cell growth was highly inhibited by HTL001 but not by a control peptide (CXR9) (Fig. 6e). The IC_50_ of HTL001 in H295R cells was lower than that of another responsive cell line, the prostate cancer line PC3 (5.54 μM versus 30.11 μM). To investigate if mouse adrenal tumours were also sensitive, we analysed three models: two cell lines derived from adrenal tumours driven by SV40 T antigen, ATC1 (containing an activating *Ctnnb1* mutation^35^) and ATC7; and primary cells derived from adrenal tissue from the *Ctnnb1* mutant mice described above. These cells were treated with the antagonist HOX–PBX peptide and found to be responsive, with ATC7 cells being the most sensitive (IC50 23.11 μM) (Fig. 6e). Cell death assays on H295R cells confirmed an increase in apoptosis in cells treated with the antagonist (Fig. 6f).

## Discussion

This study investigates the roles of HOX genes in ACC and their potential as drug targets in this disease. HOX genes have been implicated in cancer, most notably in haematological malignancies.^36^ In many cases, elevated expression correlates with poor prognosis, which is what we observe for *HOXB9* and other HOX genes in ACC. Our data show that overexpression of *Hoxb9* in the adrenal leads to an increase in foetal derived X-zone cells, but does not promote hyperplasia or neoplasia. However, in combination with an activated *Ctnnb1* mutation, high levels of *Hoxb9* lead to an increase in proliferation. This data suggests that HOX genes do not initiate neoplasia but can promote aggressive disease with an increase in cell cycle-dependent genes, such as *Cyclin E1*. Our studies also show that adrenal tumour cells are dependent on the HOX–PBX interaction for growth and that therefore these factors have the potential to be drug targets in ACC.

ACC has a sex bias, with females being more at risk than males.^37^ Consistent with this, mice with *Ctnnb1*-activating mutations in the adrenal show a more aggressive and earlier phenotype in females, which we also observe in our *Ctnnb1* mutant mice. Recent data have shown a sex-specific difference in cell proliferation and renewal in the adrenal cortex, with females showing higher turnover and using an additional stem/progenitor compartment found in the capsule.^38^ This difference was driven by testicular androgens that repressed these processes in the male. Our data is consistent with *Hoxb9* promoting the proliferative pathways that are repressed in the male. Our RNA-seq analysis did not show changes in classic androgen receptor targets, such as *Srd5a2*, however, we did observe an increase in *Frzb* expression in double-mutant adrenals, which has been shown to be higher in female adrenals and upregulated in adrenals of castrated mice.^39^

Overexpression of *Hoxb9* led to an increase in *Sf-1* levels both in a normal and neoplastic context. HOX genes have been implicated in the regulation of *Sf-1* transcription in the developing adrenal.^14^ Transgenic mice with increased foetal *Sf-1* expression showed extra-adrenal formation and mice with a Sf-1 sumoylation mutation have a persistent X zone, suggesting that the bigger foetal derived X zone seen in the adrenal of the transgenic Sf-1:Hoxb9 mice could be due to the higher *Sf-1* levels.^40,41^ Whether this pathway is active in adrenal tumours is unclear as we did not observe an increase in Sf-1 protein in the adrenals of double-mutant mice.

Our data show an increase in cell cycle markers in double-mutant male tumours including *Ccne1*, a gene commonly amplified in ACC and thought to be a disease driver. Both HOX genes and SF-1 have been implicated in the regulation of *CCNE1* expression.^42,43^ A surprising result was the observation of an increase in the expression of the FOS/JUN/EGR1 pathway without an increase in *Cyp11b2* expression.^34^ Analysis of Fosb staining in normal adrenals did show its association with the ZG, the site of angiotensin signalling, but was also found in a few capsule cells (Supplementary Fig. S10). This suggests that the FOS/JUN family can be activated in non-steroidogenic cells of the adrenal. Whether its function in these cells is associated with cell proliferation is not known.

WNT signalling has been implicated in adrenal development and differentiation, as well as homoeostasis and neoplasia in the adult. The double-mutant animals in our study do not show major changes in differentiation, such as *Cyp11b1* or *Cyp11b2*, or stem cell markers, such as *Shh* and *Gli1*, when compared to the *Ctnnb1* mutants. The major difference we observe is an increase in proliferation in double-mutant male adrenals. Mice with *Znrf3* mutations show an increase in proliferation of the ZF, which is thought to be due to moderate rather than high WNT signalling activation.^44^ We do not observe any major changes in WNT target genes in the neoplastic tissues, suggesting that HOX genes do not act to modulate this pathway in adrenal disease.

Our analysis of HOX gene expression revealed various members of the family had increased levels in human ACC, some of which correlated with increased proliferation markers. Our experiments in H295R cells identified a need for *HOXA11*, but not for other HOX expressed genes, *HOXA10* and *HOXA13*, for survival. This variety prompted us to propose a more general HOX-based therapy for patient treatment as different tumours may have specific HOX gene combinations driving disease progression. Studies have shown that functional redundancy in organ formation can be observed in paralogous members of the HOX gene family.^45^ Our expression studies in ACC samples comparing members within the HOX family showed that correlations were higher in genes of the same cluster rather than their related paralogues. Consistent with this, analysis of the *HOXA11* paralogues in H295R cells with *HOXA11* knockdown only showed a mild reduction in *HOXC11* expression. HOX transcript antisense intergenic RNA (HOTAIR) has been implicated in ACC with high expression correlating with worse disease outcome.^46^ HOTAIR has been proposed to regulate the expression of many genes through its interaction with polycomb repressive complex 2. How this LncRNA interacts with HOX genes in ACC is not known.

Our studies show that adrenal tumour cells are dependent on HOX genes for their growth, although we were not able to predict which member of the family is the most relevant to patients. As a way to overcome this issue, we determined a dependency of adrenal tumour cells on the interaction between HOX and PBX factors for growth. This makes inhibiting this interaction a promising therapy option for ACC. Peptide-based therapeutics are being used in cancer treatment and a variant of HTL001 is being developed for clinical trials currently, as in vivo studies in mice showed growth inhibition in a range of tumour types, and the drug was well-tolerated.^47^ We treated various adrenal tumour models with the HOX peptide inhibitor and saw an effect on cell survival. This suggests that this therapy would be effective in ACC with different genetic backgrounds.

## Supplementary information

Supplementary information

## Data Availability

RNA-seq data can be found in the supplementary tables.
