# Supplementary material for: HOX genes promote cell proliferation and are potential therapeutic targets in adrenocortical tumours
Source: Br J Cancer. 2020 Nov 20;124(4):805–16. doi: 10.1038/s41416-020-01166-z (PMC7884796; doi:10.1038/s41416-020-01166-z)
Supplement: Supplementary file 1 — Supplementary information [file 41416_2020_1166_MOESM1_ESM.pdf]

Supplementary methods

Immunohistochemistry

Mouse tissues were fixed overnight in 4% paraformaldehyde (PFA), dehydrated in an ethanol gradient series, washed in Histoclear and embedded in wax. Sections were cut at 4 µm, rehydrated and antigen retrieval was obtained by gently boiling sections for 20 minutes in the appropriate buffer, see below, and then cooled. Sections were treated with 3% H_2O_2 to block endogenous peroxidase activity, washed with PBS and blocked in 10% sheep serum. Primary antibodies in 2.5% horse serum were incubated overnight at 4°C. Anti-mouse or anti-rabbit ImmPRESS HRP detection kits (Vector Laboratories), or anti-sheep VisUCyte HRP detection reagent (Bio-Techne) were used according to manufacturer's instructions. Staining was carried out using DAB chromogen (Sigma) and sections were counterstained with haematoxylin. For all stains, sections were processed from at least three animals of each genotype. The following antibodies were processed for antigen retrieval in citrate buffer (0.1 M sodium citrate pH6, 0.05% Tween); Ki67 (Abcam ab16667), Hoxb9 (Santa Cruz sc-398500), Sf-1 (Abcam ab65815), DAB2 (BD Transduction Labs 610464), Tyrosine Hydroxylase (Merck AB152), 20α HSD (a kind gift from Yacob Weinstein), β-catenin (BD Transduction Labs 610153), Lef1 (Cell Signalling Technology 2230), cleaved Caspase 3 (Abcam ab2302), Fosb (Cell Signalling Technology 2251). Antigen retrieval for Cyp11b1 (2625, a kind gift from Celso Gomez-Sanchez) and Cyp11b2 (2084, a kind gift from Celso Gomez-Sanchez) antibodies was 1 mM EDTA, 10 mM Diethanolamine pH 9.

Taqman gene expression probes

Gapdh Mm99999915_g1, Hoxb9 Mm01700220_m1, Sf-1/Nr5a1 Mm00446826_m1, Shh Mm00436528_m1, Ptch1 Mm00436026_m1, Axin2 Mm00443610_m1, Cyp11b1

Mm01204952_m1, Cyp11b2 Mm01204955_g1, Akr1c18 Mm00506289_m1, Pik3c2g Mm00440781_m1, Cdk1 Mm00772472_m1, Ccnb1 Mm03053893_gH, Ccnb2 Mm01171453_m1, Ccne1 Mm01266311_m1, Knstrn Mm00518200_m1, Fos Mm00487425_m1, Fosb Mm00500401_m1, JunB Mm00492781_s1, GAPDH Hs03929097_g1, PBX1 Hs00231228_m1, HOXA10 Hs00538183_m1, HOXA11 Hs00194149_m1, HOXA13 Hs00426284_m1, LEF1 Hs01547250_m1, AXIN2 Hs00610344_m1.

Supplementary data

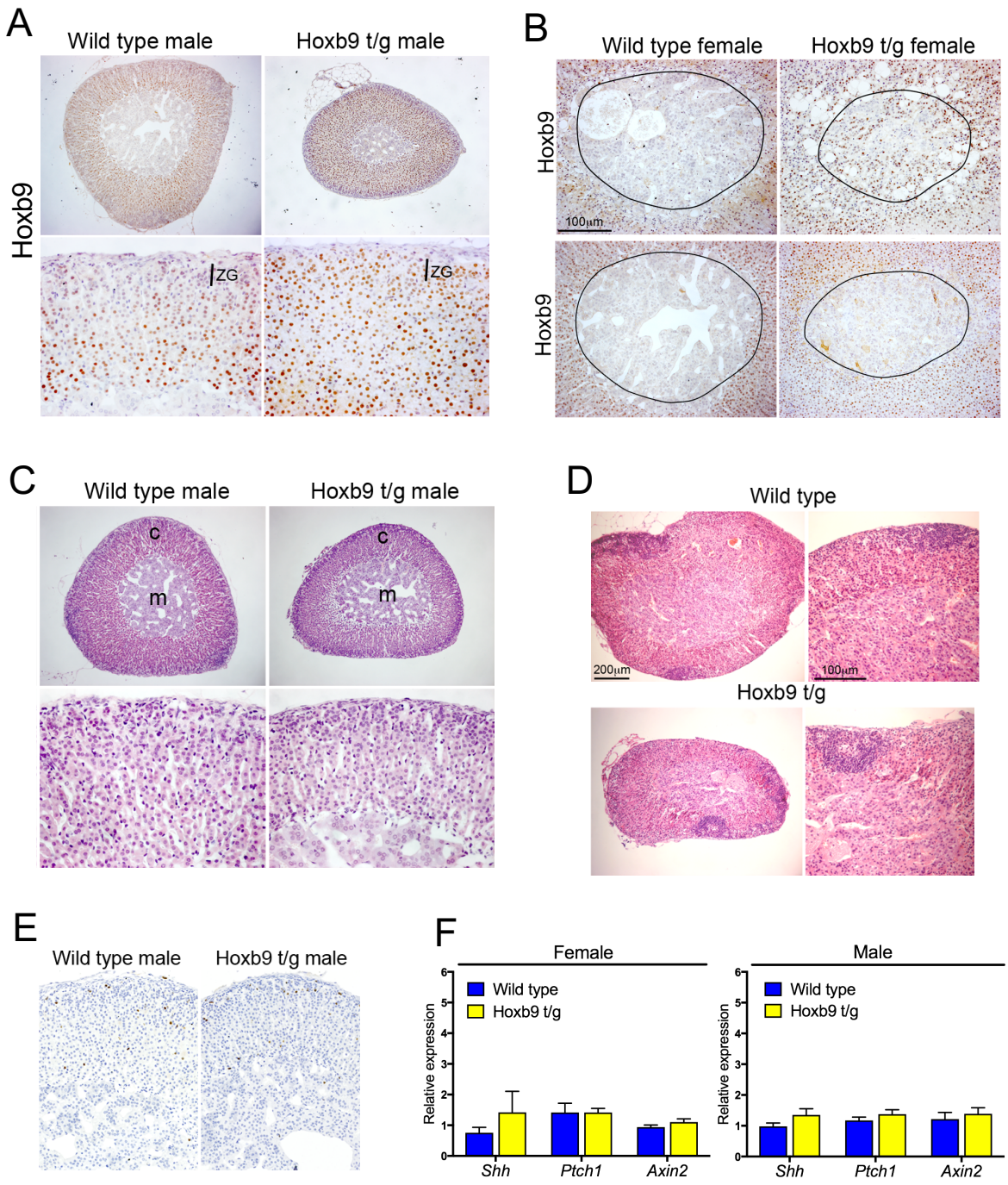

Figure S1. (A) Hoxb9 immunohistochemistry on sections of male wild type and Hoxb9 t/g adrenal glands. ZG is zona glomerulosa. (B) Hoxb9 expression in the medulla of 3-month-old female wild type and Hoxb9 t/g adrenal glands. The medulla is highlighted with a black line. (C) Haematoxylin and eosin (H & E) stain on sections from wild type and Hoxb9 t/g adrenal glands. c is cortex, m is medulla. (D) H & E stain of sections from wild type and Hoxb9 transgenic 18-month-old male adrenal glands. (E) Ki67 immunohistochemistry on sections of wild type and Hoxb9 t/g male adrenal glands. (F) qRT-PCR of *Shh*, *Ptch1* and *Axin2* on wild type and Hoxb9 t/g adrenal glands. Hoxb9 t/g indicates Sf1:Hoxb9 transgenic.

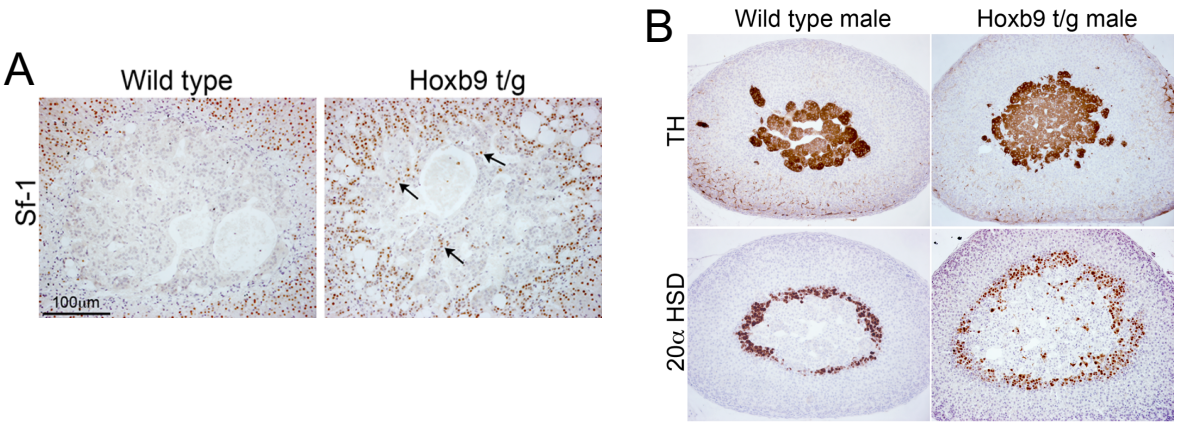

Figure S2. (A) Sf-1 IHC stain on sections of wild type and Hoxb9 t/g female adrenals from 3-month-old animals. Arrows indicate positive cells in the medulla. (B) TH and 20α-HSD IHC stains on sections of wild type and Hoxb9 t/g male adrenal glands from 3-week-old animals.

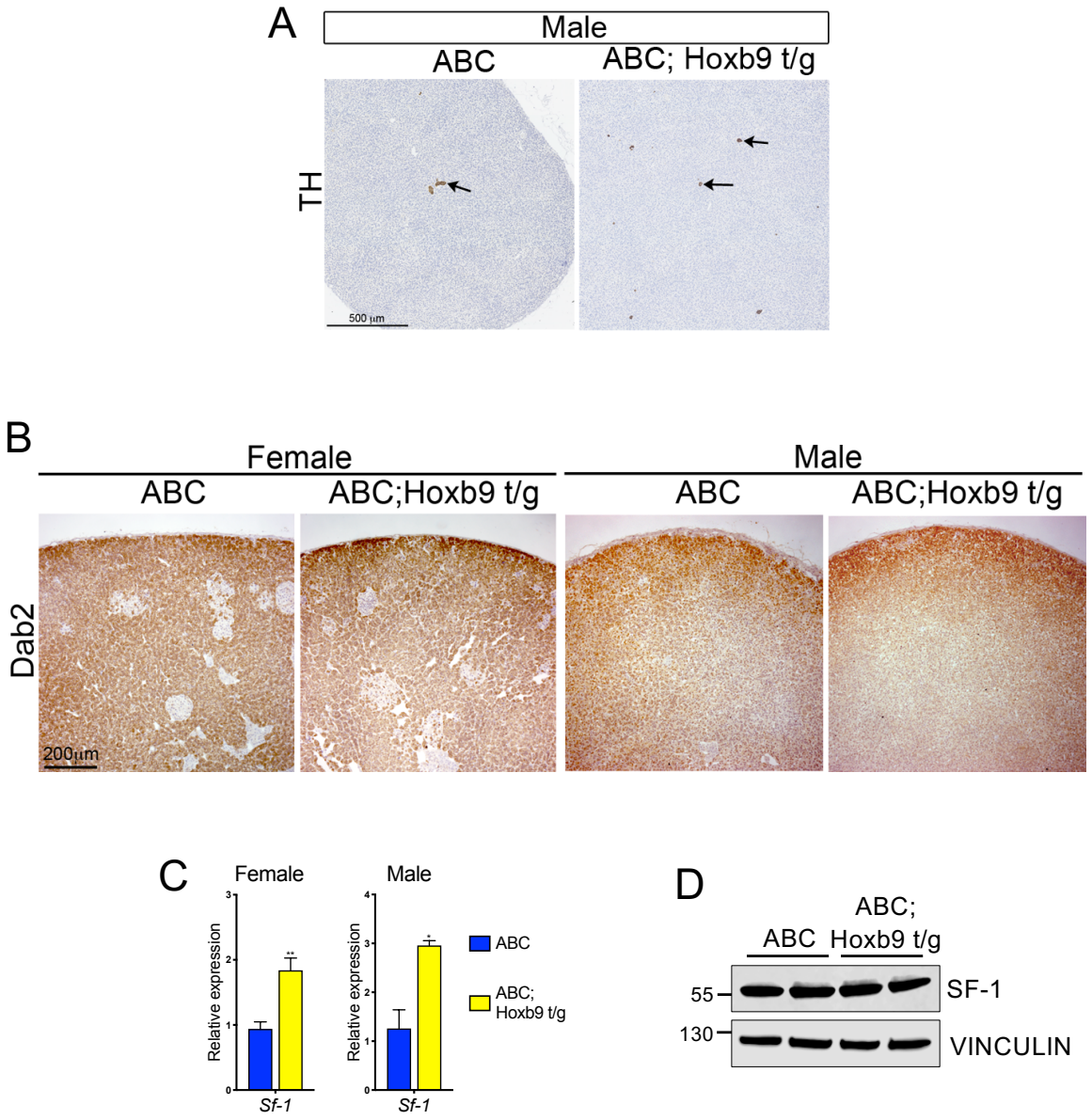

Figure S3. (A) Medulla marker TH stains on sections of ABC and ABC; Hoxb9 t/g male 3-month-old adrenal tumours. Arrows indicate few positive cells in these tumours. (B) ZG marker Dab2 stains on sections of ABC and ABC; Hoxb9 t/g 3-month-old adrenals showing expression throughout the tumours. (C) qRT-PCR of *Sf-1* on ABC and ABC; Hoxb9 t/g adrenal tumours. The data represent mean ± SD from three biological repeats. Students t-test, ** $p < 0.01$, * $p < 0.05$. (D) Western blot analysis of SF-1 on ABC and ABC; Hoxb9 t/g adrenal tumours from female animals. Adrenals from 2 animals of each genotype are shown. Vinculin is used as a loading control. ABC indicates Ctnnb1 mutant tumours, ABC; Hoxb9 t/g indicates double mutant tumours.

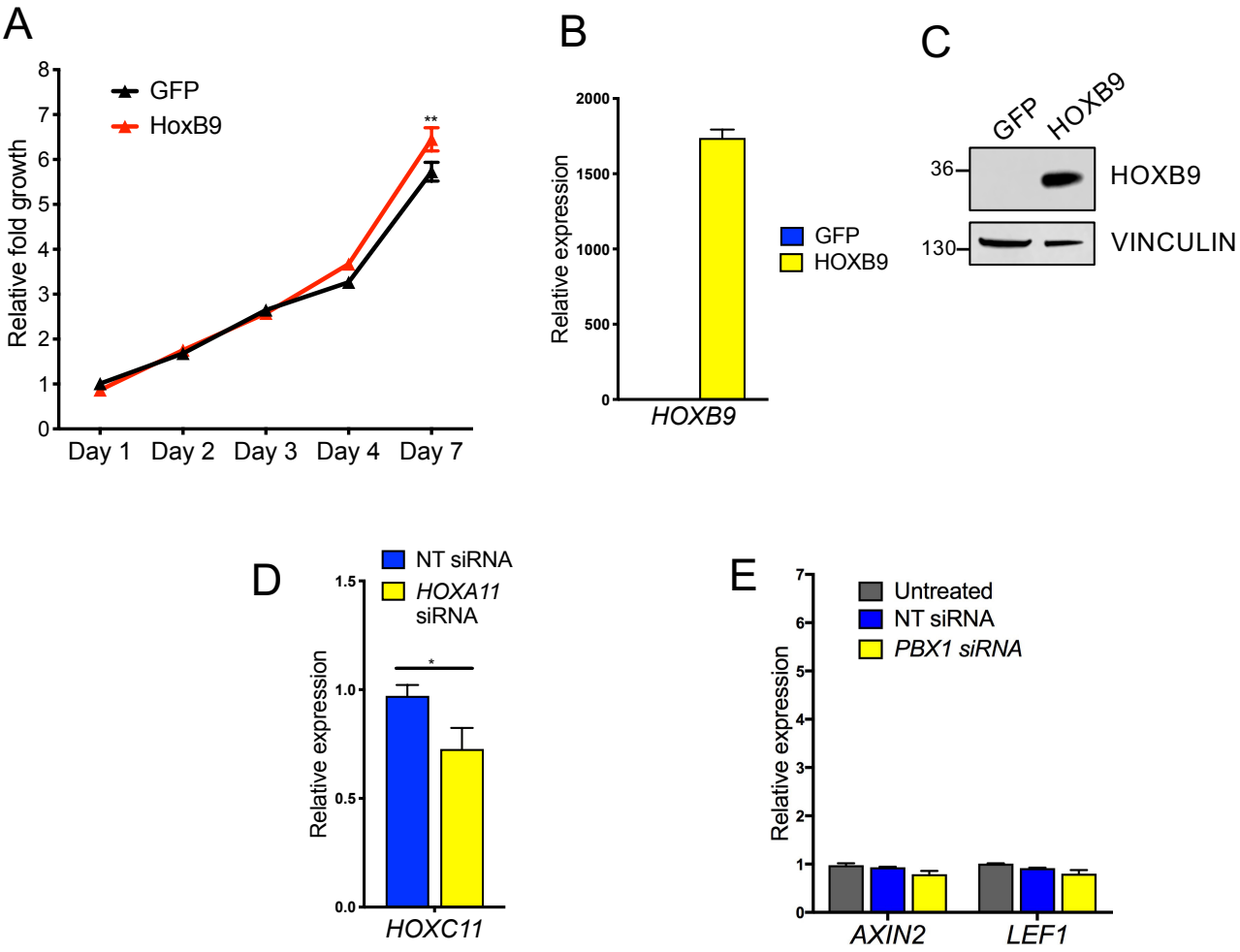

Figure S4. (A) Growth curve of H295R cells expressing GFP or HOXB9. The data represent mean ± SD from three biological repeats. One-way ANOVA, ** $p < 0.01$. (B) qRT-PCR of *HOXB9* in H295R cells expressing GFP or HOXB9. (C) Western blot analysis of HOXB9 in H295R cells expressing GFP or HOXB9. Vinculin is used as a loading control. (D) qRT-PCR of *HOXC11* in H295R cells treated with a NT siRNA or an siRNA targeting *HOXA11*. * $p < 0.05$. (E) qRT-PCR of *AXIN2* and *LEF1* in H295R cells untreated, treated with a NT siRNA or an siRNA targeting *PBX1*. The data represent mean ± SD from three biological repeats.

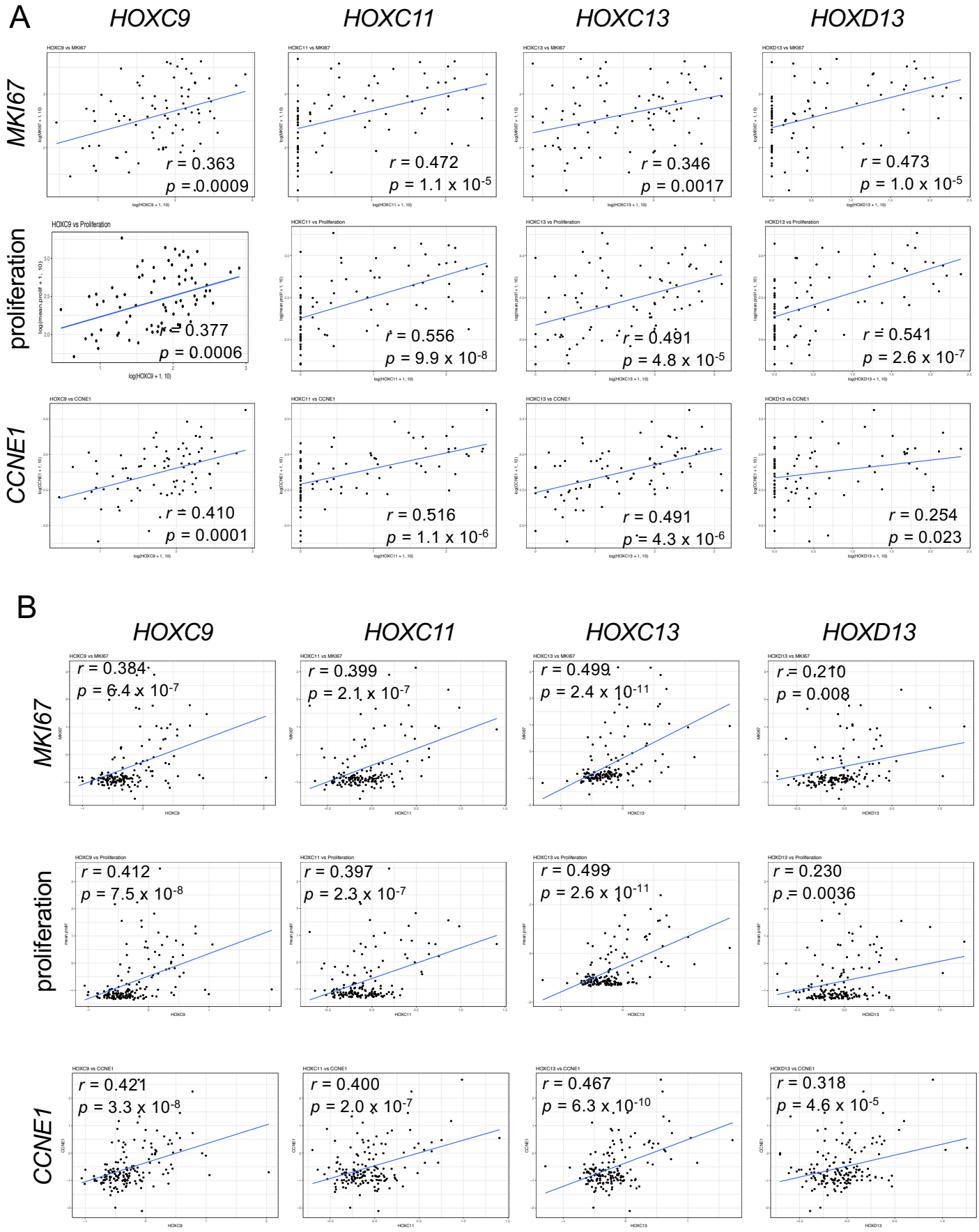

Figure S5. (A) TCGA cohort HOX gene correlations with *MKI67*, the proliferation gene signature and *CCNE1*. (B) Cochin cohort HOX gene correlations with *MKI67*, the proliferation gene signature and *CCNE1.*

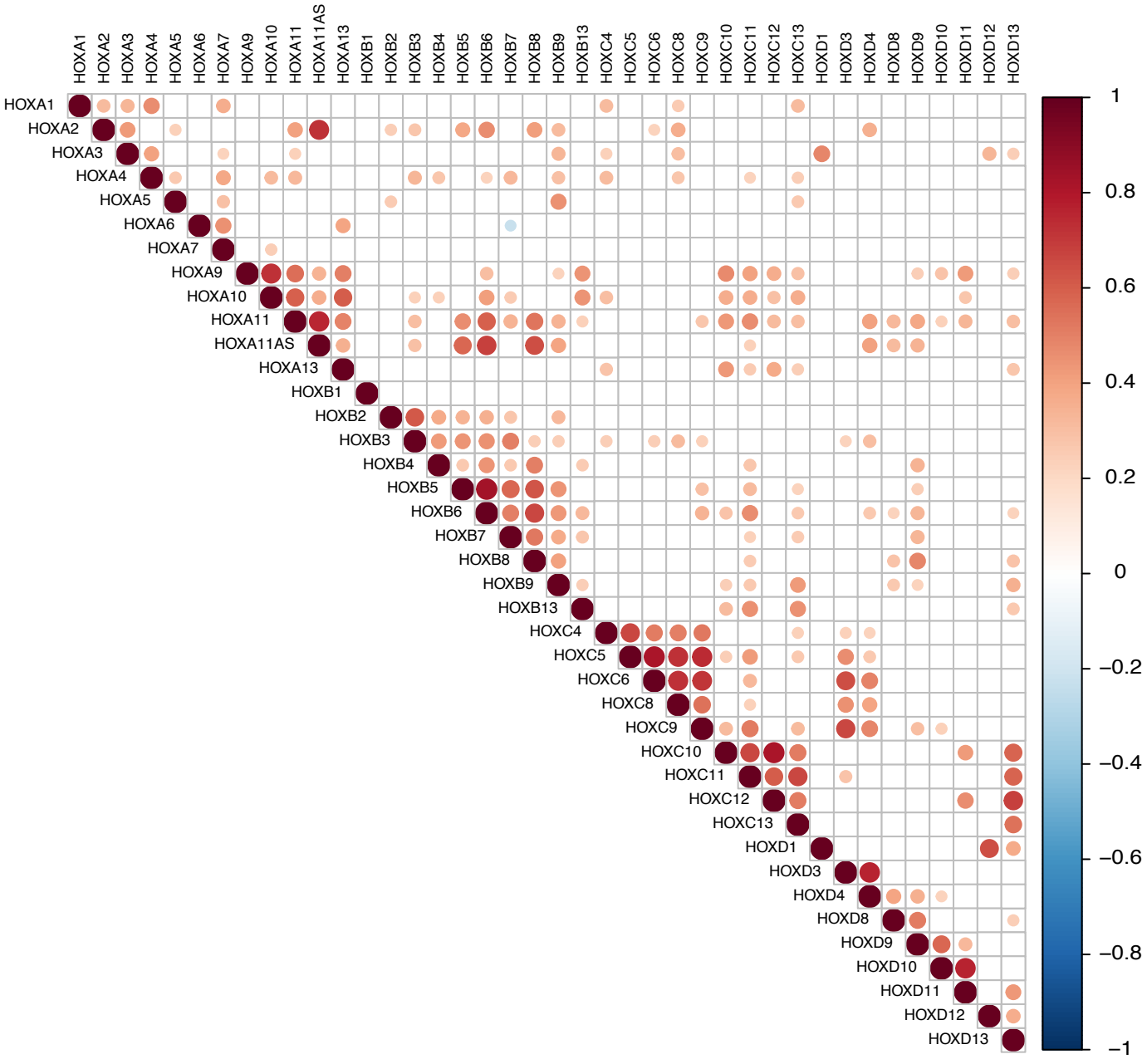

Figure S6. Correlogram of HOX gene expression correlation with the expression of other HOX genes in ACC TCGA patient samples. Colour scale is the correlation r value and spot size is the p value.

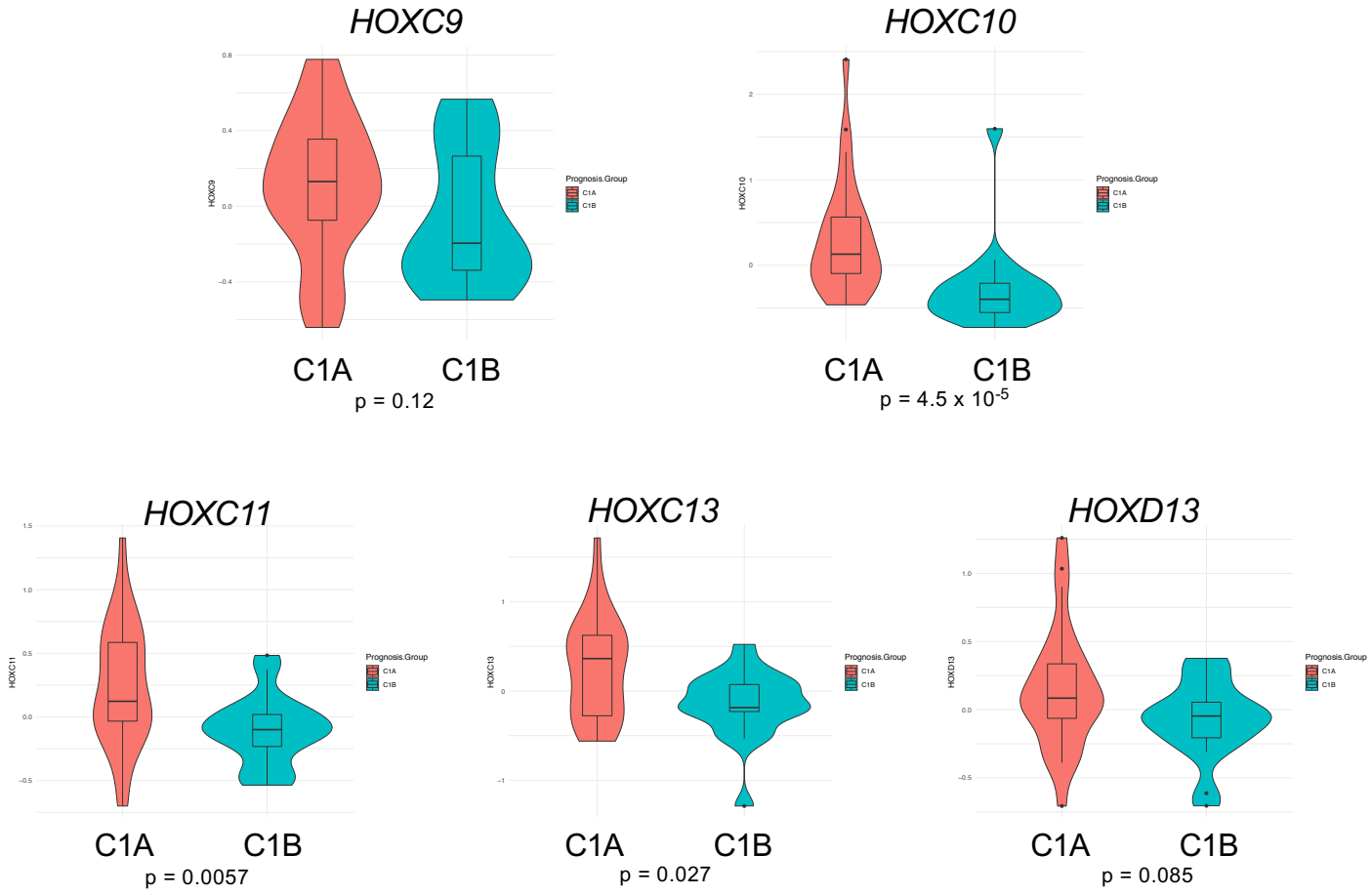

Figure S7. Cochin cohort HOX gene expression in C1A and C1B sub-types.

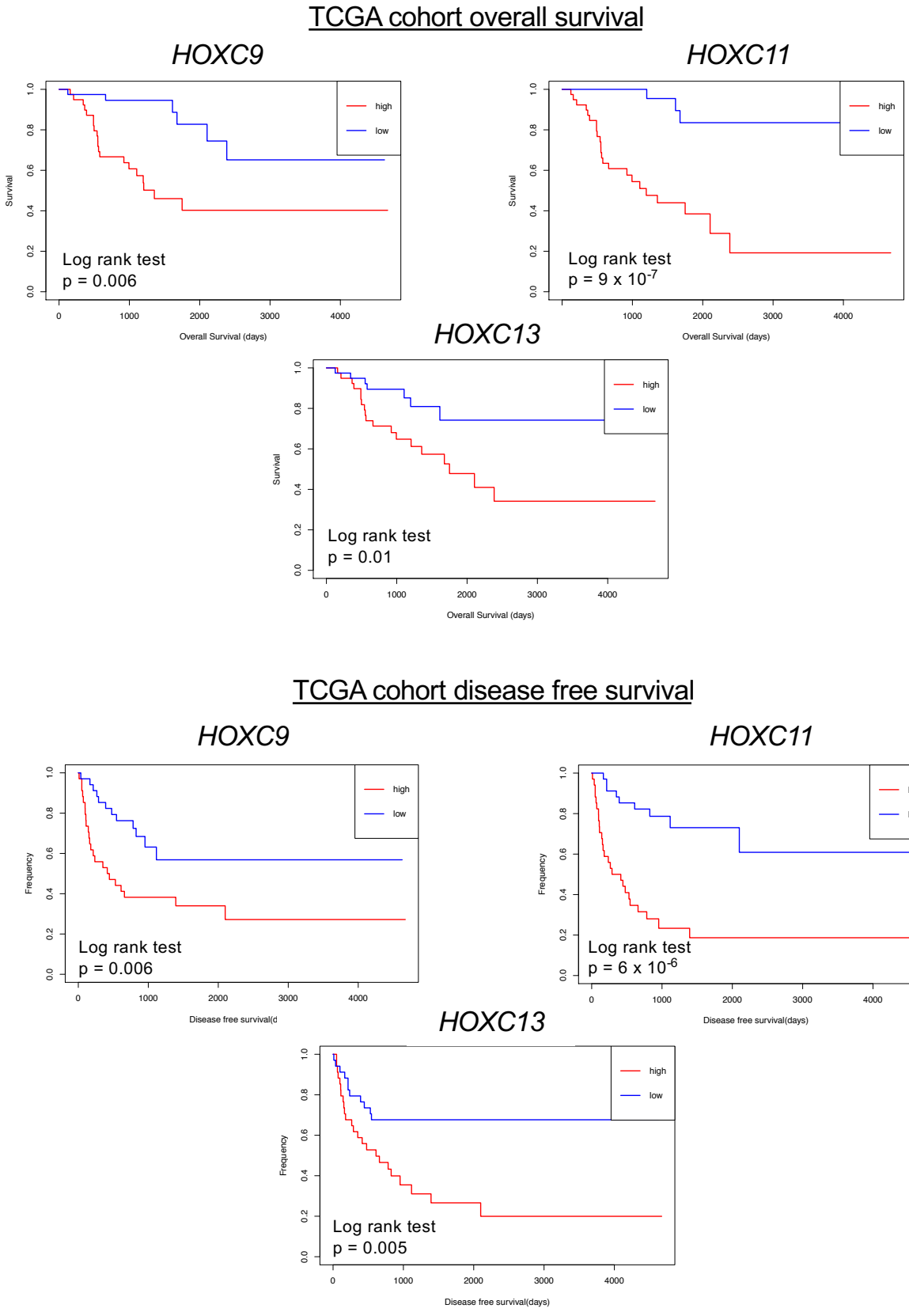

Figure S8. Kaplan Meier survival curves for ACC patients from the TCGA cohort that had either high or low *HOXC9*, *HOXC11* or *HOXC13* expression.

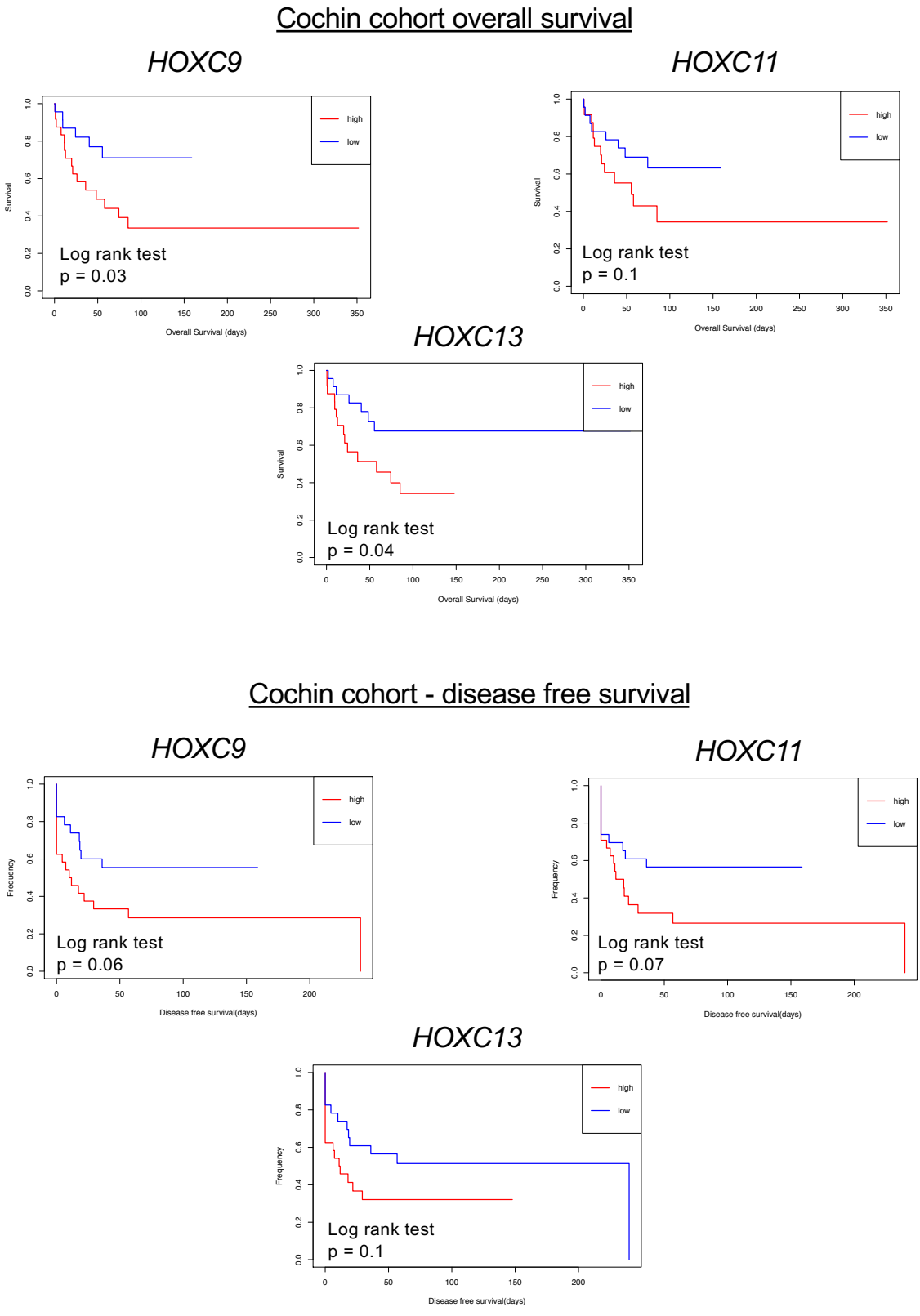

Figure S9. Kaplan Meier survival curves for ACC patients from the Cochin cohort that had either high or low *HOXC9*, *HOXC11* or *HOXC13* expression.

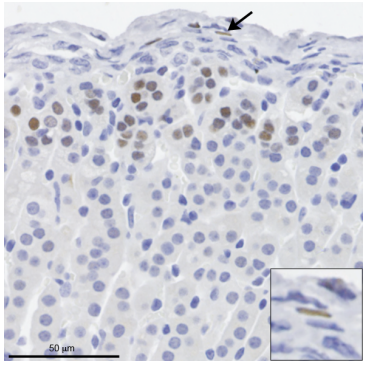

Figure S10. Fosb staining on a section of a 3-month-old male adrenal gland. Arrow indicates a positive cell in the capsule that is also shown at high magnification.

Table S1. Genes used for the proliferation signature

Probe Set ID	Gene Symbol	Gene Name
201291_s_at	TOP2A	Topoisomerase (DNA) II alpha 170kDa
201292_at	TOP2A	Topoisomerase (DNA) II alpha 170kDa
201890_at	RRM2	Ribonucleotide reductase M2
202095_s_at	BIRC5	Baculoviral IAP repeat-containing 5
202503_s_at	KIAA0101	KIAA0101
202580_x_at	FOXM1	Forkhead box M1
202589_at	TYMS	Thymidylate synthetase
202705_at	CCNB2	Cyclin B2
202870_s_at	CDC20	Cell division cycle 20 homolog (S. cerevisiae)
202954_at	UBE2C	Ubiquitin-conjugating enzyme E2C
203213_at	CDK1	Cyclin-dependent kinase 1
203214_x_at	CDK1	Cyclin-dependent kinase 1
203362_s_at	MAD2L1	MAD2 mitotic arrest deficient-like 1 (yeast)
203554_x_at	PTTG1	Pituitary tumor-transforming 1
203755_at	BUB1B	Budding uninhibited by benzimidazoles 1 homolog beta (yeast)
203764_at	DLGAP5	Discs, large (Drosophila) homolog-associated protein 5
204026_s_at	ZWINT	ZW10 interactor
204033_at	TRIP13	Thyroid hormone receptor interactor 13
204092_s_at	AURKA	Aurora kinase A
208079_s_at	AURKA	Aurora kinase A
204162_at	NDC80	NDC80 homolog, kinetochore complex component (S. cerevisiae)
204170_s_at	CKS2	CDC28 protein kinase regulatory subunit 2
204444_at	KIF11	Kinesin family member 11
204641_at	NEK2	NIMA (never in mitosis gene a)-related kinase 2
204822_at	TTK	TTK protein kinase
204825_at	MELK	Maternal embryonic leucine zipper kinase
204962_s_at	CENPA	Centromere protein A
205034_at	CCNE2	cyclin E2
205046_at	CENPE	Centromere protein E, 312kDa
206102_at	GINS1	GINS complex subunit 1 (Psf1 homolog)
206364_at	KIF14	Kinesin family member 14
207828_s_at	CENPF	Centromere protein F, 350/400kDa (mitosin)
209172_s_at	CENPF	Centromere protein F, 350/400kDa (mitosin)
209408_at	KIF2C	Kinesin family member 2C
209642_at	BUB1	Budding uninhibited by benzimidazoles 1 homolog (yeast)
209714_s_at	CDKN3	Cyclin-dependent kinase inhibitor 3
209773_s_at	RRM2	Ribonucleotide reductase M2
210052_s_at	TPX2	TPX2, microtubule-associated, homolog (Xenopus laevis)
210559_s_at	CDK1	Cyclin-dependent kinase 1
212022_s_at	MKI67	Antigen identified by monoclonal antibody Ki-67
212949_at	NCAPH	Non-SMC condensin I complex, subunit H
213226_at	CCNA2	Cyclin A2
214710_s_at	CCNB1	Cyclin B1
218009_s_at	PRC1	Protein regulator of cytokinesis 1
218039_at	NUSAP1	Nucleolar and spindle associated protein 1
218355_at	KIF4A	Kinesin family member 4A
218542_at	CEP55	Centrosomal protein 55kDa
218585_s_at	DTL	Denticleless homolog (Drosophila)
218662_s_at	NCAPG	Non-SMC condensin I complex, subunit G
218663_at	NCAPG	Non-SMC condensin I complex, subunit G
218726_at	HJURP	Holliday junction recognition protein
218755_at	KIF20A	Kinesin family member 20A
218883_s_at	MLF1IP	MLF1 interacting protein
219148_at	PBK	PDZ binding kinase
219306_at	KIF15	Kinesin family member 15
219918_s_at	ASPM	asp (abnormal spindle) homolog, microcephaly associated (Drosophila)
220651_s_at	MCM10	Minichromosome maintenance complex component 10
221436_s_at	CDCA3	Cell division cycle associated 3
221520_s_at	CDCA8	Cell division cycle associated 8
222039_at	KIF18B	Kinesin family member 18B
222077_s_at	RACGAP1	Rac GTPase activating protein 1

Table S2. Mouse adrenal tumours analysed by RNAseq

Animal ID	Sex	Genotype
184208-Turq182	Female	ABC
183159-B200	Female	ABC
B505	Female	ABC
207386	Female	ABC
153514	Female	ABC; Hoxb9
153516	Female	ABC; Hoxb9
189961-B532	Female	ABC; Hoxb9
191284	Female	ABC; Hoxb9
166836	Male	ABC
166838	Male	ABC
182098-Turq199	Male	ABC
169229	Male	ABC; Hoxb9
182083-Turq191	Male	ABC; Hoxb9
182086-Turq194	Male	ABC; Hoxb9

Table S3. Genes differentially expressed in ABC_Hoxb9 Female vs ABC Female

								Read count							
Gene	ENSEMBL_Gene_ID	baseMean	log2FoldChange	lfcSE	stat	pvalue	padj	184208_Turq1	183159_B200_	B505_Normali	207386_Norm	153514_Norm	153516_Norm	189961_B532_	191284_Norm
Slc27a2	ENSMUSG00000027359	21.9051591	4.191942937	1.148305906	3.650545482	0.000261684	0.085256787	0	1	2	5	14	20	130	2
Prg4	ENSMUSG00000006014	10.56221999	3.687436402	1.012912294	3.640430098	0.000272183	0.085256787	2	1	1	2	8	2	30	39
Apol7c	ENSMUSG00000044309	6.58620907	3.28113107	0.876188625	3.744777068	0.000180554	0.064025139	2	0	1	2	10	20	6	11
Trim12a	ENSMUSG00000066258	95.97699655	3.243870079	0.734084608	4.418932152	9.91898E-06	0.010965722	22	14	28	10	345	45	276	29
Ptprq	ENSMUSG00000035916	20.79260951	3.047391271	0.764611306	3.98554304	6.7326E-05	0.032737491	1	12	2	4	25	47	13	63
Foxl2	ENSMUSG00000050397	18.98663136	3.015393916	0.803884277	3.751029847	0.00017611	0.064025139	1	8	0	8	32	55	15	33
Tchh	ENSMUSG00000052415	31.44739379	2.76969431	0.702768844	3.941117105	8.1103E-05	0.035360644	4	15	6	8	110	15	71	23
Cfap54	ENSMUSG00000020014	944.4184571	2.611899487	0.462859836	5.642959889	1.67151E-08	5.23574E-05	220	226	389	226	2849	1038	2004	602
Nr6a1	ENSMUSG00000063972	592.0603564	2.59182928	0.414526232	6.252509685	4.03909E-10	2.53035E-06	207	228	151	89	1053	1380	432	1196
Bdnf	ENSMUSG00000048482	15.68834789	2.568158658	0.627518535	4.092562235	4.26633E-05	0.025657524	2	5	6	5	35	25	9	39
Egfem1	ENSMUSG00000063600	12.77949348	2.458183988	0.532236783	4.618590947	3.86355E-06	0.005585499	6	3	4	3	18	28	17	24
Dnase1l3	ENSMUSG00000025279	36.32121221	1.873319968	0.314700492	5.952707473	2.63742E-09	1.23919E-05	17	16	14	16	66	65	50	47
Pex5l	ENSMUSG00000027674	141.7561673	1.788716732	0.412409503	4.337234523	1.44287E-05	0.012912968	69	31	51	102	233	185	134	328
Slc6a5	ENSMUSG00000039728	184.6835041	1.689560986	0.358915349	4.707408006	2.50886E-06	0.00471516	78	79	122	71	193	477	240	219
Cd3e	ENSMUSG00000032093	12.68089399	1.679113556	0.463407924	3.62340277	0.000290753	0.089580378	7	7	5	5	16	23	20	18
Dsp	ENSMUSG00000054889	1085.754275	1.635048749	0.254713327	6.419172365	1.37017E-10	2.4443E-06	627	527	439	523	1190	2168	1323	1890
Serpina3f	ENSMUSG00000066363	16.46395617	1.632374878	0.430623559	3.790723578	0.000150209	0.060064445	6	9	9	7	20	23	23	34
Stard9	ENSMUSG00000033705	536.9815802	1.469829889	0.316412575	4.64529543	3.3959E-06	0.005385956	454	211	268	205	808	1024	689	636
Arhgap20	ENSMUSG00000053199	79.52185178	1.392139913	0.317315969	4.387235592	1.148E-05	0.011647653	37	30	64	43	114	145	84	119
Zbtb16	ENSMUSG00000066687	112.1571643	1.349645638	0.283976259	4.75267067	2.00747E-06	0.004192047	72	60	73	48	158	225	140	123
Nlrc5	ENSMUSG00000074151	82.73442601	1.280321807	0.29981152	4.270422319	1.95103E-05	0.015942474	75	31	41	45	110	112	133	115
Pdgfra	ENSMUSG00000029231	859.0140792	1.274687466	0.304411773	4.187379002	2.82194E-05	0.019642819	526	705	310	470	1159	1286	856	1559
Atp10d	ENSMUSG00000046808	80.59708051	1.258358531	0.344924793	3.648211314	0.000264072	0.085256787	67	27	32	63	129	101	99	126
Adam12	ENSMUSG00000054555	58.85155406	1.193994304	0.331918089	3.597255897	0.000321592	0.094437519	29	38	28	49	54	79	112	83
Cxcl9	ENSMUSG00000029417	56.13421237	1.183284704	0.314522196	3.762165975	0.000168448	0.064025139	29	57	26	27	74	71	82	83
Serpina3g	ENSMUSG00000041481	85.85680109	1.174701512	0.283482245	4.143827462	3.41557E-05	0.022251222	38	47	49	77	118	140	99	119
Gm4070	ENSMUSG00000078606	182.1933688	1.142427675	0.249652429	4.576072746	4.73786E-06	0.006360237	157	94	109	93	299	215	263	228
Cers6	ENSMUSG00000027035	80.36201981	1.109977474	0.300500927	3.693757237	0.000220965	0.076903922	53	56	55	40	85	114	82	158
Filip1	ENSMUSG00000034898	1076.60538	1.10708143	0.254310664	4.353263895	1.34126E-05	0.012603776	538	668	764	760	1005	1363	1548	1966
Gvin1	ENSMUSG00000045868	187.160909	1.069610302	0.262522575	4.074355516	4.61419E-05	0.025657524	166	108	103	106	333	211	251	220
Nr5a1	ENSMUSG00000026751	23101.31731	1.046153797	0.257144039	4.06835717	4.73458E-05	0.025657524	12249	15538	14523	17988	33572	39728	19701	31512
Pgm2l1	ENSMUSG00000030729	128.3059677	1.044079497	0.264608681	3.94574922	7.95508E-05	0.035360644	86	75	108	66	148	192	215	136
Itih5	ENSMUSG00000025780	1828.633184	1.034868495	0.25403474	4.073728243	4.62665E-05	0.025657524	1200	1353	1399	846	2310	2314	1905	3301
Tenm4	ENSMUSG00000048078	1271.834504	1.017366294	0.26614314	3.822628288	0.000132037	0.053945621	1134	964	690	577	2102	1482	1684	1542
Cdon	ENSMUSG00000038119	189.7879037	0.915018321	0.19708776	4.642694802	3.43894E-06	0.005385956	125	106	142	152	236	221	251	285
Vcam1	ENSMUSG00000027962	686.1843965	0.901959428	0.235112037	3.836296263	0.000124904	0.052165345	458	374	509	572	876	1183	709	808
Flnc	ENSMUSG00000068699	166.2070425	0.901711554	0.240700361	3.746199432	0.000179534	0.064025139	138	106	97	123	193	174	216	283
Tnfrsf21	ENSMUSG00000023915	129.3081305	0.898144543	0.23955356	3.749243145	0.000177369	0.064025139	106	68	75	112	193	164	148	169
Tmem181b-ps	ENSMUSG00000096780	285.7006728	0.895979863	0.211719068	4.231928054	2.31697E-05	0.016748095	179	190	246	183	385	442	294	366
Dock8	ENSMUSG00000052085	264.9339641	0.831284386	0.209374408	3.970324719	7.17747E-05	0.033723363	244	168	157	193	348	309	387	314
Tgfbr3	ENSMUSG00000029287	3527.69397	0.822988846	0.202464791	4.064849213	4.80635E-05	0.025657524	2766	1822	3052	2550	4028	4489	4715	4800
Kank1	ENSMUSG00000032702	394.654745	0.797933226	0.184619767	4.322035714	1.54596E-05	0.013206728	250	296	259	349	531	488	433	551
Sfxn5	ENSMUSG00000033720	228.116906	0.777478386	0.213565554	3.640467156	0.000272144	0.085256787	191	125	188	167	245	284	340	286
Msr1	ENSMUSG00000025044	78.52088009	0.743620497	0.207709181	3.580104121	0.000343457	0.097860037	63	54	64	53	108	97	95	94
Kif21a	ENSMUSG00000022629	606.8049681	0.656018112	0.164687737	3.983405962	6.79346E-05	0.032737491	468	565	440	412	741	751	805	671
Klhl12	ENSMUSG00000026455	805.6268982	0.597117643	0.120380972	4.960232762	7.04088E-07	0.001654078	674	601	644	646	988	1029	906	957
Cyyr1	ENSMUSG00000041134	583.3518596	0.587586018	0.16059243	3.658864967	0.000253335	0.085020959	520	384	505	454	675	756	659	713
Thnsl2	ENSMUSG00000054474	490.4863057	-0.775627118	0.206034003	-3.764558795	0.000166843	0.064025139	495	785	635	562	415	329	372	330
1700040L02Rik	ENSMUSG00000019945	141.0419481	-0.895256479	0.204316839	-4.38170677	1.17753E-05	0.011647653	185	194	196	160	82	99	92	121
Insc	ENSMUSG00000048782	124.1928239	-1.024209528	0.28460156	-3.598748818	0.000319752	0.094437519	198	115	211	141	89	60	94	87
Skap1	ENSMUSG00000057058	529.0237922	-1.045117196	0.252283536	-4.142629408	3.43347E-05	0.022251222	660	931	574	686	455	320	348	258
Sbspon	ENSMUSG00000032719	899.2799666	-1.079379755	0.254205857	-4.24608531	2.17538E-05	0.016353624	1544	1070	1202	1066	568	495	807	441
Gstm7	ENSMUSG00000044035	1138.044699	-1.170350479	0.326918171	-3.579949305	0.000343661	0.097860037	964	2486	1078	1776	767	630	765	639
RP23-82I13.8	ENSMUSG00000107432	69.66141325	-1.231228805	0.275430358	-4.470200064	7.81465E-06	0.009179279	68	114	94	115	42	35	49	40
Tspan11	ENSMUSG00000030351	844.6849803	-1.411743131	0.315773244	-4.470749682	7.79459E-06	0.009179279	1402	1757	892	862	353	447	406	639
Hist1h2ad	ENSMUSG00000071478	43.66655185	-1.605926072	0.407224753	-3.943586587	8.0272E-05	0.035360644	40	107	41	75	24	19	23	20
Lsr	ENSMUSG00000001247	403.2309522	-1.696471991	0.268392687	-6.320857737	2.60115E-10	2.4443E-06	890	633	502	441	206	209	181	165
Rimklb	ENSMUSG00000040649	60.58136567	-1.829022286	0.447456305	-4.087599762	4.35859E-05	0.025657524	139	64	109	66	41	12	34	20
Ly6d	ENSMUSG00000034634	5414.062031	-2.352429977	0.553969072	-4.246500561	2.17135E-05	0.016353624	3055	13550	12918	6696	1161	1244	3737	951
4930481A15Rik	ENSMUSG00000086938	150.0490934	-2.377103166	0.58829502	-4.040665117	5.32998E-05	0.027073429	89	618	163	136	37	31	58	68
Gulo	ENSMUSG00000034450	45.19409874	-2.455849138	0.482481479	-5.090038159	3.57991E-07	0.000961156	76	121	74	35	18	21	10	8
Guca1a	ENSMUSG00000023982	124.8593841	-2.493240255	0.678372319	-3.675327227	0.000237545	0.081171201	49	583	78	138	36	27	49	39
Ly6k	ENSMUSG00000044678	438.3079278	-2.58237902	0.451272697	-5.722435766	1.05008E-08	3.94702E-05	976	294	551	1182	175	71	121	135
Mapk15	ENSMUSG00000063704	10.48941833	-2.59584537	0.659481693	-3.936190188	8.27854E-05	0.035360644	13	28	11	20	5	3	3	1
Gm26917	ENSMUSG00000097971	3932.585527	-2.786087543	0.686288254	-4.059646261	4.91471E-05	0.025657524	2252	18573	1140	5512	1056	804	811	1312
Ptprv	ENSMUSG00000097993	13.11079293	-4.36468208	1.211114255	-3.603856582	0.00031353	0.094437519	60	19	21	0	0	1	2	2

Table S4. Genes differentially expressed ABC_Hoxb9 Male vs ABC Male

								Read count					
Gene	ENSEMBL_Gene_ID	baseMean	log2FoldChange	lfcSE	stat	pvalue	padj	166836_Normal	166838_Normal	182098_Turq19	169229_Normal	182083_Turq19	182086_Turq19
Nr6a1	ENSMUSG00000063972	811.1701312	3.641534655	0.245763457	14.81723402	1.13357E-49	2.06933E-45	162	107	92	1392	1573	1541
Dnah10	ENSMUSG00000038011	152.0821039	6.82144082	0.624115351	10.92977573	8.30534E-28	7.5807E-24	1	0	7	345	354	205
Rbm24	ENSMUSG00000038132	5273.455917	1.773547782	0.167771116	10.57123433	4.05127E-26	2.4652E-22	2269	2637	2253	9027	8255	7198
Nr5a1	ENSMUSG00000026751	27438.90468	1.928280233	0.192796792	10.00161993	1.49924E-23	6.84217E-20	9584	11915	12757	48019	37251	45108
Mgst2	ENSMUSG00000074604	105.3306037	4.490598127	0.513797543	8.740014792	2.3308E-18	8.50975E-15	8	12	7	322	102	181
Fos	ENSMUSG00000021250	384.6775037	3.007200438	0.350950691	8.568726353	1.04636E-17	3.18355E-14	86	87	83	482	495	1075
Paqr5	ENSMUSG00000032278	185.3234288	-2.309866975	0.270957864	-8.524819832	1.53048E-17	3.99127E-14	269	340	317	58	47	82
Trib3	ENSMUSG00000032715	170.4274959	-3.619400083	0.430849623	-8.400610998	4.44161E-17	1.01352E-13	379	424	143	22	27	28
Frzb	ENSMUSG00000027004	836.0153116	2.038833721	0.251602439	8.103394107	5.34467E-16	1.08408E-12	440	277	265	1445	1175	1415
Agtr1b	ENSMUSG00000054988	1536.290434	1.49896675	0.191315668	7.835044379	4.68677E-15	8.5557E-12	814	904	691	2611	2128	2070
Kcnj2	ENSMUSG00000041695	487.6945518	1.447861269	0.190014778	7.619729798	2.54207E-14	4.21869E-11	239	279	267	692	629	820
Fam19a4	ENSMUSG00000046500	150.052925	2.16280513	0.293579078	7.367027452	1.74475E-13	2.65311E-10	43	75	46	280	205	251
Cfap69	ENSMUSG00000040473	339.1089167	1.286672976	0.174905264	7.356399375	1.88937E-13	2.65311E-10	196	197	199	455	454	533
Atp1b2	ENSMUSG00000041329	4032.080709	-1.153090881	0.157907027	-7.302340516	2.82804E-13	3.68757E-10	5416	5324	5949	2329	2354	2822
Fosb	ENSMUSG00000003545	159.7474241	2.972023973	0.413977847	7.179186021	7.01277E-13	8.15558E-10	35	58	15	335	278	237
Sbspon	ENSMUSG00000032719	1233.201099	-1.631661149	0.227359428	-7.176571316	7.14814E-13	8.15558E-10	1867	1503	2224	690	619	496
Mthfd1l	ENSMUSG00000040675	394.8709454	1.756469142	0.246346008	7.130089728	1.00304E-12	1.07708E-09	137	230	174	692	552	584
Pgm5	ENSMUSG00000041731	2289.168586	-2.038119805	0.29106696	-7.002236892	2.51908E-12	2.55476E-09	4090	4694	2262	832	839	1018
Dusp5	ENSMUSG00000034765	147.9975236	1.437935789	0.208199793	6.906518816	4.96691E-12	4.77216E-09	75	86	79	229	207	213
Steap1	ENSMUSG00000015652	4208.069022	1.483700474	0.221663401	6.69348422	2.17919E-11	1.98905E-08	1996	2781	1872	6132	5417	7049
Hpcal4	ENSMUSG00000046093	334.9135465	2.224448287	0.333777622	6.664462024	2.65637E-11	2.30914E-08	126	161	68	565	653	437
Wnt2	ENSMUSG00000010797	73.84562318	-3.588946637	0.546114877	-6.571779658	4.97174E-11	4.03224E-08	179	169	62	20	7	8
Fam169a	ENSMUSG00000041817	25.71259866	3.854572296	0.586821405	6.568562307	5.08034E-11	4.03224E-08	4	3	3	46	58	41
Klhdc7a	ENSMUSG00000078234	691.3638177	-2.274411209	0.347327557	-6.548317766	5.81888E-11	4.42599E-08	1369	1374	695	267	290	153
Slit1	ENSMUSG00000025020	676.9815123	-1.647007297	0.253274036	-6.502866714	7.88036E-11	5.75424E-08	1207	1047	825	421	278	284
Egr1	ENSMUSG00000038418	1195.432914	1.60613355	0.251572311	6.384381275	1.72092E-10	1.20828E-07	590	555	629	1535	1426	2438
Cpb1	ENSMUSG00000011463	1154.039819	1.114970515	0.175539376	6.351683269	2.12971E-10	1.43992E-07	816	727	643	1749	1516	1473
1810041L15Rik	ENSMUSG00000062760	73.96064017	-3.352228182	0.528672156	-6.340844972	2.28508E-10	1.48979E-07	245	85	75	13	14	13
Cela1	ENSMUSG00000023031	300.3591209	1.320252192	0.208989162	6.317323724	2.66132E-10	1.67525E-07	172	141	203	397	447	443
Dkk2	ENSMUSG00000028031	269.3411136	-1.206639835	0.191678566	-6.295121349	3.07159E-10	1.86907E-07	363	362	403	145	159	184
Lin7a	ENSMUSG00000019906	1025.814724	1.803173034	0.287815956	6.26502108	3.72775E-10	2.19516E-07	488	488	395	2146	1596	1042
Nt5e	ENSMUSG00000032420	80.81971649	-1.929206081	0.308747557	-6.248490183	4.14439E-10	2.36425E-07	159	115	110	32	41	28
Tspan7	ENSMUSG00000058254	1035.205687	-1.107060862	0.178791325	-6.191915966	5.94372E-10	3.28796E-07	1448	1259	1536	649	583	738
Plin1	ENSMUSG00000030546	1388.832783	-2.272761886	0.373505401	-6.08495053	1.16527E-09	6.25649E-07	3482	2143	1279	343	589	497
Cspg5	ENSMUSG00000032482	1383.626308	1.15906072	0.192840094	6.010475815	1.8498E-09	9.64801E-07	896	792	879	1540	2111	2083
Mid1	ENSMUSG00000035299	799.443562	1.097425918	0.184206721	5.957578061	2.56003E-09	1.29815E-06	446	567	515	1228	1001	1040
Tnfaip8	ENSMUSG00000062210	376.9331839	1.50346739	0.254791142	5.900783586	3.61779E-09	1.78494E-06	165	209	216	717	442	513
Exoc3l4	ENSMUSG00000021280	154.2430421	-1.267063492	0.217953685	-5.813452934	6.11972E-09	2.93988E-06	224	240	190	85	90	96
Gdf15	ENSMUSG00000038508	69.08074119	-2.443812257	0.420806397	-5.807450355	6.34314E-09	2.96908E-06	129	159	63	23	23	18
Slc29a4	ENSMUSG00000050822	198.1680778	-1.206592712	0.208074283	-5.798855554	6.6769E-09	3.04717E-06	291	239	299	109	125	125
Cox6a2	ENSMUSG00000030785	38.77786332	-3.341112475	0.576854298	-5.791952121	6.9573E-09	3.09769E-06	69	107	35	10	7	4
Penk	ENSMUSG00000045573	135.0352103	-1.507702023	0.261427113	-5.767198391	8.06002E-09	3.50323E-06	229	151	220	72	66	72
Map6d1	ENSMUSG00000041205	49.82055941	1.967603981	0.342124283	5.751138049	8.86447E-09	3.76328E-06	17	23	21	89	85	64
Ama5	ENSMUSG00000027712	9137.620151	-1.243399416	0.216753164	-5.736476423	9.66665E-09	4.01056E-06	12423	11209	14913	4424	6547	5309
Kcnk2	ENSMUSG00000037624	264.8281644	-1.465054446	0.255653381	-5.730628084	1.00059E-08	4.05908E-06	418	422	327	179	108	136
Fstl4	ENSMUSG00000036264	24.65736113	2.949956187	0.519652097	5.676790691	1.37245E-08	5.44655E-06	6	5	6	34	56	41
Cpa3	ENSMUSG00000001865	57.95538103	1.815944878	0.32448714	5.596353913	2.18907E-08	8.50243E-06	27	18	32	87	86	97
Chl1	ENSMUSG00000030077	281.3478027	1.206816812	0.216101554	5.584489287	2.34388E-08	8.91407E-06	190	186	134	379	371	428
Prkg2	ENSMUSG00000029334	30.74707979	-2.563170366	0.460640122	-5.564366285	2.63107E-08	9.80207E-06	66	39	52	8	12	7
Ap1s2	ENSMUSG00000031367	695.5804607	0.92856082	0.168724199	5.50342407	3.72485E-08	1.35994E-05	463	445	529	967	824	945
Ly6d	ENSMUSG00000034634	5887.589686	-1.644395494	0.298992026	-5.499797152	3.80228E-08	1.36099E-05	8995	9206	8563	2126	2159	4276
Bcam	ENSMUSG00000002980	3901.500115	-0.651860589	0.119143108	-5.47124045	4.46896E-08	1.56886E-05	4690	4842	4772	3093	3009	3002
Ckap2	ENSMUSG00000037725	159.104734	1.431784668	0.262800805	5.448174584	5.08894E-08	1.7528E-05	98	92	69	225	191	281
Apobec3	ENSMUSG00000009585	277.3259051	0.969816691	0.178474497	5.433923083	5.51284E-08	1.86364E-05	196	193	173	346	395	361
Ncam1	ENSMUSG00000039542	236.6945035	-1.167093931	0.217964939	-5.354503045	8.57919E-08	2.84751E-05	320	283	380	137	135	166
Col5a1	ENSMUSG00000026837	565.3864131	-1.243681773	0.232980216	-5.33814328	9.39032E-08	3.06108E-05	951	830	604	337	373	297
Rftn2	ENSMUSG00000025978	1918.492163	-0.809012404	0.152260883	-5.313330561	1.0764E-07	3.4473E-05	2683	2179	2466	1405	1413	1365
P4ha3	ENSMUSG00000051048	202.6466646	1.545987005	0.294881699	5.242736368	1.58213E-07	4.9796E-05	83	120	107	406	231	269
Atf5	ENSMUSG00000038539	1406.063858	-1.33372337	0.259934149	-5.13100481	2.882E-07	8.91709E-05	2004	2649	1386	765	828	803
Pid1	ENSMUSG00000045658	1367.769562	-1.05164338	0.205231538	-5.124180173	2.98835E-07	9.09206E-05	2179	1532	1825	976	921	773
Htra3	ENSMUSG00000029096	189.7669777	-1.337749516	0.261306198	-5.119471051	3.06394E-07	9.16921E-05	303	258	255	78	136	109
Chgb	ENSMUSG00000027350	8095.581355	-1.188705675	0.234087049	-5.078049722	3.81329E-07	0.000112277	14303	8586	10873	4706	4533	5573
Steap3	ENSMUSG00000026389	310.1783864	1.015680981	0.200891592	5.055866053	4.28442E-07	0.000120286	203	239	173	428	432	385
Hdc	ENSMUSG00000027360	110.7635733	-2.112889946	0.41815351	-5.05290496	4.3514E-07	0.000120286	271	134	135	26	60	39
Sgk2	ENSMUSG00000050711	2584.996508	-1.286987469	0.254717831	-5.052600602	4.35835E-07	0.000120286	4700	2642	3659	1359	1404	1745
Per3	ENSMUSG00000028957	572.1838259	1.434590944	0.283999042	5.051393596	4.38598E-07	0.000120286	226	420	281	939	905	663
Dlk1	ENSMUSG00000040856	1526.188676	-1.419475048	0.281175867	-5.048353056	4.45635E-07	0.000120286	2848	1601	2217	669	771	1052
Npas2	ENSMUSG00000026077	128.286461	-3.333086666	0.660728031	-5.044566764	4.5455E-07	0.000120286	492	131	78	11	20	38
Tubb2a	ENSMUSG00000058672	1237.174186	1.034832624	0.205139867	5.044522251	4.54656E-07	0.000120286	747	904	783	1760	1326	1902
Per2	ENSMUSG00000055866	1319.724987	1.724530894	0.342387983	5.03677401	4.73443E-07	0.000123467	383	933	523	2512	1721	1847
Junb	ENSMUSG00000052837	1537.064516	1.639579174	0.327288633	5.009581782	5.45484E-07	0.000140251	463	674	1104	2305	1938	2739
3110002H16Rik	ENSMUSG00000024410	851.2247873	0.730811755	0.147118391	4.967507802	6.78188E-07	0.000171949	649	684	587	1065	1072	1050
Rimklb	ENSMUSG00000040649	25.55652204	-3.569394691	0.721252118	-4.948886253	7.46393E-07	0.000186649	40	18	84	5	3	4
Syp	ENSMUSG00000031144	299.0434991	-0.99936094	0.202559665	-4.933662083	8.0702E-07	0.000199083	448	339	409	199	182	218
Agmat	ENSMUSG00000040706	109.9081043	1.749480608	0.356346257	4.909496239	9.13107E-07	0.00022225	43	76	32	141	165	203
Fah	ENSMUSG00000030630	277.5693319	-2.329499925	0.474897669	-4.905267126	9.33003E-07	0.000224105	592	535	262	143	97	37
Gm10259	ENSMUSG00000069083	134.3520769	1.049901905	0.214274679	4.899794548	9.59369E-07	0.000227445	87	80	96	190	169	184
Egfl6	ENSMUSG00000000402	1006.992389	1.61791971	0.330863982	4.889984403	1.00844E-06	0.000236014	520	695	270	1668	1532	1357
Prss35	ENSMUSG00000033491	9930.443383	-1.041148054	0.213173269	-4.884046011	1.03931E-06	0.000240159	13228	14921	11949	8033	5346	6106
Nr4a2	ENSMUSG00000026826	184.9261161	1.323367864	0.272155921	4.86253564	1.15892E-06	0.00026445	87	138	92	306	218	268
Nupr1	ENSMUSG00000030717	152.3822309	-1.195960888	0.246092968	-4.859793018	1.17509E-06	0.000264829	185	213	239	72	104	101
Slc18a1	ENSMUSG00000036330	140.5386292	-1.255212075	0.258760383	-4.8508665	1.22923E-06	0.000273654	233	150	212	78	83	89
Rab3c	ENSMUSG00000021700	223.3071547	-1.565523907	0.32367495	-4.836716299	1.32002E-06	0.000290324	489	278	235	113	127	98
Pla2g2d	ENSMUSG00000041202	249.0889622	1.485023824	0.307456326	4.830031772	1.36511E-06	0.00029464	103	123	167	274	333	494
Mthfd2	ENSMUSG00000005667	619.374188	-1.413069033	0.292618966	-4.829041164	1.37192E-06	0.00029464	923	1192	587	355	283	377
Parvb	ENSMUSG00000022438	907.9631483	-1.232852212	0.256581684	-4.804911216	1.5482E-06	0.000328633	1694	1150	978	468	558	600
Cdk1	ENSMUSG00000019942	147.4672881	1.160514945	0.242896962	4.777807583	1.77217E-06	0.00037185	94	79	101	176	192	243
Gucy1b3	ENSMUSG00000028005	306.6615008	-0.98913099	0.207202732	-4.773735262	1.8084E-06	0.00037514	357	406	461	206	232	178
Knstrn	ENSMUSG00000027331	103.0268642	1.417207893	0.298321675	4.750603158	2.02811E-06	0.00041599	71	60	37	148	135	167
Cda	ENSMUSG00000028755	146.8801099	-1.646082627	0.347579709	-4.735842126	2.18147E-06	0.000442476	203	157	309	51	71	92
Mki67	ENSMUSG00000031004	291.4866668	1.137088925	0.240383525	4.730311389	2.24176E-06	0.000449706	236	163	148	397	370	435
Car2	ENSMUSG00000027562	1253.218039	1.10429218	0.234213154	4.714902473	2.41827E-06	0.000479842	680	752	955	1387	1652	2094
Lonrf1	ENSMUSG00000039633	331.2282891	1.233435024	0.262628882	4.696494213	2.64665E-06	0.000519511	179	237	177	593	445	357
Mpped2	ENSMUSG00000016386	3139.876544	0.63849334	0.136304467	4.684317059	2.80895E-06	0.000539869	2318	2538	2513	3699	3697	4075
Cd83	ENSMUSG00000015396	416.4796849	-1.161294329	0.247913281	-4.684276398	2.80951E-06	0.000539869	729	566	432	281	259	231
Nr5a1os	ENSMUSG00000087164	27.26914142	2.404874689	0.513904957	4.679609827	2.87421E-06	0.00054655	9	7	10	64	27	46
Nr4a1	ENSMUSG00000023034	3218.217325	1.748964944	0.374141382	4.67460973	2.94513E-06	0.000554262	861	2284	1282	6701	4141	4040
Aldh1a3	ENSMUSG00000015134	467.2695007	-1.107896714	0.237124674	-4.672211853	2.97973E-06	0.000555051	700	725	489	350	268	271
Gm5532	ENSMUSG00000073535	488.5305659	-0.84929822	0.182738729	-4.647609322	3.35804E-06	0.000619202	607	581	698	392	338	316
Zfand5	ENSMUSG00000024750	8665.759624	0.941811005	0.203888944	4.619235286	3.85157E-06	0.000703104	5182	6080	6539	12992	9122	12080
Tfap2b	ENSMUSG00000025927	55.44423196	-1.934508352	0.419775028	-4.608440769	4.057E-06	0.000733272	128	54	82	18	27	24
Olfml3	ENSMUSG00000027848	969.5445239	-0.888547516	0.193186402	-4.599430952	4.23647E-06	0.000751378	1081	1447	1249	603	679	758
Th	ENSMUSG00000000214	664.8652159	-1.271184803	0.276387647	-4.599282266	4.23949E-06	0.000751378	1213	944	663	343	352	474
Aldh1l2	ENSMUSG00000020256	61.43684502	-3.40199281	0.74213638	-4.584053422	4.56048E-06	0.000797931	167	156	14	14	10	8
Atp1a3	ENSMUSG00000040907	839.1563675	0.979117024	0.213823698	4.579085644	4.67013E-06	0.000797931	605	659	431	1193	1083	1065
Zdhhc14	ENSMUSG00000034265	239.2364174	0.950774351	0.207657838	4.578562317	4.68183E-06	0.000797931	151	185	153	289	360	297
Bhlha15	ENSMUSG00000052271	68.00211665	-2.708629647	0.591612861	-4.578381956	4.68586E-06	0.000797931	134	174	45	31	16	8
Col6a5	ENSMUSG00000091345	170.3833496	-1.259524317	0.275195686	-4.57683162	4.72071E-06	0.000797931	289	180	252	90	122	89
Bcl11b	ENSMUSG00000048251	70.78187861	1.415546566	0.309417976	4.574868549	4.76519E-06	0.00079806	30	38	47	106	119	84
Kif22	ENSMUSG00000030677	82.12110479	1.282323201	0.280817175	4.566398765	4.96174E-06	0.000823424	59	46	38	101	123	124
RP23-177G20.1	ENSMUSG00000107863	11.29025076	5.962062886	1.308885947	4.555066774	5.23689E-06	0.000853948	0	1	0	14	28	24
Cdh12	ENSMUSG00000040452	69.66354156	3.090593993	0.678509851	4.554972911	5.23923E-06	0.000853948	12	16	16	237	23	114
Ikzf4	ENSMUSG00000002578	328.2742075	-1.518673221	0.33362031	-4.552100622	5.31129E-06	0.000858032	728	413	319	190	145	175
Hlf	ENSMUSG00000003949	2227.842391	1.41579152	0.31178142	4.540974636	5.59948E-06	0.000896653	792	1475	1376	4308	2947	2468
Cxcl12	ENSMUSG00000061353	776.7368749	-1.124499586	0.247850689	-4.537004069	5.7059E-06	0.00090575	764	1062	1368	468	488	509
Smpdl3b	ENSMUSG00000028885	403.4786527	1.424143899	0.315357333	4.515968877	6.30279E-06	0.000991875	129	218	311	571	625	567
Rrm2	ENSMUSG00000020649	200.7001551	1.259444837	0.279074736	4.512930312	6.3938E-06	0.000997597	156	120	79	281	274	294
Itgb8	ENSMUSG00000025321	411.931421	-1.136509041	0.253143141	-4.489590504	7.13602E-06	0.001103967	667	580	452	289	289	195
Six4	ENSMUSG00000034460	85.3605262	1.116569134	0.248867753	4.48659628	7.237E-06	0.00111018	55	48	58	107	120	123
Hid1	ENSMUSG00000034586	640.697366	-0.897692436	0.20170544	-4.450511773	8.56659E-06	0.001301371	781	1012	708	471	446	426
Kcnd3	ENSMUSG00000040896	352.5475759	-1.163155586	0.261440278	-4.449029794	8.62591E-06	0.001301371	578	543	342	190	219	243
Ccne1	ENSMUSG00000002068	51.40427609	1.376878286	0.311138328	4.425293067	9.63114E-06	0.001429637	31	32	23	78	66	79
Glipr2	ENSMUSG00000028480	100.6326047	-1.824346566	0.412257704	-4.425257672	9.63272E-06	0.001429637	253	116	102	34	50	48
Frmd5	ENSMUSG00000027238	308.5170237	1.254043188	0.283906998	4.417091505	1.00038E-05	0.001472735	165	234	148	567	362	375
Ccnb2	ENSMUSG00000032218	93.646426	1.271830893	0.290361728	4.380160234	1.18592E-05	0.001731919	52	56	56	116	109	173
Gm43549	ENSMUSG00000106334	218.6876104	1.650182437	0.377453149	4.371886786	1.23177E-05	0.001784606	81	78	158	320	453	222
Pea15a	ENSMUSG00000013698	1770.064806	-0.705215423	0.161790252	-4.358825167	1.30763E-05	0.001879583	2469	2189	1925	1331	1377	1330
Avpr1a	ENSMUSG00000020123	80.03141989	-2.24577525	0.519823701	-4.356814136	1.31969E-05	0.001882108	145	120	132	56	14	14
Plin5	ENSMUSG00000011305	45.53551057	-1.837287844	0.422187614	-4.351827912	1.35007E-05	0.001910509	95	69	49	28	18	14
Lcn2	ENSMUSG00000026822	19.07541613	-2.739861335	0.630756443	-4.343770669	1.40058E-05	0.001953918	36	44	19	7	2	6
Gata1	ENSMUSG00000031162	31.1883529	-1.807619632	0.416164341	-4.343523586	1.40215E-05	0.001953918	56	51	38	14	17	10
Hoxc8	ENSMUSG00000001657	762.8319993	-0.924543214	0.213445098	-4.33152704	1.48079E-05	0.002047862	871	873	1253	491	549	540
Ciapin1	ENSMUSG00000031781	955.7178131	0.608711614	0.140602789	4.329299712	1.49584E-05	0.002053128	732	737	802	1118	1145	1201
Rbms3	ENSMUSG00000039607	788.4523645	-0.795127202	0.184499007	-4.309655719	1.63509E-05	0.002227504	878	941	1182	568	602	559
Insm1	ENSMUSG00000068154	66.30212499	-1.884121868	0.439332449	-4.28860166	1.79802E-05	0.002431316	143	52	118	24	24	37
Pak3	ENSMUSG00000031284	2208.544469	-0.771156726	0.179974439	-4.284812489	1.82893E-05	0.002454113	2872	3038	2446	1682	1414	1800
Syt1	ENSMUSG00000035864	110.9936341	-1.347834733	0.314675094	-4.283258377	1.84176E-05	0.002454113	224	121	133	61	63	64
Slc18a2	ENSMUSG00000025094	108.3077177	-1.337595663	0.312449386	-4.280999492	1.86056E-05	0.002461194	183	113	169	67	45	72
Nrxn1	ENSMUSG00000024109	142.5119076	-1.677777904	0.392106942	-4.278878351	1.87837E-05	0.002466887	235	115	301	49	79	76
Hs3st1	ENSMUSG00000051022	533.6212262	0.808736786	0.189319747	4.271803656	1.93898E-05	0.002512257	447	353	364	753	614	671
Chga	ENSMUSG00000021194	6300.082438	-1.303450585	0.305140868	-4.271635571	1.94045E-05	0.002512257	9487	5817	11598	3035	3157	4707
Sparcl1	ENSMUSG00000029309	1612.766187	-0.862006038	0.20266049	-4.253448898	2.10503E-05	0.002706149	2306	2206	1730	1194	1276	964
Zfp185	ENSMUSG00000031351	209.2415304	-1.112940497	0.261808366	-4.250973771	2.12843E-05	0.002717098	352	286	221	153	134	110

Table S4. Genes differentially expressed ABC_Hoxb9 Male vs ABC Male

								Read count					
Gene	ENSEMBL_Gene_ID	baseMean	log2FoldChange	lfcSE	stat	pvalue	padj	166836_Normal	166838_Normal	182098_Turq19	169229_Normal	182083_Turq19	182086_Turq19
Tubb2b	ENSMUSG00000045136	418.5396414	1.023461693	0.241586642	4.236416726	2.27115E-05	0.002879157	232	338	258	555	460	668
Srebf1	ENSMUSG00000020538	3228.829072	-1.202333941	0.286028738	-4.203542452	2.6277E-05	0.003308182	5151	5488	2865	2329	1857	1684
Lsr	ENSMUSG00000001247	283.1756427	-1.108270339	0.263881828	-4.199873661	2.67064E-05	0.003339214	426	297	439	136	204	199
Klrg2	ENSMUSG00000071537	245.896566	-0.978859296	0.234352281	-4.176871208	2.95546E-05	0.003670202	316	280	383	164	195	138
Pcsk1n	ENSMUSG00000039278	383.7963342	-0.968025839	0.232271051	-4.167656005	3.07748E-05	0.003795904	524	426	575	207	263	309
Hoxc6	ENSMUSG00000001661	779.2579327	-0.721850596	0.173300011	-4.165323424	3.10911E-05	0.003809187	929	959	1022	617	646	502
Faim2	ENSMUSG00000023011	186.8510305	-1.320852704	0.317539162	-4.15965292	3.18732E-05	0.003878963	382	193	226	90	121	109
Zswim6	ENSMUSG00000032846	355.830239	0.763581377	0.183646231	4.157893004	3.21196E-05	0.003883073	298	239	254	483	444	417
Ociad2	ENSMUSG00000029153	600.0175298	0.820427614	0.197690863	4.150053279	3.32398E-05	0.003992055	473	480	349	807	784	708
Plk1	ENSMUSG00000030867	75.24432344	1.372584478	0.331057263	4.146063629	3.3824E-05	0.004035666	43	34	49	84	98	144
Chac1	ENSMUSG00000027313	77.72657713	-1.377953876	0.332627834	-4.142629492	3.43346E-05	0.004065097	130	124	83	42	55	32
Racgap1	ENSMUSG00000023015	157.7841987	1.059080788	0.255728818	4.141421363	3.4516E-05	0.004065097	119	104	84	182	199	258
Cdca2	ENSMUSG00000048922	42.83410558	1.600410171	0.387753942	4.127385946	3.6691E-05	0.004266612	26	25	13	57	56	80
Lnx1	ENSMUSG00000029228	152.7537176	-1.38522923	0.335620789	-4.127364205	3.66945E-05	0.004266612	310	167	187	75	111	68
Actg1	ENSMUSG00000062825	18153.77224	0.6294144448	0.152608351	4.124377477	3.71739E-05	0.004294995	12984	15636	14146	23458	22312	20387
Traf4	ENSMUSG00000017386	309.3234089	0.794445591	0.19400769	4.094918047	4.22318E-05	0.004848686	240	250	189	383	381	414
Neto2	ENSMUSG00000036902	125.3040039	0.882450042	0.216704236	4.072140246	4.65831E-05	0.005290626	82	88	95	160	151	176
Slc4a11	ENSMUSG00000074796	92.41990767	-1.555342622	0.381983559	-4.071752786	4.66607E-05	0.005290626	107	171	135	69	45	27
Suox	ENSMUSG00000049858	659.1906862	-0.712076157	0.175500642	-4.057399148	4.96222E-05	0.005557291	897	804	754	561	453	485
Lrat	ENSMUSG00000028003	19.52735844	-2.770329728	0.682811146	-4.057241512	4.96557E-05	0.005557291	30	21	51	8	6	1
Dhh	ENSMUSG00000023000	432.0742937	-0.9257802	0.228251019	-4.055974004	4.99258E-05	0.005557291	665	599	434	327	299	268
Cpeb1	ENSMUSG00000025986	520.8678764	0.967181581	0.238678455	4.052236645	5.07303E-05	0.005612617	351	354	353	867	692	508
Pdgfd	ENSMUSG00000032006	1118.682583	-0.760131781	0.187830037	-4.046906342	5.1899E-05	0.005707326	1503	1494	1223	956	796	740
Etv4	ENSMUSG00000017724	122.4242107	0.942811535	0.233150257	4.043793675	5.25932E-05	0.005749036	93	84	75	168	176	140
Tspan11	ENSMUSG00000030351	977.1611964	-1.118791587	0.277710736	-4.028622026	5.61047E-05	0.005985548	1507	1037	1471	421	680	747
Gm21857	ENSMUSG00000095134	106.8579732	1.002202919	0.248839525	4.027506967	5.63714E-05	0.005985548	79	72	63	164	142	122
Ccna2	ENSMUSG00000027715	143.9642223	1.058331069	0.262783311	4.027390722	5.63993E-05	0.005985548	122	86	73	180	195	208
Sord	ENSMUSG00000027227	5442.175027	0.852213272	0.211733615	4.024931377	5.69919E-05	0.005985548	3046	3995	4600	7689	7122	6202
Ptprn	ENSMUSG00000026204	209.2467642	-1.045424662	0.259820211	-4.023646419	5.73039E-05	0.005985548	361	258	227	123	158	128
Camkv	ENSMUSG00000032936	1616.278286	0.815798385	0.202770784	4.023254089	5.73995E-05	0.005985548	1032	1197	1285	2295	2234	1655
6430573F11Rik	ENSMUSG00000039620	174.2666171	0.970284357	0.241196295	4.022799585	5.75104E-05	0.005985548	117	112	124	256	260	176
Slc31a2	ENSMUSG00000066152	650.4244709	-0.631960782	0.157105468	-4.022525701	5.75774E-05	0.005985548	739	789	843	551	485	495
Dbh	ENSMUSG00000000889	1248.142421	-0.997680203	0.248056191	-4.021992748	5.77078E-05	0.005985548	1974	1317	1699	689	761	1048
Ddc	ENSMUSG00000020182	202.2755968	-1.262810715	0.314300967	-4.017839108	5.87343E-05	0.006057594	278	204	375	86	127	144
Myot	ENSMUSG00000024471	56.33413627	1.489116893	0.370820691	4.015733013	5.92613E-05	0.006077614	31	34	24	113	80	57
D930016D06Rik	ENSMUSG00000097392	107.0065075	1.209674013	0.30280176	3.994937195	6.47114E-05	0.006599479	65	47	82	119	179	150
Wisp2	ENSMUSG00000027656	27.63785264	2.186899876	0.547867235	3.991660268	6.56123E-05	0.006654183	8	13	9	24	41	71
Sun2	ENSMUSG00000042524	1959.570288	-0.54766398	0.137304051	-3.988694994	6.64378E-05	0.006688509	2366	2443	2172	1631	1623	1523
Fam64a	ENSMUSG00000020808	61.2169183	1.503846138	0.377227773	3.986573224	6.70344E-05	0.006688509	44	26	26	70	79	122
Nr1h4	ENSMUSG00000047638	188.2740738	0.938459797	0.235426862	3.986205269	6.71384E-05	0.006688509	135	120	132	303	203	236
Rnd3	ENSMUSG00000017144	562.0778444	0.801450268	0.201188466	3.983579601	6.78849E-05	0.006688509	332	467	431	783	688	671
Kcnn2	ENSMUSG00000054477	4631.750722	0.731446193	0.183645116	3.982932997	6.807E-05	0.006688509	2874	3521	4052	5619	5643	6081
Slc35f2	ENSMUSG00000042195	37.32932634	-1.539792276	0.386624401	-3.982656736	6.81492E-05	0.006688509	72	45	49	18	23	16
Igf2bp2	ENSMUSG00000033581	282.0886669	0.698542651	0.175517186	3.979910265	6.89413E-05	0.006730071	236	214	196	347	346	355
Rgs7bp	ENSMUSG00000021719	377.4442783	0.813921858	0.204803114	3.974167392	7.06259E-05	0.006857848	290	269	262	560	490	393
Pclo	ENSMUSG00000061601	122.3815225	-1.29127948	0.325665306	-3.965050798	7.33803E-05	0.007087607	237	171	113	78	63	72
Fam181b	ENSMUSG00000051515	335.0259434	-0.63348465	0.159833141	-3.963412386	7.3886E-05	0.007098888	399	405	418	273	251	263
Tpx2	ENSMUSG00000027469	144.7638874	0.96516283	0.243618182	3.961784883	7.43916E-05	0.007110041	114	96	85	190	162	222
Camk2b	ENSMUSG00000057897	400.1141363	-1.045379948	0.265207226	-3.941747607	8.08901E-05	0.007690876	543	667	407	255	212	317
Ndufa4l2	ENSMUSG00000040280	240.4354708	-0.906539219	0.230904626	-3.926033155	8.63583E-05	0.008168238	302	269	370	154	198	149
Malt1	ENSMUSG00000032688	919.7664413	0.768525872	0.196926278	3.902607008	9.51621E-05	0.008954562	609	837	596	1159	1136	1182
E130012A19Rik	ENSMUSG00000043439	118.9157044	0.868885815	0.222834806	3.899237431	9.64961E-05	0.009033521	73	86	94	155	156	149
Speg	ENSMUSG00000026207	248.1606696	-0.682933129	0.175361854	-3.894422384	9.8433E-05	0.009167829	301	321	295	186	203	182
Rev1	ENSMUSG00000026082	634.3872379	0.729592607	0.187476324	3.891651974	9.9564E-05	0.009226095	468	517	447	919	765	690
Mybpc1	ENSMUSG00000020061	9394.481961	1.084364553	0.27901008	3.886470891	0.000101712	0.009377549	6500	7452	4112	15594	12560	10150
Cyp2d22	ENSMUSG00000061740	1029.869144	-0.630955951	0.162397896	-3.88524709	0.000102226	0.009377554	1183	1197	1375	757	780	888
Aurkb	ENSMUSG00000020897	64.63770226	1.323940721	0.342034039	3.870786441	0.000108485	0.00990195	45	32	34	65	95	117
Thra	ENSMUSG00000058756	2150.000944	-0.74266064	0.19201283	-3.867765715	0.000109837	0.009975505	3109	2176	2790	1501	1656	1669
Rtn4rl1	ENSMUSG00000045287	5413.863964	-0.564513715	0.146156038	-3.862404332	0.000112277	0.010146575	6419	6089	6871	4524	4610	3970
Cryba4	ENSMUSG00000066975	89.19847543	-1.882307362	0.489042365	-3.848965848	0.000118618	0.010666814	182	171	69	48	17	49
Tmem108	ENSMUSG00000042757	56.26423378	1.232661358	0.321211972	3.83753242	0.000124277	0.011120949	44	27	30	69	86	81
Npas4	ENSMUSG00000045903	10.18372232	5.811161757	1.515350517	3.834863083	0.000125634	0.011187578	0	1	0	2	27	31
Gm2a	ENSMUSG00000000594	7057.545762	-0.497122103	0.129678361	-3.833500823	0.000126332	0.011195134	8438	8253	8093	5572	5730	6259
Cited1	ENSMUSG00000051159	224.5901391	-1.469335284	0.384699298	-3.819438432	0.000133756	0.011795714	225	304	462	84	93	180
Ptk	ENSMUSG00000022033	113.9715047	1.29020434	0.33861256	3.810267228	0.000138817	0.012131878	90	57	51	131	136	218
Dusp26	ENSMUSG00000039661	42.60715353	-1.380618706	0.362355282	-3.810124418	0.000138897	0.012131878	59	50	76	18	25	28
Slc6a5	ENSMUSG00000039728	102.310154	2.03876788	0.535304152	3.808615856	0.000139747	0.012147995	44	66	10	201	118	175
Rasgrf1	ENSMUSG00000032356	318.0220267	1.267824371	0.334221823	3.79336202	0.000148621	0.012858202	116	157	287	435	462	451
Inhbb	ENSMUSG00000037035	137.7303923	0.831119833	0.219212528	3.791388389	0.000149807	0.012861515	84	101	113	174	178	177
Filip1l	ENSMUSG00000043336	902.4789056	-0.648486979	0.171061601	-3.790955867	0.000150069	0.012861515	1195	1116	995	700	633	776
Coq10b	ENSMUSG00000025981	2232.794745	1.356827515	0.358302215	3.786824249	0.000152585	0.01296079	623	1374	1765	3295	2700	3640
Kpna2	ENSMUSG00000018362	450.8287847	0.865142036	0.228467193	3.786723267	0.000152647	0.01296079	276	288	395	537	528	681
Exoc3l	ENSMUSG00000043251	589.7151442	-0.772386375	0.204640136	-3.774364063	0.000160416	0.01355741	758	678	796	353	513	441
Amigo1	ENSMUSG00000050947	1511.529133	-0.577406641	0.153117259	-3.771009508	0.000162588	0.013579688	1862	1792	1777	1315	1249	1075
Rnf144a	ENSMUSG00000020642	607.6727328	-1.120551053	0.297176411	-3.770659487	0.000162817	0.013579688	1035	917	545	459	391	298
Lrrc55	ENSMUSG00000075224	1620.575009	0.856840215	0.227247585	3.770514064	0.000162912	0.013579688	1313	1299	847	2134	1873	2258
Mmp24	ENSMUSG00000027612	127.4578784	-1.231622212	0.327340639	-3.762509335	0.000168217	0.01395819	184	238	115	77	64	88
Hoxb9	ENSMUSG00000020875	7376.084465	0.590792812	0.157221516	3.757709668	0.000171476	0.014164201	5219	5963	6478	8781	8359	9457
Rasd2	ENSMUSG00000034472	172.5018823	1.474397012	0.393597446	3.745951672	0.000179711	0.014777602	63	76	135	250	352	158
Kif23	ENSMUSG00000032254	81.68690395	1.228884664	0.328743626	3.738124686	0.000185398	0.015162759	67	42	37	89	126	128
Cdh6	ENSMUSG00000039385	121.4153391	2.337397534	0.625435283	3.737233249	0.000186056	0.015162759	63	49	8	236	283	90
Adcyap1r1	ENSMUSG00000029778	231.4571377	-0.968172933	0.259281201	-3.734065293	0.000188414	0.015221437	371	316	232	153	184	133
Zcchc16	ENSMUSG00000071679	32.64205618	-1.498650534	0.401349921	-3.73402474	0.000188444	0.015221437	56	39	49	11	21	18
Tacc3	ENSMUSG00000037313	153.9300181	0.860299699	0.230707736	3.728959053	0.000192272	0.015462258	126	106	96	170	207	219
Cenpe	ENSMUSG00000045328	85.67547363	1.010855283	0.271272196	3.726350496	0.000194215	0.015554558	68	59	43	120	117	106
Lama3	ENSMUSG00000024421	1989.579411	0.597820451	0.160813089	3.717486273	0.000201215	0.01597681	1631	1651	1468	2122	2628	2438
Synpr	ENSMUSG00000056296	57.37625275	-1.336628023	0.359561469	-3.717383918	0.000201296	0.01597681	103	61	83	28	26	43
2810417H13Rik	ENSMUSG00000040204	57.107781	1.066505946	0.287043813	3.715481394	0.000202817	0.016018115	36	34	40	68	84	80
St3gal6	ENSMUSG00000022747	191.0852773	-0.800271397	0.215442798	-3.714542353	0.000203572	0.016018115	273	221	235	120	141	157
Lrrc2	ENSMUSG00000032495	85.77068688	1.078655216	0.29088526	3.708181078	0.000208753	0.016304089	52	64	49	88	125	136
Il34	ENSMUSG00000031750	231.7039144	-0.740274272	0.199648328	-3.707891172	0.000208992	0.016304089	265	268	337	179	167	175
Acan	ENSMUSG00000030607	3654.977925	-0.679796281	0.18376976	-3.699173786	0.000216302	0.016802559	4281	5339	3882	2977	2628	2823
1700025G04Rik	ENSMUSG00000032666	4961.779424	0.727533067	0.197006128	3.692946375	0.000221671	0.017110404	3628	4069	3513	7568	5566	5427
Eef1a2	ENSMUSG00000016349	166.7423519	-1.141287014	0.30916266	-3.691542227	0.000222898	0.017110404	285	155	249	80	113	120
Kif2c	ENSMUSG00000028678	44.20580038	1.460649903	0.395696583	3.691338177	0.000223077	0.017110404	28	30	13	62	54	78
Hmga2	ENSMUSG00000056758	147.9209747	0.876111011	0.237964944	3.681680992	0.000231701	0.017697513	100	120	93	203	213	159
Slc6a2	ENSMUSG00000055368	61.78386139	-1.431139243	0.389661229	-3.672778137	0.000239928	0.01819126	125	67	79	22	43	36
Slain1	ENSMUSG00000055717	190.4866973	0.946079493	0.257609557	3.672532585	0.000240159	0.01819126	143	101	147	226	305	222
Angptl6	ENSMUSG00000038742	30.32942735	-1.422708979	0.388252404	-3.664391942	0.000247927	0.018638089	48	49	35	17	15	18
Ngfr	ENSMUSG00000000120	49.93614471	-1.389112975	0.379102597	-3.664213819	0.000248099	0.018638089	84	47	86	22	27	34
Cd47	ENSMUSG00000055447	2064.884265	-0.621043217	0.169793094	-3.657647095	0.000254541	0.019043645	2724	2649	2134	1720	1635	1527
Cdca8	ENSMUSG00000028873	92.37883761	1.059107792	0.289647218	3.656543987	0.000255639	0.019047681	64	45	71	105	121	149
Ghdc	ENSMUSG00000017747	474.9103136	-0.543469725	0.149000437	-3.647437131	0.000264869	0.019655225	554	568	569	393	368	398
Adamts15	ENSMUSG00000033453	27.8251561	-1.50775816	0.413875794	-3.643020882	0.000269457	0.019914725	43	47	33	12	18	13
Adamts20	ENSMUSG00000022449	11.57774148	2.412816098	0.663484881	3.636580374	0.000276281	0.020336767	2	4	5	22	16	21
Tmem180	ENSMUSG00000025227	136.2915521	-1.262528949	0.347286881	-3.635406397	0.000277543	0.020347562	155	173	249	53	74	114
Arhgef19	ENSMUSG00000028919	1846.503445	1.193622077	0.328504268	3.633505536	0.000279596	0.020383907	3540	2749	1420	1225	1178	967
Fgf20	ENSMUSG00000031603	192.8671306	1.591993754	0.438217728	3.632883043	0.000280272	0.020383931	57	66	165	197	293	379
Crispld2	ENSMUSG00000031825	226.5560155	-0.847089285	0.233476222	-3.62816084	0.000285447	0.020677947	265	262	347	148	145	193
Ass1	ENSMUSG00000076441	219.9798425	0.900059415	0.248327555	3.62448467	0.000289538	0.020891382	116	185	159	273	258	328
RP23-380L19.3	ENSMUSG00000107881	1033.549866	-0.953163082	0.263162471	-3.62195673	0.000292383	0.021013592	1856	1215	1018	784	680	648
Fbln1	ENSMUSG00000006369	3108.106034	0.797337294	0.220389379	3.617857167	0.000297052	0.021265446	2731	2368	1712	4033	4221	3584
Phf19	ENSMUSG00000026873	52.18691151	1.27408016	0.352322458	3.616233174	0.000298921	0.021315646	32	40	19	73	82	67
Ccnb1	ENSMUSG00000041431	83.28288723	1.174580006	0.325197382	3.611898717	0.000303963	0.021554435	66	55	32	103	110	133
Gm7329	ENSMUSG00000100774	913.0940498	-0.566108781	0.156759104	-3.611329536	0.000304631	0.021554435	989	1100	1181	782	720	706
Usp2	ENSMUSG00000032010	1084.096811	1.031151942	0.285775767	3.608255357	0.000308263	0.021727186	439	811	887	1619	1350	1399
Scn8a	ENSMUSG00000023033	198.9648604	1.152970956	0.319697146	3.606447453	0.000310418	0.021759083	102	117	151	182	269	372
Ccnl1	ENSMUSG00000027829	851.4275089	0.665451017	0.184595303	3.604918468	0.000312251	0.021759083	761	561	653	965	1080	1089
Armcx1	ENSMUSG00000033460	663.2327085	-0.566954937	0.157274078	-3.604884813	0.000312291	0.021759083	808	719	848	527	516	561
Clip4	ENSMUSG00000024059	184.8497947	0.780436076	0.216777488	3.600171232	0.000318008	0.022056982	127	129	152	198	251	253
Sfxn2	ENSMUSG00000025036	163.1923314	-1.147089815	0.318691428	-3.599374549	0.000318983	0.022056982	219	210	246	71	83	150
B4galt5	ENSMUSG00000017929	14880.88623	0.746992802	0.207782417	3.595072263	0.000324302	0.022340093	12323	12269	8745	20415	19329	16204
Epn3	ENSMUSG00000010080	42.17054742	1.727815478	0.481670108	3.587134534	0.000334332	0.022944466	25	26	8	42	76	76
Trabd2b	ENSMUSG00000070867	128.3530385	-1.185526392	0.330753054	-3.584324858	0.000337951	0.023105993	194	116	225	72	99	64
Alpk1	ENSMUSG00000028028	138.9228627	0.838411803	0.234099453	3.581434266	0.000341713	0.023276019	103	116	80	187	168	179
Rep15	ENSMUSG00000040121	62.09524815	-1.106019869	0.309018601	-3.579136875	0.000344731	0.023394284	72	97	86	49	39	31
Mxd1	ENSMUSG00000020000	15.74674337	-2.666626943	0.745646215	-3.576262963	0.000348541	0.023565248	44	29	9	4	6	3
Grin3b	ENSMUSG00000035745	9.445762791	-3.103477884	0.868623289	-3.572832334	0.000355141	0.02378143	8	24	19	1	2	3
Rasl11a	ENSMUSG00000029641	70.1133221	-1.144264003	0.320363163	-3.571771455	0.000354575	0.023796921	102	73	115	34	44	53
Gm10184	ENSMUSG00000066878	186.5631671	0.922576539	0.258541779	3.568384736	0.000359189	0.024018284	109	109	168	229	226	278
Abca1	ENSMUSG00000015243	3616.26977	-1.262845836	0.354398613	-3.563348696	0.000366154	0.024394661	6512	5778	3026	2797	2208	1377
Zp3	ENSMUSG00000004948	10.01205374	3.170878026	0.891818509	3.555519417	0.000377233	0.024954979	1	0	5	12	21	20
Slc6a17	ENSMUSG00000027894	27.33425683	-1.564812486	0.44012311	-3.555397234	0.000377408	0.024954979	54	39	29	11	16	14
Alas1	ENSMUSG00000032786	19926.12601	0.847809454	0.23856006	3.553861667	0.000379619	0.024954979	11905	17691	13106	30033	20261	26560
Rsad2	ENSMUSG00000020641	1214.539784	-0.534825618	0.150576374	-3.551856141	0.000382524	0.024954979	1458	1530	1324	982	1058	935
Top2a	ENSMUSG00000020914	274.6176256	0.970314978	0.273194111	3.551741931	0.00038269	0.024954979	227	210	120	365	350	376
Tmem59l	ENSMUSG00000035964	78.84139826	-1.261594749	0.355265393	-3.551133249	0.000383576	0.024954979	112	81	141	43	33	63
Cyp4f14	ENSMUSG00000024292	160.8807066	-0.952437423	0.268235456	-3.550751405	0.000384133	0.024954979	175	272	190	103	97	128
Bhmt	ENSMUSG00000074768	15.59651401	-2.650916364	0.74728542	-3.547394736	0.000389061	0.025185504	49	22	10	4	5	4
Igf2	ENSMUSG00000048583	100.9908325	-1.406127338	0.396691927	-3.544633107	0.00039316	0.0253609	213	75	152	53	51	62
Tmprss13	ENSMUSG00000037129	28.38359611	1.858578106	0.52484567	3.541189745	0.000398327	0.025603731	17	15	5	30	43	61
Smim1	ENSMUSG00000078350	906.9765866	-0.544687388	0.154265558	-3.530842494	0.000414238	0.026533056	1017	1095	1116	812	696	705
Fam110b	ENSMUSG00000049119	156.874744	0.748693407	0.212140065	3.529240958	0.000416753	0.026600821	125	118	108	221	191	177

Table S4. Genes differentially expressed ABC_Hoxb9 Male vs ABC Male

								Read count					
Gene	ENSEMBL_Gene_ID	baseMean	log2FoldChange	lfcSE	stat	pvalue	padj	166836_Norma	166838_Norma	182098_Turq19	169229_Norma	182083_Turq19	182086_Turq19
Nell2	ENSMUSG00000022454	13.61191197	2.283414696	0.647390655	3.527104816	0.00042013	0.026722929	3	8	3	16	24	28
Nuf2	ENSMUSG00000026683	81.95103339	1.140469165	0.323889877	3.52116335	0.000429658	0.027234042	63	52	38	82	131	125
Bcl2	ENSMUSG00000057329	468.7333698	-0.571779987	0.162802988	-3.51209761	0.000444585	0.028082676	610	529	542	385	392	354
Prr5	ENSMUSG00000036106	164.6942232	-0.834666144	0.237868612	-3.50893772	0.0004499	0.028320445	179	192	262	117	117	122
Dusp14	ENSMUSG00000018648	244.0798448	1.017915861	0.290237444	3.507183102	0.000452877	0.028409887	134	154	197	245	299	436
Spry1	ENSMUSG00000037211	947.0523941	-0.554648395	0.158275488	-3.504322756	0.00045777	0.028618475	1119	1232	1029	796	792	714
Slc29a1	ENSMUSG00000023942	2721.780601	-0.654796013	0.187242401	-3.497049857	0.000470434	0.029296947	3251	3928	2808	2251	2127	1965
Mlxipl	ENSMUSG00000005373	60.79210579	-1.017836953	0.291195833	-3.495369227	0.000473406	0.029296947	82	92	71	38	47	36
Syn2	ENSMUSG00000009394	98.57744984	-0.964846232	0.276036949	-3.495351748	0.000473437	0.029296947	161	122	108	78	59	64
Cd55	ENSMUSG00000026399	111.840714	-1.623283819	0.464812527	-3.492340947	0.000478807	0.029529106	195	75	237	45	40	80
Colec12	ENSMUSG00000036103	873.3304301	-0.490763072	0.140767221	-3.486344835	0.000489669	0.030097354	1015	1076	970	707	726	745
Akr1b7	ENSMUSG00000052131	8573.060795	-0.909560066	0.261100182	-3.4835674	0.000494779	0.030149578	10965	14517	8086	5885	5022	6963
Utp18	ENSMUSG00000054079	639.3703159	0.502137816	0.144261483	3.48074764	0.000500016	0.030149578	519	513	557	758	725	766
Hand1	ENSMUSG00000037335	91.16387527	-1.57388594	0.452172477	-3.480720348	0.000500067	0.030149578	146	64	200	30	50	57
Nptx1	ENSMUSG00000025582	68.23170099	-1.55352757	0.446357496	-3.480455876	0.000500561	0.030149578	146	89	71	55	28	21
Smad6	ENSMUSG00000036867	224.7208814	-0.61200645	0.175866017	-3.479958558	0.000501491	0.030149578	258	278	279	181	172	180
Cdk5rap2	ENSMUSG00000039298	3280.344347	0.44560483	0.128062702	3.47958323	0.000502194	0.030149578	2699	2708	2926	3789	3888	3672
A730017C20Rik	ENSMUSG00000050875	69.50165731	-0.930487092	0.267438293	-3.479259017	0.000502802	0.030149578	83	88	103	50	44	50
Kif11	ENSMUSG00000012443	77.36950602	1.158681146	0.333156437	3.47788911	0.000505379	0.030149578	69	42	32	109	113	99
Ncapg	ENSMUSG00000015880	38.86786156	1.471402227	0.423259075	3.476363094	0.000508264	0.030149578	32	17	13	47	58	67
Phox2a	ENSMUSG00000007946	88.09303764	-1.355275759	0.389867473	-3.476247317	0.000508483	0.030149578	133	77	170	36	45	68
Tshz2	ENSMUSG00000047907	1678.800815	-0.68503661	0.19706818	-3.476140144	0.000508686	0.030149578	1967	2467	1776	1444	1219	1201
Sh3bp5	ENSMUSG00000021892	1436.020814	-0.480246356	0.138233521	-3.474167139	0.000512442	0.030224382	1603	1711	1705	1137	1275	1185
Resp18	ENSMUSG00000033061	165.9197902	-1.408043716	0.405339514	-3.473739095	0.00051326	0.030224382	231	158	335	53	84	135
Hand2	ENSMUSG00000038193	200.1790172	-1.542684988	0.444301109	-3.472161012	0.000516287	0.030243509	268	158	469	62	99	146
Aunip	ENSMUSG00000078521	40.59140912	1.432524602	0.412612122	3.471843226	0.000516898	0.030243509	17	21	28	39	59	79
Slc35d3	ENSMUSG00000050473	69.28962292	-1.287917397	0.371834442	-3.463685044	0.00053283	0.031076074	100	69	126	43	26	51
1500015O10Rik	ENSMUSG00000026051	4188.267013	1.419620717	0.410088025	3.461746333	0.000536683	0.031201093	1991	2558	2289	3275	4290	10727
Met	ENSMUSG00000009376	73.12490383	-1.266246211	0.366271548	-3.457124143	0.000545973	0.031640451	132	102	77	29	59	41
Iqsec3	ENSMUSG00000040797	39.95733316	-1.390605471	0.402564127	-3.454370064	0.00055158	0.031864214	83	48	42	21	21	24
Nsg2	ENSMUSG00000020297	97.3894001	-1.088555016	0.315393558	-3.451418038	0.000557649	0.032070023	147	101	150	45	63	79
Gars	ENSMUSG00000029777	4635.864874	-0.466222678	0.135100546	-3.450931127	0.000558656	0.032070023	5758	5080	5297	3747	3878	4054
Armcx4	ENSMUSG00000049804	360.8250109	-1.093167523	0.317013344	-3.448332835	0.000564058	0.032278645	642	542	289	253	241	198
Gm10644	ENSMUSG00000074219	29.08878472	-1.409587022	0.409247807	-3.444336164	0.000572463	0.032657252	49	40	37	22	13	14
Phox2b	ENSMUSG00000012520	68.01926807	-1.068556228	0.310520021	-3.44118304	0.000579177	0.032937286	118	84	75	48	47	38
Adcy8	ENSMUSG00000022376	92.45511696	-1.574900999	0.458354198	-3.435991217	0.00059039	0.033344955	146	175	94	80	24	36
Necab3	ENSMUSG00000027489	22.08095154	1.650535451	0.480454005	3.435366203	0.000591754	0.033344955	9	8	15	42	28	30
Epha8	ENSMUSG00000028661	23.74348406	1.849170855	0.538279865	3.435333504	0.000591825	0.033344955	11	8	12	31	58	22
Guca1b	ENSMUSG00000023979	76.18577868	-1.328464587	0.387722737	-3.426326236	0.000611805	0.03436462	152	109	66	54	44	33
Gm14519	ENSMUSG00000082838	126.0752393	0.798930055	0.233577721	3.420403507	0.000625283	0.035006359	86	109	81	171	162	148
Inmt	ENSMUSG00000003477	38.8225443	-1.26867365	0.37099743	-3.419629214	0.000627065	0.035006359	69	50	45	25	17	26
Col4a5	ENSMUSG00000031274	438.8534776	-0.986248177	0.28864438	-3.416827917	0.000633553	0.035135728	794	548	407	339	294	250
Pdgfrl	ENSMUSG00000031595	113.6037061	-0.838010651	0.24527571	-3.416606771	0.000634068	0.035135728	140	143	154	87	64	94
Antxr2	ENSMUSG00000029338	1545.204258	-0.697510279	0.204180853	-3.416139518	0.000635157	0.035135728	2200	2034	1500	1207	1249	1081
Ncaph	ENSMUSG00000034906	93.90602917	0.910847209	0.266859973	3.413202804	0.000642041	0.035409264	75	52	69	105	127	136
Get4	ENSMUSG00000025858	2442.791016	-0.447955161	0.131360352	-3.410124541	0.000649332	0.035626261	2961	2750	2746	2136	1974	2090
Gpr55	ENSMUSG00000049608	296.214638	0.938693834	0.275331808	3.409318532	0.000651254	0.035626261	231	247	131	376	372	420
Gm21781	ENSMUSG00000095123	102.5744931	0.944872341	0.277163685	3.4090769993	0.000651831	0.035626261	87	64	59	111	156	138
9530077C05Rik	ENSMUSG00000036411	217.536501	-0.693344812	0.20359524	-3.405506001	0.000660416	0.035987723	243	261	302	172	148	179
Prc1	ENSMUSG00000038943	168.2720505	1.025376904	0.302098913	3.394176073	0.000688354	0.03739853	138	128	67	219	224	233
Scg5	ENSMUSG00000023236	110.2920724	-1.003666853	0.295840031	-3.392599872	0.000692327	0.037438817	169	108	164	68	61	91
Gatm	ENSMUSG00000027199	286.7730806	-0.851599815	0.251042351	-3.392255575	0.000693197	0.037438817	336	483	288	211	192	210
Lynx1	ENSMUSG00000022594	5861.881691	-0.973976888	0.287428105	-3.388593087	0.000702522	0.037728555	9207	9175	4924	4774	3601	3490
Klf15	ENSMUSG00000030087	475.1878637	-0.774197089	0.228476117	-3.388525233	0.000702696	0.037728555	500	532	767	375	343	335
Nek2	ENSMUSG00000026622	75.56922442	1.067293405	0.315601955	3.38177058	0.000720203	0.038479467	61	54	31	109	94	104
Crabp1	ENSMUSG00000032291	33.5064764	-1.329660683	0.393306531	-3.380723632	0.000722952	0.038479467	42	39	63	16	21	20
Wnk2	ENSMUSG00000037989	255.1720623	1.076091597	0.318304041	3.380703533	0.000723005	0.038479467	151	231	111	418	347	274
Clstn3	ENSMUSG00000008153	147.3542931	0.889775162	0.263281531	3.37955784	0.000726025	0.038527883	125	109	76	169	213	193
Wnt6	ENSMUSG00000033227	230.8433165	-1.034985636	0.306768919	-3.37382822	0.000741306	0.039146524	320	385	226	107	190	157
Ehd2	ENSMUSG00000074364	2054.028249	-0.553140756	0.16396249	-3.373581081	0.000741972	0.039146524	2570	2666	2093	1607	1704	1684
Igsf3	ENSMUSG00000042035	665.2679837	-0.598565631	0.17783321	-3.365882179	0.000762993	0.040139584	908	777	719	577	518	493
Slc16a2	ENSMUSG00000033965	1021.203823	-0.691146979	0.2058617	-3.357336394	0.000786973	0.041282162	1480	1188	1116	837	856	650
Cep85	ENSMUSG00000037443	346.9467243	1.179983852	0.351913433	3.353051463	0.000799258	0.041745969	269	234	135	301	607	536
Fstl3	ENSMUSG00000020325	262.7154706	-0.850355507	0.253636036	-3.352660345	0.000800388	0.041745969	272	440	302	196	201	166
Rab39b	ENSMUSG00000031202	70.64140472	-1.034501303	0.309230329	-3.345406986	0.000821619	0.042731217	104	110	71	39	46	54
Sgsm1	ENSMUSG00000042216	55.40361214	-1.17734905	0.352060756	-3.344164404	0.000825308	0.042801136	104	64	63	34	40	28
Tk1	ENSMUSG00000025574	76.38705687	1.217997085	0.364941293	3.337515126	0.000845311	0.043714323	61	31	46	79	102	140
Hoxc5	ENSMUSG00000022485	181.7899307	-0.807079908	0.241900626	-3.336411	0.000848676	0.04376435	201	216	277	148	140	109
Me1	ENSMUSG00000032418	31551.28546	-0.485930698	0.145698292	-3.335184583	0.000852428	0.043834007	35974	38107	36365	29261	25318	24284
Cplx1	ENSMUSG00000033615	42.91110991	-1.3840753	0.41591056	-3.32781957	0.000875285	0.044882961	63	82	41	27	16	29
Tbx20	ENSMUSG00000031965	61.51433865	-1.134772696	0.341277864	-3.325069728	0.000883964	0.045168247	113	61	80	34	38	43
Asic4	ENSMUSG00000033007	32.04524737	-1.34355914	0.404139671	-3.324492092	0.000885797	0.045168247	44	34	60	17	19	18
Sept6	ENSMUSG00000050379	794.993808	0.46163409	0.138955174	3.322179918	0.000893171	0.045292297	682	687	637	906	913	945
Tmem130	ENSMUSG00000043388	87.56806649	-0.997593985	0.300283559	-3.322173179	0.000893192	0.045292297	148	102	101	46	62	68
Acot2	ENSMUSG00000021226	281.658112	-0.630269099	0.189794631	-3.320795186	0.000897614	0.045390416	311	341	375	199	222	242
Clspn	ENSMUSG00000042489	35.27511195	1.307023474	0.393788105	3.319103492	0.00090307	0.04554015	28	15	18	57	41	53
Dpep1	ENSMUSG00000019278	634.8936786	-0.742600922	0.223902005	-3.316633636	0.00091109	0.04581804	780	968	636	411	501	513
Aass	ENSMUSG00000029695	32.01398893	-1.6179263	0.488098812	-3.314751564	0.000917246	0.046000897	46	34	65	9	25	13
Abca5	ENSMUSG00000018800	262.9986447	-1.336083207	0.40341701	-3.311915897	0.000926594	0.046263456	498	464	168	173	159	116
Cartpt	ENSMUSG00000021647	59.96831123	-2.078704103	0.627698668	-3.311627393	0.00092755	0.046263456	79	30	182	18	16	36
Btg1	ENSMUSG00000036478	3447.653727	0.904419178	0.273213949	3.310296496	0.000931972	0.046357351	1784	2222	3197	3734	4248	5501
Maf	ENSMUSG00000055435	941.5897672	0.57739252	0.174486322	3.309099028	0.000935967	0.046429571	711	750	806	1034	1048	1301
Kctd14	ENSMUSG00000051727	184.9645915	-1.126259844	0.340688589	-3.305833777	0.000946942	0.046846706	176	199	386	123	121	104
Sel1l3	ENSMUSG00000029189	271.4631924	1.019383438	0.308581808	3.303446321	0.000955043	0.047050672	190	110	238	324	425	341
Dync1i1	ENSMUSG00000029757	66.09248706	-1.510702336	0.45743677	-3.302538043	0.000958141	0.047050672	166	61	67	36	40	27
Vasn	ENSMUSG00000039646	241.538194	0.640405418	0.193924392	3.302345885	0.000958798	0.047050672	278	300	306	180	217	170
Spock1	ENSMUSG00000056222	61.91828175	-1.392395894	0.421933769	-3.30003426	0.00096673	0.047312764	134	84	51	27	43	33
Rgl1	ENSMUSG00000026482	836.9410854	-0.603175345	0.182990956	-3.296203035	0.000980012	0.047834524	1042	1112	874	681	723	589
Gucy1a3	ENSMUSG00000033910	574.4009867	-0.979309212	0.297251224	-3.294550643	0.000985792	0.047988345	624	547	1116	379	403	378
Dtx1	ENSMUSG00000029603	744.0286305	-0.633677776	0.192400539	-3.293534314	0.000989363	0.048034083	1079	827	809	535	613	602
Scx	ENSMUSG00000034161	602.564982	-1.227580647	0.372933576	-3.291687111	0.000995883	0.048222419	672	572	1290	261	320	501
Sidt1	ENSMUSG00000022696	45.72023134	-1.341397404	0.40768545	-3.290275389	0.001000894	0.048336813	92	53	51	35	19	23
Il11ra1	ENSMUSG00000073889	1325.884139	-0.475465707	0.144696461	-3.285952563	0.001016381	0.04895525	1404	1580	1643	1108	1091	1128
Napepld	ENSMUSG00000044968	1438.374367	0.716556561	0.218339985	3.281838464	0.001031327	0.049544391	1011	1309	945	2098	1765	1502
Tmem221	ENSMUSG00000043664	178.0759265	-1.006999706	0.30790796	-3.270456885	0.001073739	0.051446475	179	216	319	148	115	93
Rasl11b	ENSMUSG00000049907	1368.19941	0.591683537	0.180972572	3.269465259	0.00107751	0.051491987	1030	1180	1065	1374	1807	1753
Cap2	ENSMUSG00000021373	591.515574	0.829331115	0.253974119	3.265415854	0.001093035	0.052097518	492	432	355	556	924	791
Slc25a22	ENSMUSG00000019082	751.4514484	0.591417403	0.181392822	3.260423404	0.00111246	0.052779587	518	585	696	868	957	884
Matn2	ENSMUSG00000022324	424.8002674	-0.688023603	0.211033785	-3.260253335	0.001113127	0.052779587	584	470	519	262	346	368
Nup88	ENSMUSG00000040667	2172.674635	0.476088098	0.146261957	3.2550371114	0.001133775	0.053446284	1855	1704	1893	2688	2582	2314
Cdt1	ENSMUSG00000006585	112.3390465	0.694711965	0.213440075	3.254833771	0.001134588	0.053446284	83	87	88	143	135	139
Steap2	ENSMUSG00000015653	10967.77663	0.913912757	0.280816179	3.254487544	0.001135971	0.053446284	8259	9435	5123	17713	13448	11829
RP23-308E4.9	ENSMUSG00000107568	84.50379391	0.866591901	0.266484443	3.251941805	0.001146195	0.053788656	53	66	60	125	112	91
Slc30a2	ENSMUSG00000028836	3078.378764	-0.672352883	0.207048821	-3.247315677	0.001164991	0.054530539	4430	3699	3220	2645	2505	1971
Lgr5	ENSMUSG00000020140	132.0893953	-1.026777489	0.316769315	-3.241404517	0.001189423	0.055463868	254	151	127	84	87	90
Mtmr11	ENSMUSG00000045934	672.1976013	-0.666613106	0.205679714	-3.241025048	0.001191007	0.055463868	999	692	783	473	545	541
Prpf40b	ENSMUSG00000023007	604.4310571	0.480369579	0.148468688	3.235494207	0.001214323	0.05540579	539	502	474	716	696	700
Mmd2	ENSMUSG00000039533	387.3046457	-1.731898603	0.535581742	-3.233677454	0.001222074	0.056621723	722	742	322	85	351	101
Arntl	ENSMUSG00000055116	328.809126	-2.78614742	0.862417856	-3.230623531	0.001235205	0.056976924	1194	409	120	76	96	78
Fads3	ENSMUSG00000024664	327.9514307	-0.874320832	0.270650388	-3.23044367	0.001235983	0.056976924	447	539	287	234	245	216
Pla2g2f	ENSMUSG00000028749	461.0928548	1.196445579	0.370647615	3.227986715	0.001246648	0.05732381	269	396	175	538	463	925
Dnajb9	ENSMUSG00000014905	1952.175798	-0.656517951	0.203822778	-3.221023473	0.001277337	0.058383395	2092	2876	2198	1738	1413	1396
Srcin1	ENSMUSG00000038453	1049.355614	-0.439405148	0.136422115	-3.220923144	0.001277784	0.058383395	1230	1227	1167	935	879	859
Jag1	ENSMUSG00000027276	341.7516527	-0.558649573	0.173504214	-3.219804068	0.001282782	0.058383395	422	438	362	283	277	270
Odf2	ENSMUSG00000026790	1272.205217	0.546010941	0.169583251	3.219722106	0.001283149	0.058383395	1133	1029	941	1608	1589	1333
Tet2	ENSMUSG00000040943	1284.376006	0.711993963	0.221174061	3.219156714	0.001285682	0.058383395	1231	815	875	1420	1622	1744
Ssc4d	ENSMUSG00000029699	553.4844018	1.697379349	0.527757693	3.216209582	0.001298959	0.058839956	142	105	535	789	1025	724
Cndp2	ENSMUSG00000024644	1559.564392	0.482893455	0.150461307	3.209419504	0.001330033	0.060098389	1176	1429	1298	1875	1788	1792
Rab3b	ENSMUSG00000003411	97.75514943	-1.088140072	0.33950002	-3.205125206	0.001350037	0.060435694	159	82	158	58	55	74
Cxx1c	ENSMUSG00000051851	972.4206512	0.660456214	0.206095615	3.204610711	0.001352453	0.060435694	676	760	825	979	1172	1422
Diap2	ENSMUSG00000034480	1084.025864	-0.678617184	0.211775481	-3.204418096	0.001353358	0.060435694	1479	1429	1095	958	843	700
Gng3	ENSMUSG00000071658	37.3594843	-1.482023658	0.462545309	-3.204061588	0.001355035	0.060435694	55	31	80	14	21	23
Proca1	ENSMUSG00000044122	129.4778096	-0.848549016	0.264884548	-3.203467407	0.001357834	0.060435694	139	149	212	100	82	95
Scube1	ENSMUSG00000016763	154.0113496	-0.963322035	0.30072732	-3.203307349	0.001358589	0.060435694	166	160	285	98	117	98
Gsta3	ENSMUSG00000025934	512.8735063	-0.847501657	0.264607268	-3.20286613	0.001360672	0.060435694	676	633	668	500	263	337
Cenpa	ENSMUSG00000029177	89.42184452	1.091661351	0.341272589	3.198795875	0.001380028	0.061146648	63	62	46	82	117	167
Ccdc92	ENSMUSG00000037979	232.6839777	0.721822512	0.225756001	3.197356912	0.001386932	0.061216459	176	208	143	255	317	298
Dgat2	ENSMUSG00000030747	209.1468777	1.168741251	0.365566327	3.19707031	0.001388311	0.061216459	100	125	161	204	447	218
Eya1	ENSMUSG00000025932	42.11086384	-1.110872664	0.348192841	-3.1903949	0.001420785	0.062422135	65	46	62	23	33	24
Gjc2	ENSMUSG00000043448	47.41055649	-1.019513855	0.319592036	-3.190047745	0.001422493	0.062422135	53	70	68	31	36	27
Cers4	ENSMUSG00000008206	1498.293388	-0.556335797	0.174534556	-3.18753953	0.001434888	0.06281508	1757	1539	2055	1274	1170	1195
Pcdh15	ENSMUSG00000052613	22.77243325	-1.7190338	0.539720257	-3.185045916	0.00144731	0.063207301	54	29	22	14	10	8
Ptpn7	ENSMUSG00000031506	57.32514387	-0.985999859	0.309683792	-3.18389236	0.00145309	0.063308268	87	62	80	32	38	45
Ank3	ENSMUSG00000069601	103.4614686	-0.755016742	0.237274143	-3.182043912	0.001462396	0.063562017	131	137	122	69	72	90
Lin9	ENSMUSG00000058729	219.388108	0.588303944	0.185143896	3.177549765	0.001485252	0.064402078	165	180	180	285	244	262
Vstm4	ENSMUSG00000050666	1404.748128	-0.46013167	0.144863519	-3.176311555	0.001491607	0.064524358	1580	1539	1762	1177	1238	1133
Slc27a6	ENSMUSG00000024600	162.5107817	-1.551854058	0.488740198	-3.175212646	0.001497267	0.064616112	326	270	131	140	70	39
Ramp1	ENSMUSG00000034353	69.55992632	-1.289199232	0.406877717	-3.168517659	0.001532184	0.065967043	84	61	151	32	41	48
Tlx2	ENSMUSG00000068327	28.03054411	-1.484030568	0.468628618	-3.166751903	0.001541518	0.06621272	41	29	54	10	13	21
Gcat	ENSMUSG00000006378	478.5084463	-0.574067188	0.181372148	-3.165134201	0.001550114	0.066281161	536	653	528	388	412	354
Magt1	ENSMUSG00000031232	1761.342598	-0.723387819	0.228566753	-3.164886451	0.001551435	0.066281161	2151	2721	1709	1494	1168	1324
5830444B04Rik	ENSMUSG00000084803	95.32042562	-1.056726544	0.33394162	-3.164405036	0.001554004	0.066281161	142	127	117	91	52	42
Serpina5	ENSMUSG00000041550	10.37133763	5.836319044	1.846537607	3.160682469	0.001574	0.066977545	1	0	0	1	59	1

Table S4. Genes differentially expressed ABC_Hoxb9 Male vs ABC Male

								Read count					
Gene	ENSEMBL_Gene_ID	baseMean	log2FoldChange	lfcSE	stat	pvalue	padj	166836_Norma	166838_Norma	182098_Turq19	169229_Norma	182083_Turq19	182086_Turq19
Kcnq1	ENSMUSG00000009545	116.3147942	-0.802057493	0.253964108	-3.158152931	0.001587722	0.067299806	141	171	131	87	98	69
Sh2d4a	ENSMUSG00000053886	190.8689087	-0.935704906	0.296303405	-3.157928292	0.001588946	0.067299806	314	279	159	140	126	127
Ckap2l	ENSMUSG00000048327	72.49704332	1.037498647	0.328763915	3.155755848	0.001600828	0.067585075	61	50	31	95	84	114
Gm6665	ENSMUSG00000091561	419.184502	-0.810904844	0.256994064	-3.155344649	0.001603086	0.067585075	485	423	695	264	293	356
Chd1	ENSMUSG00000023852	2851.62008	0.559219325	0.17747365	3.150999176	0.001627129	0.068415904	2262	2468	2187	3986	3201	3005
Kif20a	ENSMUSG00000003779	156.6086389	1.024186009	0.325093768	3.150432616	0.001630289	0.068415904	134	115	60	197	188	245
Gm4980	ENSMUSG00000096606	1371.863761	-0.814338894	0.258755164	-3.147140641	0.001648756	0.06875129	2178	1812	1257	1140	816	1028
Dtx4	ENSMUSG00000039982	406.4411044	-0.863187192	0.27429036	-3.146983336	0.001649644	0.06875129	684	531	358	265	320	280
Cadm1	ENSMUSG00000032076	1312.717482	0.509003997	0.161748964	3.146876403	0.001650247	0.06875129	1110	1008	1132	1462	1733	1430
Stmn1	ENSMUSG00000028832	558.8057251	0.76784998	0.244097832	3.145664891	0.001657098	0.06875129	376	357	508	652	589	871
Gm10038	ENSMUSG00000058281	9.40749792	2.220378256	0.705853946	3.145662454	0.001657111	0.06875129	5	3	2	16	14	17
Nova1	ENSMUSG00000021047	142.2760962	-0.949480859	0.301910627	-3.144907052	0.001661396	0.068772755	157	162	244	90	77	124
Unc5c	ENSMUSG00000059921	39.23919115	-1.245444395	0.396176427	-3.143661027	0.001668486	0.068844244	72	43	50	16	27	27
C7	ENSMUSG00000079105	327.1272933	-1.569649821	0.499367016	-3.143278929	0.001670666	0.068844244	757	574	137	167	159	169
Ampd3	ENSMUSG00000005686	483.3639867	-0.674553572	0.214854799	-3.139578805	0.001691909	0.069418024	557	501	726	345	414	359
Tia1	ENSMUSG00000071337	581.6717161	0.580307543	0.184874868	3.138920662	0.001695714	0.069418024	553	427	419	674	699	718
Gstm7	ENSMUSG00000004035	789.8977761	-0.592606859	0.188809595	-3.138648016	0.001697292	0.069418024	888	855	1107	560	676	654
Grb14	ENSMUSG00000026888	1388.92635	0.874278577	0.278591023	3.138215178	0.0016998	0.069418024	999	764	1179	1233	2124	2035
Prr16	ENSMUSG00000073565	280.0366035	0.915500518	0.291905294	3.136292958	0.001710982	0.069589349	166	233	184	504	314	281
Osbpl6	ENSMUSG00000042359	151.3663739	-0.965462596	0.307846318	-3.136183674	0.00171162	0.069589349	242	231	128	119	95	94
Gm43843	ENSMUSG00000106944	97.60850072	0.866750797	0.27660699	3.133510097	0.00172729	0.069823582	89	64	54	118	120	140
Pon2	ENSMUSG00000032667	1884.132818	-0.621646421	0.198394051	-3.13339244	0.001727982	0.069823582	2449	2443	1959	1756	1396	1301
Pon3	ENSMUSG00000029759	533.5014818	-0.968221194	0.309015558	-3.133244165	0.001728856	0.069823582	1012	659	447	385	367	331
Rps6ka1	ENSMUSG00000003644	599.8237356	0.604560801	0.193096994	3.130865941	0.001742917	0.070236093	514	486	429	606	752	813
Rbfox3	ENSMUSG00000025576	190.1138685	1.078657383	0.344644214	3.129770757	0.001749428	0.070343177	83	114	169	181	260	333
Mal2	ENSMUSG00000024479	248.5818119	-1.336734407	0.427580655	-3.126274287	0.001770363	0.071028539	297	389	382	242	116	66
Cenpm	ENSMUSG00000068101	24.58861117	1.28683928	0.412172853	3.122086455	0.001795742	0.071741626	14	14	15	32	31	41
Vat1l	ENSMUSG00000046844	84.6656803	-1.141334252	0.365572685	-3.122044667	0.001795997	0.071741626	175	78	97	56	54	48
Gm13889	ENSMUSG00000087006	321.2493408	-1.081680229	0.347053315	-3.116755213	0.001828533	0.072793456	332	331	646	152	199	267
Cdca3	ENSMUSG00000023505	97.80415825	0.990097468	0.317699155	3.116462387	0.00183035	0.072793456	78	57	62	96	118	176
Lrrn3	ENSMUSG00000036295	2654.602278	0.624321934	0.200371107	3.115828139	0.001834291	0.072793456	2123	2428	1715	3561	3268	2831
Sema3b	ENSMUSG00000057969	140.8628266	-0.668449887	0.214734982	-3.112906344	0.001852548	0.073358488	159	177	182	108	121	97
Gm10561	ENSMUSG00000097649	202.6012222	-0.793740801	0.255139721	-3.111004428	0.001864521	0.073660999	215	241	315	160	117	167
Nsg1	ENSMUSG00000029126	227.1705061	-0.760569108	0.244523491	-3.110413254	0.001868258	0.073660999	283	228	347	143	188	175
Gm43858	ENSMUSG00000104546	25.9689143	1.257628255	0.404594721	3.108365454	0.001881253	0.07392814	15	12	19	37	38	35
Sult4a1	ENSMUSG00000018865	35.68150072	-1.094986167	0.352304181	-3.108070318	0.001883133	0.07392814	51	39	55	23	23	22
Sdr9c7	ENSMUSG00000040127	33.01877707	-1.356960253	0.436813634	-3.106497023	0.001893182	0.074163176	58	55	29	24	15	17
Nfkbid	ENSMUSG00000036931	20.15020201	1.418001109	0.456589055	3.105639733	0.001898679	0.074219236	10	11	12	29	24	35
Aspm	ENSMUSG00000033952	71.82439524	1.205365975	0.388443211	3.103068715	0.001915252	0.074707092	55	54	21	85	102	114
Cenpf	ENSMUSG00000026605	173.6806586	0.954891266	0.308312697	3.09715194	0.001953897	0.075921734	157	126	72	216	224	247
Gbp2b	ENSMUSG00000040264	83.05693929	-3.804826358	1.228540856	-3.097028755	0.001954709	0.075921734	171	8	286	5	16	13
Prkar2b	ENSMUSG00000002997	3069.337866	-0.617605383	0.199461575	-3.096362711	0.001959106	0.07593094	3664	4436	3049	2629	2205	2433
Spry2	ENSMUSG00000022114	845.4589748	0.466681344	0.15081185	3.09446071	0.00197171	0.076257574	663	689	777	956	1014	973
Rassf7	ENSMUSG00000038618	142.364921	-0.725097759	0.234398801	-3.093436298	0.00197853	0.076359555	187	201	144	117	104	101
Zbtb42	ENSMUSG00000037638	145.37386	-1.00742618	0.325792779	-3.092229918	0.001986589	0.076440362	179	258	145	128	87	74
A730046J19Rik	ENSMUSG00000085139	45.24189541	-1.340559694	0.433575686	-3.091870089	0.001988999	0.076440362	100	44	50	20	30	27
Tril	ENSMUSG00000043496	586.6500862	-0.7738647	0.250405723	-3.090443338	0.001998579	0.076647197	905	752	564	445	502	352
Rimbp2	ENSMUSG00000029420	19.7610431	-1.576165821	0.510501369	-3.087485984	0.002018573	0.077251674	41	28	20	12	9	9
Twist1	ENSMUSG00000035799	355.4292032	1.38765981	0.44969006	3.085813839	0.002029959	0.077524888	128	127	335	318	475	750
Ildr2	ENSMUSG00000040612	254.9712438	-1.350665547	0.438390651	-3.080963389	0.00206332	0.078587487	489	472	138	169	140	122
Sdf2l1	ENSMUSG00000022769	550.3119691	-0.687412437	0.223148157	-3.080520336	0.002066392	0.078587487	627	786	624	365	379	521
Pim1	ENSMUSG00000024014	742.7542754	0.817899342	0.266090026	3.073769254	0.002113729	0.080220611	433	635	545	1251	756	836
Ankrd9	ENSMUSG00000037904	209.5628369	0.68466335	0.223213241	3.06730617	0.002159975	0.08180569	136	150	197	255	243	278
Gpr22	ENSMUSG00000044067	33.04902264	-1.292446566	0.421588226	-3.065660959	0.002171894	0.082086816	66	34	40	22	16	20
Prrt1	ENSMUSG00000015476	114.6618428	-0.945941996	0.308893738	-3.062354075	0.002196035	0.082827733	129	113	211	72	81	82
Clybl	ENSMUSG00000025545	336.160445	0.528260617	0.172538932	3.061689383	0.002200917	0.082840706	275	280	271	389	363	439
Slc2a1	ENSMUSG00000028645	1081.289691	-0.504800047	0.165137889	-3.056839656	0.002236839	0.084019537	1294	1235	1276	1027	840	816
Arhgef2	ENSMUSG00000028059	528.9286835	-0.558558248	0.182864344	-3.05449513	0.002254397	0.084505159	700	661	528	437	429	418
Col4a6	ENSMUSG00000031273	40.20626289	-1.531630573	0.501719796	-3.052760893	0.002267465	0.084744166	91	59	29	14	28	19
Btnl9	ENSMUSG00000040283	1606.910473	-0.495539984	0.162343422	-3.052418011	0.002270057	0.084744166	1744	2145	1752	1285	1395	1320
Far2	ENSMUSG00000030303	295.7941843	0.940882526	0.30848978	3.049963357	0.002288693	0.085265487	156	158	294	328	385	454
Gm36989	ENSMUSG00000102496	16.95629486	1.548212581	0.508036404	3.047444176	0.002307964	0.085775618	11	7	8	20	27	29
Unc80	ENSMUSG00000055567	59.71456497	-1.388013663	0.455542411	-3.046947178	0.002311783	0.085775618	142	72	45	35	36	28
Clmp	ENSMUSG00000032024	97.52784634	-1.06506613	0.349758989	-3.045142977	0.002325697	0.08611684	105	99	193	52	63	74
Galnt14	ENSMUSG00000024064	13.49060925	-1.815997785	0.596827796	-3.042750014	0.00234427	0.086582311	22	15	26	8	6	4
Aldh18a1	ENSMUSG00000025007	587.8030768	-0.604322749	0.19863989	-3.042303085	0.002347754	0.086582311	746	797	584	417	498	484
Fdx1	ENSMUSG00000032051	5113.002774	0.874723039	0.287617407	3.04127295	0.002355802	0.086601498	2635	4238	3953	6225	4866	8761
Kazald1	ENSMUSG00000025213	183.774416	-1.257552075	0.413532297	-3.041000869	0.002357932	0.086601498	165	232	381	78	81	167
Dlgap1	ENSMUSG00000003279	1546.16042	-0.447076297	0.147044389	-3.040417259	0.002362506	0.086601498	1830	1847	1675	1408	1306	1211
Asns	ENSMUSG00000029752	450.6111834	-1.187409308	0.390683161	-3.039315299	0.002371165	0.086744741	767	828	283	337	241	247
St8sia4	ENSMUSG00000040710	268.384472	-0.579242324	0.190810249	-3.03569817	0.002399795	0.087616502	295	332	338	209	243	194
Agfg2	ENSMUSG00000029722	983.6125644	-0.600507143	0.198007647	-3.03274723	0.002423385	0.088301183	1240	1271	1045	916	657	772
Slc2a4rg-ps	ENSMUSG00000085028	77.19178765	0.982402525	0.324247873	3.02978865	0.002447249	0.088972849	72	40	43	86	104	118
Hmmr	ENSMUSG00000020330	57.40757456	1.057348328	0.34904552	3.029256266	0.002451566	0.088972849	50	33	29	97	63	72
1110032F04Rik	ENSMUSG00000046999	40.30512927	-1.251840918	0.413701994	-3.025948473	0.002478545	0.089773503	66	70	34	30	21	20
Tmprss6	ENSMUSG00000016942	22.37758475	-1.362025936	0.450298471	-3.024718099	0.00248865	0.08996099	36	36	24	13	10	14
Lpar1	ENSMUSG00000038668	527.5438325	-0.565767038	0.18720813	-3.022128568	0.002510039	0.090554871	660	648	581	496	373	408
Fam83d	ENSMUSG00000027654	32.07665123	1.511514486	0.501012888	3.01691737	0.002553594	0.091919492	23	7	20	33	48	62
Lrrtm1	ENSMUSG00000060780	5.846419472	-3.421771147	1.134588012	-3.015871056	0.002562422	0.091919492	10	6	16	2	1	0
Acsl1	ENSMUSG00000018796	1319.33186	0.709370207	0.235217439	3.01580619	0.00256297	0.091919492	990	1218	796	1796	1797	1319
E230013L22Rik	ENSMUSG00000096957	25.04864022	-1.250060195	0.414841242	-3.013345994	0.002583841	0.092279931	33	33	40	14	12	18
Cyp2j9	ENSMUSG00000015224	54.23022972	-0.953127709	0.316332094	-3.013060405	0.002586274	0.092279931	88	62	65	38	31	41
Col6a2	ENSMUSG00000020241	767.5252532	-0.991568487	0.329114631	-3.01283624	0.002588185	0.092279931	814	682	1568	451	564	526
Lum	ENSMUSG00000036446	210.9943999	-0.833355754	0.27687865	-3.009823089	0.002613999	0.09301626	284	262	265	109	139	207
Gpatch3	ENSMUSG00000028850	169.5749758	0.637788202	0.211943337	3.009239223	0.002619028	0.09301626	128	127	143	179	208	232
2810459M11Rik	ENSMUSG00000026227	12.06460264	1.936265173	0.643606823	3.008459672	0.002625756	0.093074145	4	3	8	19	24	14
Tnni3	ENSMUSG00000035458	7.977257011	4.523335521	1.504311416	3.006914308	0.002639141	0.093367302	2	0	0	4	38	4
Nup93	ENSMUSG00000032939	476.9195864	0.522006411	0.173699403	3.00522858	0.002653813	0.093527392	413	366	396	628	551	508
Celf6	ENSMUSG00000032297	24.76278207	-1.31253177	0.436751064	-3.005217111	0.002653913	0.093527392	44	26	36	17	14	13
Rhcg	ENSMUSG00000030549	11.77254666	-1.793198905	0.596894336	-3.004214979	0.002662671	0.093594189	15	22	18	5	5	6
Cdc20	ENSMUSG00000006398	190.2034757	0.651922392	0.217030557	3.003827669	0.002666063	0.093594189	170	139	135	204	232	261
Gm27177	ENSMUSG00000098975	90.35102081	-5.022125889	1.672647338	-3.00250135	0.002677708	0.093611034	257	268	1	11	2	3
Sdr42e1	ENSMUSG00000034308	61.90793019	-0.875935237	0.29176248	-3.00222029	0.002680182	0.093611034	88	83	70	53	39	40
Ankrd35	ENSMUSG00000038354	19.74942335	-1.395849752	0.464983923	-3.001931216	0.002682728	0.093611034	34	23	29	10	13	10
Treml4	ENSMUSG00000051682	35.12342398	1.335779426	0.445046098	3.001440598	0.002687055	0.093611034	22	19	19	30	47	74
Noxo1	ENSMUSG00000019320	78.18778102	0.874434362	0.291658064	2.9981491	0.002716248	0.094447806	49	62	54	114	114	76
Mapk8ip2	ENSMUSG00000022619	54.39455734	-0.97976453	0.327280103	-2.993657479	0.002756552	0.095667032	94	60	63	37	33	40
Ninl	ENSMUSG00000068115	557.8408175	-0.857645236	0.286572018	-2.992773835	0.002764545	0.095762382	998	656	502	429	418	343
Stx1a	ENSMUSG00000007207	94.34764011	-0.895581676	0.299398499	-2.991269757	0.0027782	0.096053092	166	103	99	67	68	63
Bhlhe40	ENSMUSG00000030103	735.1202026	0.916366899	0.30661304	2.988675561	0.002801895	0.096612249	324	573	631	1123	1044	717
Cyp2j6	ENSMUSG00000052914	263.674741	-0.533926104	0.178716984	-2.987550999	0.002812224	0.096612249	287	324	325	221	225	200
Pgbd5	ENSMUSG00000050751	476.5632407	0.480819504	0.160947187	2.987436524	0.002813277	0.096612249	393	382	418	602	545	519
Fam76a	ENSMUSG00000028878	1520.402211	0.547363388	0.183236851	2.987190535	0.002815542	0.096612249	1108	1334	1263	2105	1678	1634
Mgat2	ENSMUSG00000043998	1642.396613	-0.63129504	0.211382314	-2.98650832	0.002821832	0.096646409	1977	2290	1721	1515	1291	1060
Akr1c13	ENSMUSG00000021213	84.44883338	-0.899989526	0.301634748	-2.983706398	0.002847799	0.097198952	132	113	85	56	49	72
Gcnt2	ENSMUSG00000021360	218.0104587	-0.545247054	0.182746884	-2.983618882	0.002848613	0.097198952	252	265	259	191	162	178
Tenm3	ENSMUSG00000031561	145.0311336	1.104273184	0.37018744	2.983007689	0.002854308	0.097211561	116	87	74	197	283	115
Trim7	ENSMUSG00000040350	225.5071834	-0.700945537	0.235077144	-2.981768129	0.00286589	0.097424238	345	266	227	174	156	185
Gm4899	ENSMUSG00000094615	32.43062458	1.243912743	0.417413596	2.980048459	0.002882028	0.097691268	13	29	16	52	40	45
Tmem159	ENSMUSG00000030917	1283.80239	-0.498127347	0.167168532	-2.97979136	0.002884448	0.097691268	1726	1435	1349	1097	1073	1023
Pitpnm3	ENSMUSG00000040543	49.36971533	-1.059586593	0.355805179	-2.977996542	0.002901393	0.098083188	75	81	44	34	33	29
Cdh3	ENSMUSG00000061048	21.82415123	-1.489417623	0.500364561	-2.976664892	0.002914023	0.098328088	27	39	30	6	17	11
Mmp15	ENSMUSG00000031790	279.6662865	-0.743421136	0.249822753	-2.975794344	0.002922308	0.0983629	400	378	272	229	233	166
Gng4	ENSMUSG00000021303	31.11410474	-1.291812087	0.434160568	-2.975424718	0.002925832	0.0983629	54	42	36	13	14	27
Pik3c2b	ENSMUSG00000026447	330.134451	0.658246991	0.221380046	2.973379961	0.002945396	0.098838607	322	222	225	385	433	394
Ptpru	ENSMUSG00000028909	732.6128216	-1.041269983	0.350356576	-2.972029221	0.002958385	0.099042499	1195	1241	522	504	564	369
Scd2	ENSMUSG00000025203	21407.05107	-0.696301502	0.234317076	-2.971620822	0.002962323	0.099042499	29869	29704	19852	19232	15819	13966
Fam110c	ENSMUSG00000036136	9.603256643	-2.117244139	0.712882095	-2.969978003	0.002978211	0.099289743	18	14	15	1	5	5
Slc38a1	ENSMUSG00000023169	165.47911	-0.591283821	0.199173534	-2.968686694	0.002990754	0.099289743	205	182	210	122	140	134
1700086L19Rik	ENSMUSG00000071265	8.626507713	-2.482367296	0.836238906	-2.968490557	0.002992663	0.099289743	22	9	13	1	2	5
Rph3a	ENSMUSG00000029608	16.0611823	-1.873970655	0.631311989	-2.968374889	0.002993789	0.099289743	40	22	14	4	10	7
Ppfibp1	ENSMUSG00000016487	2673.572631	-1.02135018	0.344114376	-2.968054375	0.002996913	0.099289743	4074	4460	2213	2329	1816	1150
Galnt16	ENSMUSG00000021130	145.1446091	-0.749643186	0.252660062	-2.967003095	0.003007179	0.099449381	212	194	140	97	120	107
Opn3	ENSMUSG00000026525	362.8483728	0.832572359	0.280686251	2.966202849	0.003015016	0.099528227	301	298	185	377	434	583

Table S5. Genes differentially expressed in ABC Male vs ABC Female

								Read count						
Gene	ENSEMBL_Gene_ID	baseMean	log2FoldChange	lfcSE	stat	pvalue	padj	166836_No	166838_No	182098_Tu	184208_Tu	183159_B2	BS05_Norm	207386_No
Ddx3y	ENSMUSG00000069045	1046.848744	12.23680254	1.023642768	11.95417281	6.17453E-33	3.9801E-29	2451	2744	2130	1	0	0	1
Eif2s3y	ENSMUSG00000069049	398.4463562	11.55675408	1.067877975	10.82216728	2.70309E-27	1.30681E-23	818	866	1105	0	0	1	0
Drd4	ENSMUSG00000025496	54.58397991	7.336438255	1.064060904	6.894754078	5.39581E-12	6.13789E-09	50	79	249	1	3	0	0
Gm29650	ENSMUSG00000099876	11.55833114	7.170987222	1.187473369	6.038861511	1.55205E-09	1.00045E-06	22	24	35	0	0	0	0
Susd3	ENSMUSG00000021384	769.1433918	4.634436514	0.556861731	8.322418751	8.61862E-17	1.51515E-13	1322	1717	2068	45	158	43	30
Kdm5d	ENSMUSG00000056673	284.7519157	4.423946895	0.33653877	13.14543014	1.80775E-39	3.49582E-35	767	608	500	21	41	29	26
Uty	ENSMUSG00000068457	338.1005811	4.15321738	0.832168958	4.990834301	6.01191E-07	0.000181653	908	723	570	14	132	11	10
4930438A08Rik	ENSMUSG00000069873	12.3566045	3.727209382	0.757595106	4.919790735	8.66368E-07	0.00024638	27	34	17	4	1	2	1
Aldh1l2	ENSMUSG00000020256	51.41029936	3.627532669	0.743368908	4.879855252	1.06164E-06	0.000293285	161	151	14	9	5	11	10
Cox6a2	ENSMUSG00000030785	33.40694447	3.256909082	0.651811756	4.996701961	5.83191E-07	0.000179012	66	104	34	4	16	7	3
Cacnb4	ENSMUSG00000017412	26.70626031	3.186838369	0.579601553	5.498326143	3.83413E-08	1.75172E-05	47	74	41	6	12	5	2
Tnn	ENSMUSG00000026725	50.75434258	3.168358092	0.747366309	4.239364355	2.24154E-05	0.004291764	73	162	75	8	8	28	1
Lcn2	ENSMUSG00000026822	15.93920059	3.068115533	0.730132013	4.202138078	2.64406E-05	0.004734332	35	43	18	0	5	5	5
Paqr5	ENSMUSG00000032278	150.0284161	2.921961595	0.507390439	5.758803027	8.47125E-09	4.55047E-06	259	329	304	64	55	26	13
Trib3	ENSMUSG00000032715	153.4450361	2.913239444	0.441426426	6.599603637	4.12258E-11	4.19592E-08	365	410	138	38	31	42	51
Kcnk1	ENSMUSG00000033998	669.6760387	2.848949389	0.304245709	9.363975577	7.67916E-21	1.65E-17	1204	1377	1374	236	211	168	118
4632428C04Rik	ENSMUSG00000097184	14.0534001	2.797561128	0.617143972	4.533076968	5.81306E-06	0.001405162	19	35	28	3	4	6	3
Bhlha15	ENSMUSG00000052271	58.72151174	2.704404621	0.581638533	4.649631119	3.32529E-06	0.000835123	129	169	44	11	9	20	29
Gsta3	ENSMUSG00000025934	329.2782076	2.684706562	0.347617508	7.723162681	1.13478E-14	1.68803E-11	652	613	643	84	160	84	69
1520401A03Rik	ENSMUSG00000043747	7.517464613	2.563030032	0.783517234	3.271185265	0.001070977	0.073950605	12	16	16	4	0	2	3
Adcy8	ENSMUSG00000022376	70.49321408	2.505202727	0.408562646	6.131746878	8.69193E-10	6.22535E-07	141	170	90	24	13	31	24
9330159F19Rik	ENSMUSG00000004360	266.5886506	2.482105194	0.553758741	4.48228626	7.38476E-06	0.001704018	544	430	534	154	48	128	28
Gdf15	ENSMUSG00000038508	60.50482018	2.399121087	0.446222178	5.376516914	7.59406E-08	3.05946E-05	124	154	60	27	24	19	16
Adamts8	ENSMUSG00000031994	31.72002932	2.396547243	0.642801487	3.728285156	0.000192787	0.022458543	38	84	56	8	1	16	19
Tubb3	ENSMUSG00000062380	451.4934974	2.321252971	0.439060826	5.286859663	1.24434E-07	4.62751E-05	508	742	1246	212	82	192	179
Pdgfrl	ENSMUSG00000031595	77.37931562	2.227076981	0.297367842	7.489300007	6.92419E-14	8.92667E-11	135	138	148	26	33	34	26
Derl3	ENSMUSG00000009092	49.72859925	2.06477907	0.589372236	3.503353136	0.00045944	0.043150363	87	130	48	18	8	42	16
Foxo6	ENSMUSG00000052135	36.36172752	1.968767633	0.619080795	3.180146516	0.001472006	0.092121855	27	51	112	15	8	13	29
5031414D18Rik	ENSMUSG00000034959	45.52840953	1.917700445	0.368056594	5.210341232	1.88494E-07	6.62744E-05	70	66	100	21	16	22	23
Grin2d	ENSMUSG00000002771	47.85536355	1.915363823	0.559583	3.422841332	0.000619702	0.0528984	36	64	148	31	15	23	18
Prss35	ENSMUSG00000033491	7597.575661	1.830608125	0.295724572	6.190246935	6.007E-10	4.68414E-07	12741	14450	11492	4003	2658	2969	4871
Ampd3	ENSMUSG00000005686	342.1663572	1.757962229	0.409125643	4.296876176	1.73222E-05	0.003453366	536	485	698	196	71	211	198
Glt8d2	ENSMUSG00000020251	151.4439101	1.754613768	0.400864129	4.377078521	1.20281E-05	0.00254497	200	363	198	58	51	109	82
Tsku	ENSMUSG00000049580	291.3594715	1.747530891	0.408062399	4.282508992	1.84798E-05	0.00363298	451	535	473	70	215	182	113
Fgf11	ENSMUSG00000042826	270.3718814	1.727687771	0.323180877	5.345884901	8.99764E-08	3.47993E-05	364	478	508	197	103	119	124
Acan	ENSMUSG00000030607	2627.371611	1.69465377	0.439611351	3.854890841	0.000115781	0.0150267	4123	5170	3733	2343	632	1081	1308
Prrg3	ENSMUSG00000033361	3226.380308	1.64245984	0.508820794	3.227973107	0.001246707	0.08172481	6570	7038	2218	1914	722	2371	1751
Jam2	ENSMUSG00000053062	815.5511485	1.63592875	0.271365031	6.028517166	1.65471E-09	1.03222E-06	1244	1671	1080	489	356	479	389
Chac1	ENSMUSG00000027313	66.34990642	1.626814943	0.39461149	4.122573682	3.74663E-05	0.006245886	125	120	80	37	20	33	50
Hhex	ENSMUSG00000024986	910.2269489	1.544843719	0.384229252	4.020630164	5.80427E-05	0.008701001	1286	1424	1663	258	456	521	764
Zbtb16	ENSMUSG00000066687	114.7796148	1.538822023	0.351868901	4.373282263	1.22392E-05	0.00254497	256	156	140	72	59	74	48
Speg	ENSMUSG00000026207	184.8438817	1.533969187	0.249712489	6.142941402	8.10072E-10	6.02506E-07	290	311	284	93	127	101	88
Pdp1	ENSMUSG00000049225	2641.631006	1.507077469	0.225806227	6.67420687	2.48573E-11	2.6705E-08	4488	4097	4004	1526	1263	1795	1318
Zfp69	ENSMUSG00000064141	39.83588766	1.475748177	0.431881935	3.417017608	0.000633112	0.053697881	46	49	94	25	16	25	24
Shh	ENSMUSG00000002633	7053.141673	1.468141294	0.412956635	3.555194833	0.000377699	0.038030607	7996	8717	16604	6033	2429	4147	3446
Ppp1r3b	ENSMUSG00000046794	125.275267	1.452688477	0.354097683	4.102507713	4.08696E-05	0.006687622	216	236	139	70	51	66	100
Pitpnm3	ENSMUSG00000040543	41.22092005	1.41782772	0.433615105	3.269783969	0.001076296	0.073950605	72	78	43	33	17	28	17
Penk	ENSMUSG00000045573	124.4538138	1.399609794	0.341292807	4.100906212	4.11535E-05	0.006687622	220	146	211	50	90	64	90
Stard5	ENSMUSG00000046027	325.4734983	1.391621343	0.244508343	5.691508614	1.25922E-08	6.40809E-06	474	437	599	170	207	179	212
Map2	ENSMUSG00000015222	423.8694955	1.38128227	0.436264312	3.166159212	0.001544662	0.09482755	743	806	415	338	112	310	244
Fads3	ENSMUSG00000024664	266.2092801	1.366824328	0.415621029	3.288631308	0.001006758	0.07061468	430	522	276	140	95	141	260
Amotl1	ENSMUSG00000013076	3306.572111	1.298425647	0.392591964	3.30731591	0.000941946	0.068222291	5718	6185	3106	2363	2778	1813	1182
Itgb8	ENSMUSG00000025321	361.579721	1.288411095	0.376475816	3.422294444	0.00062095	0.0528984	643	561	434	300	127	288	178
Mmp24	ENSMUSG00000027612	114.3352986	1.286583568	0.346668059	3.71128385	0.000206211	0.023595874	177	230	111	68	72	83	60
Tro	ENSMUSG00000025272	192.9562521	1.280605083	0.364774934	3.510671822	0.000446976	0.042579403	374	332	165	111	152	105	111
Hivep3	ENSMUSG00000028634	271.0569464	1.274498075	0.30313384	4.204407119	2.61767E-05	0.004734332	447	444	332	170	231	141	132
Htr3a	ENSMUSG00000032269	229.0798043	1.271010657	0.374596358	3.393013921	0.000691281	0.056885093	286	353	395	114	131	230	95
Kcnc3	ENSMUSG00000062785	36.3853384	1.269830853	0.382055788	3.323679144	0.000888383	0.065570835	49	64	50	17	31	24	20
Cers6	ENSMUSG00000027035	81.4612258	1.264629263	0.386108575	3.275320326	0.001055422	0.073681429	107	181	79	54	55	55	40
Slc4a11	ENSMUSG00000074796	88.99215241	1.249593755	0.299660053	4.170037827	3.04549E-05	0.005403092	103	166	130	54	57	56	57
Maneal	ENSMUSG00000042763	266.7406834	1.246401434	0.361526378	3.447608555	0.000565573	0.049940877	394	473	329	128	112	178	253
Smarca1	ENSMUSG00000031099	1112.89876	1.216569304	0.313872628	3.875996808	0.000106189	0.013969281	2050	1826	1074	834	721	731	554
Lifr	ENSMUSG00000054263	1052.650117	1.194167183	0.326104203	3.661919012	0.000250333	0.027355237	1753	1857	1046	864	488	648	712
Adamtsl4	ENSMUSG00000015850	100.5890668	1.182641227	0.351251656	3.366934241	0.000760088	0.059750341	129	163	152	80	36	65	79
Mpp3	ENSMUSG00000052373	103.8797507	1.171005225	0.282430885	4.146165619	3.38089E-05	0.005837474	152	139	166	68	67	83	53
Atf5	ENSMUSG00000038539	1329.517493	1.161331304	0.335401354	3.46251227	0.000535158	0.048359234	1931	2566	1333	685	1112	698	981
2010007H06Rik	ENSMUSG00000044694	311.9267151	1.160570496	0.291381246	3.982996546	6.80517E-05	0.009894621	425	520	422	267	207	146	196
Tgfbr3	ENSMUSG00000029287	3861.464092	1.139053317	0.313332892	3.635281662	0.000277677	0.029503953	6024	6876	3938	2780	1797	3059	2556
Eda	ENSMUSG00000059327	91.92443301	1.12079511	0.331884047	3.377068342	0.000732628	0.058302753	133	154	113	64	45	83	51
Asph	ENSMUSG00000028207	2467.872162	1.117847913	0.265914007	4.203794771	2.62477E-05	0.004734332	3663	4084	2955	1711	1198	1889	1775
Klf15	ENSMUSG00000030087	402.1668676	1.097252478	0.273894973	4.006106669	6.17278E-05	0.009112151	481	515	738	326	246	242	267
Rtn4rl1	ENSMUSG00000045287	4352.878817	1.080675047	0.212205066	5.092597763	3.5319E-07	0.000119825	6183	5897	6609	3425	3077	2478	2801
Ccdc85a	ENSMUSG00000032878	130.1997464	1.072835974	0.326255731	3.288328363	0.001007842	0.07061468	213	212	134	78	80	118	77
Acot2	ENSMUSG00000021226	232.4384155	1.051878735	0.246218684	4.272132062	1.93613E-05	0.003744085	299	330	361	128	162	183	164
Tmem117	ENSMUSG00000063296	109.2634736	1.031001456	0.288631017	3.57203972	0.000354212	0.036242039	135	180	148	88	57	74	82
Ncam1	ENSMUSG00000039542	228.7391812	0.953523141	0.267049502	3.570585737	0.000356184	0.036252017	308	274	366	169	202	148	135
Fli1	ENSMUSG00000016087	298.8886766	0.902975851	0.284466697	3.174276149	0.001502107	0.093101751	397	359	466	297	188	192	193
Apln	ENSMUSG00000037010	959.2443143	0.894200741	0.237959168	3.757790663	0.00017142	0.02048155	1243	1335	1332	749	583	860	611
Bbs4	ENSMUSG00000025235	477.9673231	0.870579947	0.238111301	3.65618912	0.000255993	0.027655774	575	575	785	387	356	314	355
Sema3b	ENSMUSG00000057969	123.7154014	0.868926399	0.261258471	3.325926223	0.000881253	0.065570835	153	172	176	72	98	101	95
Scml4	ENSMUSG00000044770	234.6942703	0.864017509	0.246634995	3.503223496	0.000459664	0.043150363	354	346	248	165	184	174	172
Cd200	ENSMUSG00000022661	1072.480204	0.863217744	0.262105876	3.293393332	0.000989859	0.07011682	1314	1677	1342	683	635	929	928
Trib2	ENSMUSG00000020601	845.1608904	0.842354068	0.259358472	3.247837096	0.001162858	0.078081088	1113	1218	1064	751	455	701	616
Bcl2	ENSMUSG00000057329	404.2529121	0.83822343	0.234870789	3.568870501	0.000358524	0.036299104	587	513	522	375	275	276	283
Fhl1	ENSMUSG00000023092	853.1120659	0.819485431	0.230587431	3.553903292	0.000379559	0.038030607	1050	1083	1269	641	580	780	568
Sgsm3	ENSMUSG00000042303_I	453.5715607	0.814441013	0.24102513	3.379070942	0.000727312	0.058118871	572	559	676	404	283	368	313
Cacna2d1	ENSMUSG00000040118	6202.943495	0.802313133	0.247438465	3.242475381	0.001184962	0.078744991	9109	8599	6900	5545	3790	5159	4318
Jag1	ENSMUSG00000027276	299.0829684	0.776399329	0.239908484	3.236231228	0.001211192	0.079779168	406	424	348	265	186	232	232
Slc29a1	ENSMUSG00000023942	2491.608698	0.718985561	0.216815981	3.31610962	0.0009128	0.066731232	3132	3804	2701	1955	1825	1934	2091
Arl15	ENSMUSG00000042348	1331.015427	0.611175269	0.173725594	3.518049673	0.000434731	0.042034138	1629	1703	1643	1139	1094	1105	1005
Eif2s3x	ENSMUSG00000035150	4981.069602	-0.67314522	0.175830364	-3.828378695	0.00012899	0.016151143	3796	3848	3510	6299	6091	5890	5434
Gm8203	ENSMUSG00000101878	574.8860218	-0.713205365	0.201692114	-3.536109324	0.000406067	0.039659173	434	397	432	677	640	787	656
Adck3	ENSMUSG00000026489	2184.2285	-0.713463334	0.206902452	-3.448307779	0.000564111	0.049940877	1819	1516	1463	2447	2986	2555	2503
Rps6ka1	ENSMUSG00000003644	627.7080496	-0.71669517	0.22113531	-3.24098024	0.001191194	0.078888073	495	471	412	712	647	858	799
Rasal1	ENSMUSG00000029602	259.9515606	-0.719542972	0.220062816	-3.269716285	0.001076554	0.073950605	189	174	208	313	286	349	302
Sccpdh	ENSMUSG00000038936	1892.938913	-0.831214794	0.216187171	-3.844884923	0.000120609	0.015548916	1273	1312	1343	2558	2663	1941	2160
Rangap1	ENSMUSG00000022391	1546.169035	-0.850324932	0.266008178	-3.196611995	0.001390518	0.088163422	1030	1258	891	1811	2464	1607	1762
Dusp14	ENSMUSG00000018648	227.9072555	-0.858298764	0.26412079	-3.249644839	0.001155492	0.077856822	129	149	189	267	251	274	336
Echdc2	ENSMUSG00000028601	267.7420314	-0.859368973	0.254073956	-3.382357595	0.000718665	0.057972352	175	155	218	340	380	325	281
Pkdcc	ENSMUSG00000024247	1252.370834	-0.889666964	0.276331738	-3.219561276	0.001283869	0.083172378	875	737	914	1525	1311	1287	2117
Plin2	ENSMUSG00000028494	4402.383981	-0.934264081	0.288030992	-3.243623455	0.001180197	0.078744991	2625	2656	3404	3715	6339	5539	6538
Nmnat3	ENSMUSG00000032456	429.3325543	-0.941006797	0.253276666	-3.715331589	0.000202937	0.023359543	313	270	261	599	632	420	510

Table S5. Genes differentially expressed in ABC Male vs ABC Female

								Read count						
Gene	ENSEMBL_Gene_ID	baseMean	log2FoldChange	lfcSE	stat	pvalue	padj	166836_No	166838_No	182098_Tu	184208_Tu	183159_B2	BS05_Norm	207386_No
5930412G12Rik	ENSMUSG00000072591	319.1366747	-0.949088398	0.286609444	-3.311434496	0.00092819	0.067478691	214	169	242	491	434	383	300
Opn3	ENSMUSG00000026525	386.3840882	-0.953362147	0.281341218	-3.388633039	0.000702419	0.05731387	290	288	177	532	409	527	481
Shtn1	ENSMUSG00000041362	405.8820789	-0.958357908	0.222406826	-4.309031009	1.63971E-05	0.003337767	285	281	226	550	455	497	548
Spint2	ENSMUSG00000074227	2218.808353	-0.963194831	0.286503753	-3.361892543	0.000774102	0.06036126	1110	1408	1797	2324	3493	2416	2984
Fam110b	ENSMUSG00000049119	173.9652517	-0.963408713	0.251511173	-3.830480781	0.000127893	0.016151143	120	115	104	253	203	237	185
Tacc3	ENSMUSG00000037313	163.9177864	-0.975009148	0.303239179	-3.215313907	0.001303019	0.083713591	121	103	92	238	253	149	192
Zfp385a	ENSMUSG00000000552	730.6092465	-0.976267955	0.217829809	-4.481792272	7.40188E-06	0.001704018	448	452	510	936	1075	787	906
Skap1	ENSMUSG00000057058	558.7793622	-0.998871677	0.255905379	-3.903285197	9.48958E-05	0.012743711	363	377	327	663	918	576	688
Mttp	ENSMUSG00000028158	137.0157084	-0.999828146	0.262945066	-3.802422163	0.000143288	0.017762237	98	84	80	205	159	153	180
Actg1	ENSMUSG00000062825	21639.2174	-1.002721997	0.211796739	-4.734359932	2.19747E-06	0.000566597	12506	15143	13605	23615	31944	27382	27280
Cdca7	ENSMUSG00000055612	69.90376322	-1.039467943	0.312609392	-3.325133437	0.000883762	0.065570835	45	38	48	114	84	81	79
Cadm1	ENSMUSG00000032076	1698.050422	-1.066350123	0.265536171	-4.015837542	5.92351E-05	0.008811442	1069	976	1089	2882	2063	2156	1651
Ndrg2	ENSMUSG00000004558	2671.181103	-1.082476522	0.336268151	-3.219087264	0.001285993	0.083172378	1528	1378	1983	2465	5210	3035	3099
Runx2	ENSMUSG00000039153	115.684876	-1.095545245	0.32316989	-3.389997885	0.000698932	0.057270932	75	58	77	122	210	137	131
Mocos	ENSMUSG00000039616	453.1324143	-1.100357891	0.326652696	-3.368586592	0.000755547	0.059635756	338	242	241	638	782	545	385
Hmgn2	ENSMUSG00000003038	1120.513022	-1.120323826	0.342979646	-3.266444054	0.001089073	0.074418723	578	496	937	1133	1882	1172	1644
Cd24a	ENSMUSG00000047139	1975.508646	-1.121037464	0.336667649	-3.329804529	0.00086907	0.065139807	1293	1291	962	3213	2571	3023	1476
6030419C18Rik	ENSMUSG00000066607	146.0549978	-1.125807903	0.32977766	-3.413839205	0.000640544	0.054090976	77	73	111	167	250	141	203
Slain1	ENSMUSG00000055717	213.2783737	-1.155338692	0.294941675	-3.917176823	8.9592E-05	0.012287452	138	97	142	291	211	343	271
Tubb6	ENSMUSG00000001473	1171.653918	-1.18940555	0.373297306	-3.186215194	0.001441473	0.090798735	807	667	555	1317	1723	870	2262
Sema5a	ENSMUSG00000022231	1014.235419	-1.197500495	0.345278909	-3.468212111	0.000523934	0.047791638	697	535	519	1605	901	1848	996
Ica1l	ENSMUSG00000026018	32.28923334	-1.19817087	0.36946706	-3.242970751	0.001182904	0.078744991	19	17	19	44	36	44	46
Cspg5	ENSMUSG00000032482	1469.352261	-1.241490791	0.34377026	-3.611396725	0.000304552	0.031494297	863	767	846	1795	3099	1450	1466
Dusp5	ENSMUSG00000034765	137.466031	-1.249090096	0.314010849	-3.9778565	6.95393E-05	0.009961121	72	83	76	124	172	220	215
Lipa	ENSMUSG00000024781	3820.166664	-1.251554921	0.345312941	-3.624407817	0.000289624	0.030218402	2240	2526	1641	3818	3493	6841	6183
Chaf1b	ENSMUSG00000022945	76.68935586	-1.255356325	0.390770875	-3.212512513	0.001315794	0.084254396	51	30	48	119	118	61	111
Neto2	ENSMUSG00000036902	152.7197719	-1.26286823	0.396154234	-3.187819594	0.001433499	0.090591543	79	85	91	266	98	255	195
Lrrn1	ENSMUSG00000034648	1114.383769	-1.272941481	0.386146497	-3.296524745	0.00097889	0.070062954	787	680	381	1699	844	1849	1561
Itm2a	ENSMUSG00000031239	105.0650611	-1.282452218	0.386793092	-3.315602693	0.000914457	0.066731232	70	52	50	79	178	177	129
Fbln1	ENSMUSG00000006369	4019.559425	-1.299757973	0.307524869	-4.226513376	2.3734E-05	0.00449969	2630	2294	1647	7133	4147	5746	4541
Cenpa	ENSMUSG00000029177	103.0741877	-1.329454857	0.390886446	-3.401128052	0.000671084	0.055937152	61	60	45	137	206	81	132
Pak1	ENSMUSG00000030774	407.0365893	-1.329947739	0.26779558	-4.966279655	6.82495E-07	0.000203048	233	218	205	699	437	586	471
Rad51	ENSMUSG00000027323	60.49966207	-1.353393813	0.426117919	-3.176101621	0.001492687	0.093101751	29	38	29	131	70	52	74
Man1a	ENSMUSG00000003746	500.0094602	-1.359843321	0.405635266	-3.352379427	0.000801201	0.061974524	285	281	226	345	584	689	1090
Tmem132a	ENSMUSG00000024736	514.7244057	-1.388574292	0.402652273	-3.448569359	0.000563565	0.049940877	303	288	210	389	1036	510	866
Dlgap5	ENSMUSG00000037544	47.93097834	-1.398117705	0.415800112	-3.36247554	0.00077247	0.06036126	30	24	20	79	86	40	57
Pla2g2f	ENSMUSG00000028749	528.5872363	-1.41696053	0.407219338	-3.479600298	0.000502162	0.04624198	259	384	169	589	886	967	446
Cdkn1c	ENSMUSG00000037664	7443.987207	-1.417597792	0.440767791	-3.21620096	0.001298998	0.083713591	2778	3558	5086	5146	14666	7090	13783
Adam5	ENSMUSG00000031554	29.66136183	-1.419464127	0.430848856	-3.294575596	0.000985704	0.070079225	20	12	13	35	53	34	40
Efna5	ENSMUSG00000048915	221.2697193	-1.424518045	0.364232825	-3.911009514	9.19112E-05	0.012516745	83	108	147	333	416	261	201
Gnai1	ENSMUSG00000057614	552.6832287	-1.424865593	0.332828563	-4.281079665	1.85989E-05	0.00363298	385	299	161	757	773	767	728
Rbm24	ENSMUSG00000038132	4565.054604	-1.443616081	0.329674828	-4.378909033	1.19275E-05	0.00254497	2186	2554	2167	8063	3793	7778	5414
2810417H13Rik	ENSMUSG00000040204	70.81637147	-1.444038613	0.427159664	-3.380559387	0.000723384	0.058044845	35	33	39	113	123	45	108
Ccne1	ENSMUSG00000002068	54.58368896	-1.444661106	0.356068352	-4.057257818	4.96523E-05	0.00774335	30	31	22	96	59	67	77
Ube2c	ENSMUSG00000001403	116.2533301	-1.44641318	0.45775553	-3.159794009	0.001578807	0.096617004	52	49	75	179	227	65	166
Car8	ENSMUSG00000041261	68.16964762	-1.446473761	0.347246669	-4.165551153	3.10601E-05	0.00546037	32	31	41	80	78	90	127
Prkcz	ENSMUSG00000029053	170.1708166	-1.44975277	0.408209074	-3.551495696	0.000383048	0.038182408	109	88	60	328	238	245	123
Pid1	ENSMUSG00000045658	3544.42227	-1.452348997	0.403025026	-3.603619884	0.000313816	0.032279638	2099	1483	1755	5623	2089	5834	5927
Rsph1	ENSMUSG00000024033	128.3162066	-1.457102518	0.401957274	-3.625018412	0.000288941	0.030218402	84	44	65	111	175	158	262
Parpbp	ENSMUSG00000035365	35.90603645	-1.461106099	0.439483727	-3.324596585	0.000885466	0.065570835	19	17	17	77	44	38	38
Cdk1	ENSMUSG00000019942	176.8608331	-1.468324943	0.391010213	-3.755208675	0.000173197	0.020547766	90	76	97	312	277	118	268
Etv4	ENSMUSG00000017724	162.6402483	-1.468713891	0.387045466	-3.794680518	0.000147834	0.018208968	90	81	72	224	348	195	129
Rbfox3	ENSMUSG00000025576	237.3503219	-1.470473288	0.439380745	-3.346694879	0.000817812	0.062509262	80	111	163	312	549	205	242
Cdca8	ENSMUSG00000028873	120.2738062	-1.530180785	0.406273946	-3.766376848	0.000165634	0.020018906	62	44	68	220	194	85	170
Acot7	ENSMUSG00000028937	937.6900933	-1.534985662	0.377414133	-4.0671123	4.75993E-05	0.00748354	448	509	393	1154	2168	853	1039
Tk1	ENSMUSG00000025574	92.48772311	-1.538418464	0.438875773	-3.505362019	0.000455987	0.043150363	59	30	45	179	95	77	164
Lars2	ENSMUSG00000035202	15574.33999	-1.554442719	0.493167647	-3.151955991	0.001621807	0.098315068	7448	7564	7162	8979	41139	13595	23133
Gm37691	ENSMUSG00000104348	30.33635946	-1.556370042	0.451669116	-3.445819044	0.000569331	0.049942759	16	17	10	57	39	43	30
Gm42528	ENSMUSG00000107134	118.0272115	-1.562462007	0.348231952	-4.486842749	7.22864E-06	0.001704018	75	55	37	171	146	137	206
Kif23	ENSMUSG00000032254	100.3888433	-1.57300211	0.477292727	-3.295675845	0.000981852	0.070062954	64	41	36	231	156	72	102
Kif2c	ENSMUSG00000028678	48.64894974	-1.582422605	0.466082862	-3.395152953	0.000685903	0.056683703	27	29	13	99	59	43	72
Pbk	ENSMUSG00000022033	136.8594079	-1.587127904	0.424788957	-3.736273924	0.000186767	0.021889117	87	55	49	303	148	118	197
Hs3st1	ENSMUSG00000051022	804.1151066	-1.591384347	0.263078632	-6.049082483	1.45673E-09	9.71388E-07	430	342	350	1068	870	1160	1408
Clstn3	ENSMUSG00000008153	214.2683954	-1.593169908	0.39093414	-4.075289784	4.59571E-05	0.007284574	120	106	73	305	174	446	275
Hs6st2	ENSMUSG00000062184	274.8881146	-1.605418655	0.366840354	-4.376341471	1.20688E-05	0.00254497	144	115	121	426	579	253	287
Cdca3	ENSMUSG00000023505	137.5048421	-1.609150362	0.374109414	-4.301282731	1.69812E-05	0.003420655	75	55	59	210	270	113	180
Melk	ENSMUSG00000035683	52.42440554	-1.619135968	0.510059431	-3.174406489	0.001501433	0.093101751	29	31	12	117	61	42	75
Csrp2	ENSMUSG00000020186	1467.355439	-1.619206593	0.372176539	-4.35064122	1.3574E-05	0.002792489	673	592	751	1544	3218	1269	2226
Lrrc2	ENSMUSG00000032495	117.2170761	-1.63081329	0.403977271	-4.036893675	5.41636E-05	0.008312823	50	62	48	92	253	147	170
Aurkb	ENSMUSG00000020897	78.46910206	-1.635049103	0.405570846	-4.031475925	5.54277E-05	0.008439846	43	31	33	167	104	68	104
Cd1d2	ENSMUSG00000041750	92.56257728	-1.639432081	0.501473158	-3.269231972	0.001078399	0.073950605	48	61	16	94	102	122	204
Spc25	ENSMUSG00000005233	65.57879736	-1.645839432	0.498801678	-3.299586797	0.000968273	0.069607656	34	28	27	121	134	35	81
Bmp7	ENSMUSG00000008999	234.6443484	-1.646713201	0.483803717	-3.403680343	0.000664845	0.055899018	84	127	107	114	325	388	499
Gm10516	ENSMUSG00000097316	36.98695672	-1.650162058	0.415939162	-3.967315916	7.26866E-05	0.010259955	19	14	16	61	36	45	67
Sema4f	ENSMUSG00000000627	19.92794542	-1.652579669	0.515397149	-3.206419888	0.001343977	0.085775021	10	8	10	28	29	38	17
Nudt14	ENSMUSG00000002804	591.7297412	-1.655822726	0.469968976	-3.523259644	0.000426274	0.041423506	226	207	363	465	1567	597	717
Cck	ENSMUSG00000032532	41.67739397	-1.662715856	0.500468733	-3.32231715	0.000892732	0.065641231	21	23	12	62	61	28	85
Gm3912	ENSMUSG00000092178	74.97512627	-1.663201068	0.473077849	-3.515702697	0.000438592	0.042196461	19	25	56	91	159	93	82
Tmem255a	ENSMUSG00000036502	1414.368764	-1.663203356	0.428126174	-3.884843905	0.000102396	0.01365605	763	606	527	1969	817	2429	2790
Knstrn	ENSMUSG00000027331	121.407035	-1.665380564	0.400134096	-4.162056116	3.15395E-05	0.005494689	68	58	36	249	186	116	137
Ccna2	ENSMUSG00000027715	203.0074073	-1.675029919	0.422771358	-3.962023181	7.43173E-05	0.010414119	117	83	70	459	254	156	282
Rgs7	ENSMUSG00000026527	29.38685549	-1.705022446	0.508918487	-3.350285926	0.000807282	0.062156272	14	13	11	31	29	34	72
Tnc	ENSMUSG00000028364	3133.632714	-1.724715558	0.343030942	-5.027871675	4.95954E-07	0.000157225	1362	1365	1330	5190	4019	6149	2520
Shcbp1	ENSMUSG00000022322	49.78271366	-1.725219618	0.495067602	-3.48481624	0.000492475	0.045566909	33	21	11	105	74	46	59
Stmn1	ENSMUSG00000028832	927.4942236	-1.731062993	0.528882904	-3.273055302	0.001063917	0.073950605	362	346	489	1269	2615	465	947
Necab3	ENSMUSG00000027489	24.12945188	-1.738067815	0.51391029	-3.382045173	0.000719483	0.057972352	9	8	15	22	51	34	31
Atp1a3	ENSMUSG00000040907	1273.91027	-1.740269046	0.521734834	-3.335543138	0.000851329	0.064560819	582	639	414	898	857	2005	3523
Snap91	ENSMUSG00000033419	273.7113154	-1.755340701	0.42494578	-4.130740399	3.61597E-05	0.006133823	150	106	92	192	366	403	606
Ccdc80	ENSMUSG00000022665	1114.439284	-1.762420323	0.476761261	-3.696651695	0.000218462	0.024850666	597	477	338	2723	1008	758	1900
Pdzrn4	ENSMUSG00000036218	66.03969638	-1.767586196	0.514084177	-3.438320564	0.000585334	0.05082786	25	34	24	166	64	105	43
Pttg1	ENSMUSG00000020415	333.3383901	-1.790248927	0.56823111	-3.15056479	0.001629551	0.098475809	105	105	206	265	1028	225	400
Cd44	ENSMUSG00000005087	606.289961	-1.792249411	0.395959063	-4.526350264	6.00111E-06	0.001432709	258	252	245	535	865	620	1469
Dhcr24	ENSMUSG00000034926	1810.402437	-1.7959931	0.451997431	-3.973458646	7.08365E-05	0.01007232	1019	959	272	2630	2024	2756	3012
Ccnb2	ENSMUSG00000032218	128.0470097	-1.799151485	0.388698524	-4.628655301	3.68048E-06	0.000912475	50	54	54	256	217	101	164
Gm15662	ENSMUSG00000089855	2051.555146	-1.811671052	0.522690821	-3.466047191	0.000528171	0.047951936	791	863	874	1025	5632	1892	3284
Adh6-ps1	ENSMUSG00000090306	43.93793454	-1.813313537	0.533059713	-3.401708088	0.000669661	0.055937152	13	10	31	38	64	54	97
Rpp25	ENSMUSG00000062309	43.26525699	-1.81818372	0.525721182	-3.458456275	0.00054328	0.048864918	10	22	21	34	104	64	48
Cdca2	ENSMUSG00000048922	50.64718766	-1.835622145	0.475937056	-3.856859056	0.000114853	0.015006986	25	24	13	108	74	39	72
Fam64a	ENSMUSG00000020808	76.37765644	-1.841284244	0.437888482	-4.204915906	2.61179E-05	0.004734332	42	25	25	121	122	56	143
Frmd5	ENSMUSG00000027238	436.7324839	-1.84676014	0.529540104	-3.487479277	0.000487597	0.04539098	159	226	143	237	357	930	1005
Nr0b1	ENSMUSG00000025056	325.7097679	-1.850384052	0.488516135	-3.787764456	0.000152009	0.018604729	146	139	107	170	682	360	676
1700112E06Rik	ENSMUSG00000063458	55.15713315	-1.867458644	0.565566	-3.301928763	0.000960225	0.069286649	15	17	33	53	158	43	67

Table S5. Genes differentially expressed in ABC Male vs ABC Female

								Read count						
Gene	ENSEMBL_Gene_ID	baseMean	log2FoldChange	lfcSE	stat	pvalue	padj	166836_No	166838_No	182098_Tu	184208_Tu	183159_B2	BS05_Norm	207386_No
Emb	ENSMUSG00000021728	155.8826013	-1.869491062	0.513453283	-3.641014919	0.000271565	0.029078694	82	45	59	74	215	285	332
Kif22	ENSMUSG00000030677	116.7549732	-1.875777641	0.453142352	-4.13948869	3.48081E-05	0.005956802	57	45	37	231	221	74	153
Rem2	ENSMUSG00000022176	14.01369658	-1.893969038	0.582350243	-3.252285135	0.001144811	0.077406846	8	5	4	17	27	17	21
Ncapg	ENSMUSG00000015880	50.83331488	-1.897807149	0.540205372	-3.513121577	0.000442875	0.042397587	31	16	13	119	56	36	85
S100a10	ENSMUSG00000041959	2051.755427	-1.914076643	0.607987351	-3.148217868	0.001642692	0.098960685	703	828	852	1137	7194	1337	2312
Birc5	ENSMUSG00000017716	97.31256043	-1.930686711	0.510567521	-3.781452272	0.000155916	0.01896293	35	35	42	125	267	60	118
Inpp5j	ENSMUSG00000034570	91.70348421	-1.940385187	0.442572132	-4.38433658	1.1634E-05	0.00254497	30	22	53	201	98	136	103
Mycbpap	ENSMUSG00000039110	29.52204944	-1.982556933	0.590825575	-3.355570605	0.000792014	0.061509935	16	4	13	36	72	25	40
Gm24187	ENSMUSG00000088609	453.0040442	-1.98324731	0.544782099	-3.640441408	0.000272171	0.029078694	146	167	192	204	1289	481	692
Alyref2	ENSMUSG00000060244	441.4298057	-1.992852566	0.365340782	-5.454777203	4.90343E-08	2.10717E-05	157	137	196	867	352	648	733
Ppp1r14c	ENSMUSG00000040653	33.95426671	-1.999100748	0.545923548	-3.661869427	0.000250381	0.027355237	14	7	16	25	40	63	72
Mxd3	ENSMUSG00000021485	45.57574093	-2.008695854	0.523632198	-3.836081624	0.000125013	0.015969121	23	11	16	106	72	31	59
Crybb1	ENSMUSG00000029343	66.87009272	-2.009177723	0.576168758	-3.4871341	0.000488226	0.04539098	23	17	33	53	193	46	104
Mir6236	ENSMUSG00000098973	1713.063451	-2.027893119	0.558517443	-3.630850108	0.000282489	0.029851238	639	570	654	783	5052	1484	2810
Gm24270	ENSMUSG00000076281	713.6372043	-2.034749919	0.553328043	-3.67729405	0.000235721	0.026502196	236	268	269	305	2048	721	1148
Hist1h2ap	ENSMUSG00000094777	110.003827	-2.067450657	0.655266665	-3.155128692	0.001604273	0.097557975	30	31	56	61	377	67	148
Scara5	ENSMUSG00000022032	105.9674828	-2.067792976	0.563047735	-3.672500302	0.000240189	0.026848395	64	36	12	102	255	132	140
Gm24245	ENSMUSG00000087943	448.185255	-2.069268751	0.571070256	-3.623492436	0.000290652	0.030218402	145	159	171	188	1346	438	690
Shisa9	ENSMUSG00000022494	140.3342313	-2.07847188	0.467704046	-4.443989519	8.83059E-06	0.002009011	65	32	50	352	107	188	188
Adh1	ENSMUSG00000074207	47241.2675	-2.078579631	0.598304764	-3.474115127	0.000512541	0.046974025	8318	6252	35293	39932	66282	74074	100538
RP23-291B1.2	ENSMUSG00000108434	36.73751222	-2.082498913	0.608410613	-3.422851064	0.00061968	0.0528984	12	6	21	79	76	25	39
Serpinb1b	ENSMUSG00000051029	254.6423568	-2.090009775	0.617520043	-3.384521357	0.000713025	0.057934756	104	43	120	606	80	395	434
D630024D03Rik	ENSMUSG00000085772	49.04756164	-2.097089417	0.650471761	-3.223951513	0.001264348	0.082323107	15	16	19	22	123	33	114
Fndc7	ENSMUSG00000045326	39.76457286	-2.099999461	0.415743677	-5.051187974	4.39071E-07	0.000143911	20	12	9	56	66	52	64
Gm25911	ENSMUSG00000088246	239.1612745	-2.111669712	0.577107488	-3.659057896	0.000253144	0.027501694	85	80	82	105	753	248	321
Cenpm	ENSMUSG00000068101	40.6497048	-2.13151597	0.480061531	-4.440089097	8.99216E-06	0.002021982	13	13	15	64	84	29	66
Naip5	ENSMUSG00000071203	117.3526326	-2.140966332	0.445596734	-4.804717287	1.5497E-06	0.000416224	48	38	33	74	226	205	197
Gm23935	ENSMUSG00000076258	2305.92874	-2.173982613	0.644946016	-3.370797801	0.000749508	0.059401619	653	727	920	915	8354	1697	2875
Dner	ENSMUSG00000036766	168.3174876	-2.217798379	0.426801021	-5.19632866	2.03263E-07	7.0191E-05	86	35	43	312	182	190	331
Fam19a4	ENSMUSG00000046500	164.3353684	-2.228942376	0.439185443	-5.075173623	3.87142E-07	0.000129079	41	73	45	337	336	184	136
Ush1g	ENSMUSG00000045288	11.8689791	-2.230086498	0.691576289	-3.224642794	0.001261299	0.082323107	3	5	4	14	15	30	13
Mmp19	ENSMUSG00000025355	226.1860891	-2.258726748	0.458016892	-4.931535905	8.15856E-07	0.000235478	86	76	52	153	385	288	543
Mmel1	ENSMUSG00000058183	21.70531804	-2.274113488	0.662951885	-3.43028437	0.000602949	0.052052802	7	7	7	14	67	25	26
Gulo	ENSMUSG00000034450	50.30384396	-2.278974873	0.552429213	-4.125369947	3.70139E-05	0.006224134	14	25	8	77	119	74	35
Amhr2	ENSMUSG00000023047	205.1019299	-2.301715859	0.371905746	-6.188976329	6.05562E-10	4.68414E-07	71	61	57	452	353	243	198
Esyt3	ENSMUSG00000037681	31.03648417	-2.35340642	0.743123308	-3.1669124	0.001540667	0.09482755	13	9	6	108	27	42	13
Fmo1	ENSMUSG00000040181	270.8462693	-2.385651942	0.582839211	-4.09315622	4.25541E-05	0.00685759	65	77	95	112	650	243	654
Tmem144	ENSMUSG00000027956	148.5656885	-2.389932092	0.434885623	-5.495541733	3.89513E-08	1.75172E-05	39	26	65	128	233	270	280
Ly6k	ENSMUSG00000044678	489.0540166	-2.44829952	0.594190934	-4.120391911	3.78228E-05	0.006251438	189	180	45	981	290	553	1186
Gabrg3	ENSMUSG00000055026	185.1099507	-2.456758825	0.294609873	-8.339024085	7.49062E-17	1.44854E-13	67	51	38	316	258	269	297
Vwc2	ENSMUSG00000050830	15.02999312	-2.485554985	0.649395463	-3.82749053	0.000129456	0.016151143	4	6	3	31	20	29	13
Fhdc1	ENSMUSG00000041842	44.16922598	-2.529814669	0.62072919	-4.075552927	4.59051E-05	0.007284574	13	8	15	38	25	110	101
Lingo3	ENSMUSG00000051067	70.61842061	-2.554456256	0.475576101	-5.371288112	7.81762E-08	3.08525E-05	19	15	21	53	135	91	160
Baalc	ENSMUSG00000022296	8.472964165	-2.554891508	0.79861601	-3.199148871	0.00137834	0.08767873	4	2	1	13	9	12	18
Cela1	ENSMUSG00000023031	633.9321016	-2.571735783	0.447421654	-5.74790192	9.03576E-09	4.72253E-06	166	136	195	479	1663	1101	697
2810459M11Rik	ENSMUSG00000026227	18.60120055	-2.587041103	0.591435529	-4.374172633	1.21894E-05	0.00254497	4	3	8	35	32	29	19
Cdo1	ENSMUSG00000033022	142.8315765	-2.591232582	0.778117758	-3.330129093	0.000868057	0.065139807	64	30	16	70	349	44	426
Rassf10	ENSMUSG00000098132	68.24363666	-2.6120476	0.520193498	-5.02129998	5.13229E-07	0.000160078	15	8	29	119	144	58	104
Cited4	ENSMUSG00000070803	46.50332177	-2.648380369	0.605216973	-4.375918865	1.20922E-05	0.00254497	11	14	10	28	143	52	68
Cxadr	ENSMUSG00000022865	120.0945972	-2.710511317	0.497652054	-5.446599276	5.1342E-08	2.15837E-05	44	33	9	247	191	174	142
Htr1b	ENSMUSG00000049511	154.601061	-2.723771806	0.503440841	-5.410311568	6.29152E-08	2.58863E-05	22	66	22	255	297	293	127
4930425O10Rik	ENSMUSG00000105838	15.0433991	-2.752441472	0.700401513	-3.929805149	8.50147E-05	0.011742963	3	1	7	32	15	25	22
Egfl6	ENSMUSG00000000402	2058.92027	-2.763894532	0.506075537	-5.461426858	4.72323E-08	2.07586E-05	500	673	260	5285	1825	4142	1728
Cdh12	ENSMUSG00000040452	61.23208544	-2.77561066	0.526783605	-5.268976927	1.37186E-07	4.91279E-05	12	15	16	128	55	52	152
Ggt6	ENSMUSG00000040471	17.04239719	-2.791458036	0.811953755	-3.437951999	0.000586132	0.05082786	5	4	3	7	59	17	24
Galnt13	ENSMUSG00000060988	9.884997685	-2.800790278	0.883224904	-3.171095226	0.001518654	0.093826586	3	1	3	5	15	26	17
D430036J16Rik	ENSMUSG00000097466	14.10555832	-2.813288318	0.724927936	-3.880783424	0.000104121	0.013790978	2	5	3	21	11	36	21
D430041D05Rik	ENSMUSG00000068373	155.8359259	-2.860962501	0.504460578	-5.671330184	1.41693E-08	7.02579E-06	34	32	36	76	310	283	320
Mfsd4	ENSMUSG00000059149	139.5038219	-2.895041787	0.514953987	-5.621942655	1.88822E-08	9.1286E-06	28	37	24	72	249	238	328
Cd1d1	ENSMUSG00000028076	580.9496784	-2.897799452	0.6037118	-4.799971525	1.58688E-06	0.000420372	112	113	147	240	1913	531	1012
Sstr2	ENSMUSG00000047904	22.61254183	-2.914115423	0.900577733	-3.235828864	0.001212901	0.079779168	7	6	2	43	9	83	8
Mme	ENSMUSG00000027820	111.7661109	-2.923466112	0.553890718	-5.278055792	1.30562E-07	4.76378E-05	17	15	38	76	218	291	127
4930486L24Rik	ENSMUSG00000050345	79.45703757	-2.926217556	0.391501956	-7.474336994	7.75941E-14	9.37821E-11	17	12	20	130	83	146	147
Cdh9	ENSMUSG00000025370	15.46659329	-2.948628556	0.741230012	-3.978021004	6.94912E-05	0.009961121	3	5	2	10	24	40	24
Dsp	ENSMUSG00000054889	330.654795	-2.984161892	0.480539955	-6.210018259	5.29784E-10	4.65681E-07	55	120	25	630	520	440	524
Epn3	ENSMUSG00000010080	95.20776037	-3.010785114	0.493756336	-6.097714386	1.07596E-09	7.43102E-07	24	25	8	165	176	80	189
Hrasls	ENSMUSG00000022525	22.52442587	-3.108111811	0.785790595	-3.955394517	7.64085E-05	0.01063012	2	3	8	11	68	25	41
Gm973	ENSMUSG00000047361	40.01581884	-3.120559515	0.83263813	-3.747797997	0.000178394	0.021035243	7	8	8	11	160	23	64
Fam84a	ENSMUSG00000020607	50.8749915	-3.137788083	0.589472626	-5.323042909	1.02046E-07	3.86933E-05	10	9	10	24	108	90	106
Dbx2	ENSMUSG00000045608	60.85826544	-3.170584977	0.78730173	-4.027153574	5.64562E-05	0.008529291	4	9	20	253	48	42	51
Unc5d	ENSMUSG00000063626	21.46048215	-3.183242073	0.655091593	-4.859232061	1.17842E-06	0.000320962	4	6	2	44	24	49	21
Gm26035	ENSMUSG00000064999	13.83528122	-3.309106515	0.987981189	-3.349361861	0.000809979	0.062156272	4	2	1	8	57	7	17
Retn	ENSMUSG00000012705	75.64858107	-3.335637993	0.997745254	-3.343176007	0.000828253	0.063058134	29	5	3	36	305	12	140
Bmp3	ENSMUSG00000029335	10.68718976	-3.433998985	0.89541627	-3.835086652	0.00012552	0.015969121	2	2	1	7	25	14	23
Sgk2	ENSMUSG00000017868	6.718800306	-3.527832479	1.071726947	-3.291726955	0.000995742	0.070276153	2	1	0	7	8	14	15
Serpinb1a	ENSMUSG00000044734	296.9286997	-3.606091796	0.653871711	-5.514983651	3.48812E-08	1.6452E-05	38	24	58	109	775	271	802
Eps8l1	ENSMUSG00000006154	99.81279086	-3.609712235	1.01889335	-3.542777305	0.000395937	0.039064428	9	16	16	10	429	205	14
Nxph1	ENSMUSG00000046178	11.91981497	-3.624089067	0.909661085	-3.983999233	6.77651E-05	0.009894621	1	1	3	16	17	36	10
Nell2	ENSMUSG00000022454	33.59405548	-3.631792508	0.614920855	-5.906113737	3.50272E-09	2.0526E-06	3	8	3	43	32	65	82
Frzb	ENSMUSG00000027004	2394.656272	-3.647256939	0.486309683	-7.499864943	6.38836E-14	8.82415E-11	423	268	255	1758	2825	4003	7231
Sbsn	ENSMUSG00000046056	556.0781164	-3.716074653	0.756348567	-4.913177358	8.96121E-07	0.000251148	63	52	95	120	2270	424	869
Ms4a10	ENSMUSG00000024731	40.48393498	-3.901047176	1.04714372	-3.725417152	0.000194992	0.02257943	5	2	7	3	178	20	69
Elfn1	ENSMUSG00000048888	58.28687739	-3.915241641	0.627480592	-6.239621894	4.3863E-10	4.03915E-07	8	2	10	124	130	38	97
Mgat4c	ENSMUSG00000019888	391.9290944	-3.961091899	0.671626453	-5.897760404	3.68468E-09	2.09572E-06	52	37	37	333	239	335	1710
Lhx3	ENSMUSG00000026934	21.1282811	-3.96239845	0.846496986	-4.680936275	2.85568E-06	0.00072662	3	3	1	19	78	23	22
Atp1a4	ENSMUSG00000007107	47.57870302	-4.043935558	0.959480245	-4.21471477	2.50094E-05	0.004650307	10	2	3	15	183	15	106
Dnmt3l	ENSMUSG00000000730	52.29392544	-4.087592771	0.825776346	-4.949999825	7.42135E-07	0.000217446	5	3	8	11	178	62	100
RP23-430B14.2	ENSMUSG00000108801	6.77194221	-4.146847862	1.270413603	-3.264171489	0.001097847	0.074754099	1	1	0	4	11	13	18
Hydin	ENSMUSG00000059854	10.75239015	-4.249788431	1.160072722	-3.663381053	0.000248908	0.027355237	0	3	0	31	5	23	13
Cryba4	ENSMUSG00000066975	1536.181555	-4.255609669	1.253152049	-3.39592444	0.000683972	0.056683703	175	165	66	256	8685	933	473
Cst8	ENSMUSG00000027442	61.79887059	-4.342032325	1.02992163	-4.215886141	2.48799E-05	0.004650307	5	6	5	9	286	11	111
Serpinb1c	ENSMUSG00000079049	7.760513366	-4.350336803	1.187995526	-3.661913458	0.000250338	0.027355237	1	0	1	14	11	10	18
1700028P14Rik	ENSMUSG00000033053	8.383009338	-4.444249902	1.255353234	-3.54023854	0.000399766	0.039241958	0	0	2	10	27	8	12
Fam169a	ENSMUSG00000041817	41.5364635	-4.456711	0.764907762	-5.826468524	5.66125E-09	3.12792E-06	4	3	3	22	39	115	105
Vsig8	ENSMUSG00000049598	12.63372062	-4.458341732	1.290762909	-3.454036137	0.000552263	0.049442912	2	0	1	2	55	10	19
D730001G18Rik	ENSMUSG00000101026	4.76981677	-4.563448848	1.433532713	-3.183358709	0.001455771	0.091401649	0	1	0	6	4	8	14
Col5a3	ENSMUSG00000004098	596.4902493	-4.59997774	0.553841691	-8.305582285	9.93311E-17	1.60072E-13	43	31	50	346	1400	594	1711
Entpd3	ENSMUSG00000041608	15.26594916	-4.759088498	1.174991191	-4.050318449	5.1148E-05	0.007912797	0	2	1	6	12	30	56
Vnn3	ENSMUSG00000020010	93.77038807	-4.788960059	0.795266818	-6.021828087	1.72458E-09	1.04219E-06	5	8	5	25	352	87	176
Mapk13	ENSMUSG00000004864	707.4258601	-4.808167229	0.731302538	-6.574799043	4.87189E-11	4.71063E-08	25	42	62	179	2716	649	1279
C3	ENSMUSG00000024164	4137.042901	-5.231474988	0.844717955	-6.19316182	5.89691E-10	4.68414E-07	284	140	143	1546	19487	885	6474
Gm15883	ENSMUSG00000086868	4.05915213	-5.312743487	1.542097133	-3.445141925	0.00057076	0.049942759	0	0	0	2	12	8	6

Table S5. Genes differentially expressed in ABC Male vs ABC Female

								Read count						
Gene	ENSEMBL_Gene_ID	baseMean	log2FoldChange	lfcSE	stat	pvalue	padj	166836_No	166838_No	182098_Tu	184208_Tu	183159_B2	BS05_Norm	207386_No
Apoc1	ENSMUSG00000040564	486.444706	-5.378689002	1.650847625	-3.258137772	0.001121459	0.076093975	6	48	7	543	202	3	2597
Cidea	ENSMUSG00000024526	60.85767602	-5.767356799	1.566332448	-3.682077075	0.000231341	0.026161876	3	1	2	3	291	7	119
Folr1	ENSMUSG00000001827	21.63718195	-5.856602024	1.498465116	-3.908400645	9.29091E-05	0.012564175	2	0	0	6	107	3	33
Orm2	ENSMUSG00000061540	6.321953858	-5.945781945	1.675771518	-3.548086288	0.000388041	0.038481735	0	0	0	6	29	1	8
Orm1	ENSMUSG00000039196	23.08098032	-5.947564422	1.582827191	-3.757557652	0.00017158	0.02048155	0	0	2	8	118	1	33
Adig	ENSMUSG00000044405	36.45441964	-6.032101992	1.810896968	-3.331002315	0.000865339	0.065139807	3	0	0	9	172	5	66
Oxtr	ENSMUSG00000049112	315.5640977	-6.214215352	0.622140777	-9.988439241	1.71257E-23	4.73109E-20	4	13	5	175	698	593	721
Txndc2	ENSMUSG00000050612	29.00536007	-6.28462254	1.248323449	-5.03445044	4.79222E-07	0.000154453	0	1	1	12	108	38	42
Mgst2	ENSMUSG00000074604	564.4611447	-6.825243121	0.707233244	-9.650625415	4.88568E-22	1.18099E-18	8	11	7	327	2438	669	492
Ucp1	ENSMUSG00000031710	46.68430773	-6.982986604	2.210874044	-3.158473284	0.001585978	0.096749689	2	0	0	2	180	2	141
1700125H20Rik	ENSMUSG00000018479	25.39644423	-6.995469086	1.472460062	-4.750871867	2.02541E-06	0.00052929	0	0	1	5	104	32	36
Ly6h	ENSMUSG00000022577	13.21714983	-7.015838199	1.538218648	-4.561014918	5.0907E-06	0.001246125	0	0	0	6	66	10	12
Cyp2f2	ENSMUSG00000052974	1249.062152	-7.642574958	0.710533984	-10.75610053	5.54679E-27	2.14528E-23	17	10	6	530	2029	1242	4909
Xist	ENSMUSG00000086503	979.6871197	-9.79681836	0.793335221	-12.34890133	4.93812E-35	4.77467E-31	2	1	3	3453	1356	1215	828
Akr1d1	ENSMUSG00000038641	2196.634761	-14.40659791	1.361935368	-10.57803347	3.76783E-26	1.21437E-22	0	0	0	749	7326	2999	4302

Table S6. GSEA report for male ABC_Hoxb9 tumours compared to male ABC tumours

NAME	SIZE	ES	NES	NOM p-val	FDR q-val	FWER p-val	RANK AT MAX
HALLMARK_G2M_CHECKPOINT	189	0.6468555	1.697571	0	0.04399998	0	2163
HALLMARK_MYC_TARGETS_V2	57	0.64348674	1.5850172	0	0.06618747	0.115	3399
HALLMARK_E2F_TARGETS	191	0.62671053	1.5686973	0	0.06945833	0.163	2932
HALLMARK_SPERMATOGENESIS	95	0.4395258	1.4903258	0.17158672	0.20406246	0.543	1001
HALLMARK_MYC_TARGETS_V1	191	0.51113737	1.4716262	0	0.18284994	0.543	4372
HALLMARK_TNFA_SIGNALING_VIA_NFKB	183	0.3626254	1.3311056	0	0.34334636	0.808	1244
HALLMARK_TGF_BETA_SIGNALING	52	0.30804762	1.1985636	0.102345414	0.6009473	0.947	2640
HALLMARK_REACTIVE_OXIGEN_SPECIES_PATHWAY	45	0.4346205	1.18057	0.31212723	0.59848905	0.947	4305
HALLMARK_KRAS_SIGNALING_DN	123	0.31556696	1.1704465	0.17073171	0.5797694	1	1550
HALLMARK_MITOTIC_SPINDLE	198	0.45075125	1.1701288	0.10331384	0.5261925	1	1201
HALLMARK_UV_RESPONSE_UP	140	0.2766306	1.1253206	0.11044177	0.5694552	1	3332
HALLMARK_OXIDATIVE_PHOSPHORYLATION	189	0.46338195	1.1154771	0.30604288	0.53661823	1	4469
HALLMARK_FATTY_ACID_METABOLISM	142	0.30793777	1.1061171	0.41351888	0.50957435	1	2996
HALLMARK_ESTROGEN_RESPONSE_LATE	172	0.28641844	1.0496027	0.3592233	0.5719434	1	1172
HALLMARK_GLYCOLYSIS	180	0.19225512	0.95989305	0.52642274	0.70349604	1	2646
HALLMARK_PI3K_AKT_MTOR_SIGNALING	99	0.21583931	0.9114872	0.62355214	0.7756091	1	1283
HALLMARK_DNA_REPAIR	136	0.2382726	0.8295817	0.6276803	0.8557615	1	4594
HALLMARK_P53_PATHWAY	187	0.18670382	0.827854	0.6312849	0.81244224	1	3007
HALLMARK_ADIPOGENESIS	187	0.17481901	0.7299593	0.9116466	0.8920309	1	4100
HALLMARK_ALLOGRAFT_REJECTION	169	0.16238579	0.46229643	0.915547	0.987912	1	1807

Table S7. Pearson r values of HOX gene expression correlation with the expression of other HOX genes and proliferation markers in TCGA ACC samples

	HOXA1	HOXA2	HOXA3	HOXA4	HOXA5	HOXA6	HOXA7	HOXA9	HOXA10	HOXA11	HOXA13	HOXB1	HOXB2	HOXB3	HOXB4	HOXB5	HOXB6	HOXB7	HOXB8	HOXB9	HOXB13	HOXC4	HOXC5	HOXC6	HOXC8	HOXC9	HOXC10	HOXC11	HOXC12	HOXC13	HOXD1	HOXD3	HOXD4	HOXD8	HOXD9	HOXD10	HOXD11	HOXD12	HOXD13
HOXA1	1	0.42585	0.48905	0.54974	0.38186	0.24093	0.3196	0.06487	0.40922	0.25273	0.30168	0.04596	0.02047	0.06379	0.06725	0.06827	0.08185	0.16274	0.1716	0.19879	0.1631	0.23555	0.1138	0.14199	0.20105	0.26927	0.39635	0.25792	0.17351	0.22436	0.25026	0.0956	0.01329	0.02512	0.15966	0.2157	0.11424	0.11239	0.10398
HOXA2	0.42585	1	0.52545	0.31418	0.32777	0.00583	0.08669	0.05939	0.1359	0.14222	0.0376	-0.01168	0.04776	0.16798	0.09885	0.15931	0.2154	0.14165	0.20148	0.14618	-0.06404	0.13385	0.26637	0.29954	0.35714	0.17633	0.1095	0.1568	0.03744	0.05645	0.09858	0.23205	0.2312	0.07387	0.09607	0.01561	0.14528	0.11306	-0.0228
HOXA3	0.48905	0.52545	1	0.61143	0.55696	0.29079	0.47473	0.21521	0.30781	0.28599	0.20192	0.06939	0.11449	0.15649	-0.03118	0.24348	0.29101	0.34471	0.21693	0.35418	0.14018	0.28678	0.35863	0.32274	0.38876	0.32987	0.32633	0.2431	0.18347	0.14542	0.16701	0.24595	0.20836	0.19778	0.15263	0.07191	0.12141	0.05243	0.15167
HOXA4	0.54974	0.31418	0.61143	1	0.60896	0.27741	0.51887	0.18898	0.46408	0.27745	0.40732	0.09407	0.09845	0.26773	0.23638	0.25141	0.30161	0.40856	0.16543	0.29047	0.23836	0.35333	0.32679	0.26486	0.28921	0.33926	0.38691	0.26615	0.18437	0.34821	-0.02216	0.13028	0.14419	0.04838	0.15465	0.124	0.08885	0.00243	0.11441
HOXA5	0.38186	0.32777	0.55696	0.60906	1	0.58525	0.67468	0.07199	0.2457	0.0811	0.22613	0.05775	0.03955	0.11793	-0.03306	0.19558	0.12397	0.19152	0.0769	0.17999	0.03109	0.16869	0.13849	0.21224	0.19052	0.03712	0.08034	-0.11694	-0.00904	0.07515	-0.15035	0.13505	0.21	0.2402	0.17023	-0.00674	-0.07928	-0.14225	-0.11019
HOXA6	0.24093	0.00583	0.29079	0.27741	0.58525	1	0.60903	0.11931	0.28219	0.14576	0.4075	0.08152	-0.13657	-0.11777	-0.09501	-0.07552	-0.06129	-0.00633	-0.01296	0.02621	0.08414	0.25507	0.06227	0.10071	0.04549	-0.07121	0.08462	-0.18076	0.03989	0.02969	0.0017	0.22616	0.11117	0.16986	0.15522	0.00861	0.02574	-0.0903	-0.08524
HOXA7	0.3196	0.08669	0.47473	0.51887	0.67468	0.60903	1	0.34261	0.47654	0.40181	0.40624	0.02042	-0.03167	0.05999	-0.00876	0.20606	0.21421	0.34807	0.18305	0.28244	0.19512	0.29748	0.2389	0.28491	0.21	0.23754	0.27235	0.04683	0.13278	0.21782	-0.03008	0.16013	0.12399	0.27838	0.38383	0.29394	0.1651	-0.08918	0.10411
HOXA9	0.06487	0.00933	0.21521	0.18898	0.07199	0.11931	0.34261	1	0.55664	0.54019	0.49339	-0.00551	0.01767	0.14148	0.14339	0.18666	0.32138	0.31185	0.19296	0.28619	0.10776	0.14335	0.25308	0.25792	0.14809	0.24399	0.1272	0.24277	0.13939	0.30182	0.04356	0.18595	0.2281	0.22931	0.37376	0.34349	0.34218	-0.04561	0.08825
HOXA10	0.40922	0.1359	0.30781	0.46408	0.2457	0.28219	0.47654	0.55664	1	0.69704	0.77484	0.13025	0.11514	0.18678	0.13756	0.22291	0.37123	0.43458	0.38195	0.44082	0.28281	0.29685	0.32177	0.29081	0.24713	0.34792	0.38737	0.38945	0.27083	0.48296	0.04961	0.19958	0.15126	0.16562	0.36387	0.31914	0.30643	0.08721	0.2771
HOXA11	0.25273	0.14222	0.28599	0.27745	0.0811	0.14576	0.40181	0.54019	0.69704	1	0.64271	0.14555	0.04376	0.2079	0.15096	0.3958	0.51361	0.42594	0.43248	0.43208	0.30691	0.14409	0.25719	0.32356	0.2558	0.39733	0.5295	0.51502	0.38076	0.45556	-0.00801	0.37226	0.3517	0.25057	0.41268	0.46572	0.38938	0.14766	0.41695
HOXA13	0.30168	0.0376	0.20192	0.40732	0.22613	0.4075	0.40624	0.49339	0.77484	0.64271	1	0.05519	0.04519	0.1409	0.18078	0.12618	0.28797	0.33283	0.32402	0.3175	0.29464	0.32916	0.21326	0.21396	0.14773	0.22195	0.35346	0.27291	0.25283	0.44117	-0.009	0.2709	0.18927	0.27176	0.36876	0.28143	0.26618	0.05079	0.27771
HOXB1	0.04596	-0.01168	0.06939	0.09407	0.05775	0.08152	0.02042	-0.00551	0.13025	0.14555	0.05519	1	0.17892	0.09665	0.08928	0.093	0.11845	0.08854	0.15839	0.10435	0.0556	0.09052	0.15872	0.11982	0.15808	0.16946	0.13539	0.00772	-0.04519	0.13702	-0.0987	0.0015	0.00512	-0.02616	0.01108	0.03376	-0.096	-0.06268	-0.07021
HOXB2	0.02047	0.04776	0.11449	0.09945	0.03955	-0.11657	-0.03167	0.01767	0.11514	0.04376	0.04519	0.17892	1	0.67828	0.45612	0.27483	0.32286	0.3564	0.34185	0.33608	-0.07392	0.03767	0.10467	0.09047	0.17525	0.08836	-0.07821	0.04262	-0.0958	0.03735	0.15862	-0.08275	0.02849	0.11195	0.14338	0.11279	0.11158	0.0244	0.09401
HOXB3	0.06379	0.10798	0.15649	0.26773	0.11793	-0.11777	0.05999	0.14148	0.18678	0.2079	0.1409	0.09665	0.67828	1	0.63011	0.62639	0.66912	0.66364	0.39165	0.37347	0.16757	0.20063	0.27717	0.32184	0.30551	0.37616	0.21743	0.30489	0.09976	0.32015	0.07458	0.14402	0.25893	0.17467	0.27127	0.1981	0.13362	0.00628	0.13071
HOXB4	0.06725	0.09685	-0.03118	0.23638	-0.03306	-0.09501	-0.00676	0.14339	0.13756	0.19296	0.18078	0.06928	0.45612	0.63011	1	0.27982	0.44912	0.45926	0.36611	0.10759	0.04633	0.12366	0.18047	0.17237	0.18068	0.15142	0.05355	0.13391	0.0032	0.21064	0.14269	0.0193	0.05085	0.0449	0.23974	0.26396	0.15329	-0.08329	-0.02007
HOXB5	0.06827	0.15931	0.24348	0.25141	0.19558	-0.07552	0.20606	0.18666	0.22291	0.3958	0.12618	0.093	0.27483	0.62639	0.27982	1	0.81144	0.69141	0.58675	0.57456	0.29404	0.16007	0.27523	0.33165	0.29232	0.45249	0.37585	0.48163	0.30876	0.50949	0.06437	0.0766	0.23923	0.36844	0.34188	0.20169	0.1668	0.08875	0.29045
HOXB6	0.08185	0.2154	0.29101	0.30161	0.12397	-0.06129	0.21421	0.32138	0.37123	0.51161	0.28797	0.11845	0.32286	0.66912	0.44912	0.81144	1	0.79175	0.63634	0.59143	0.44766	0.18302	0.33408	0.38959	0.26199	0.50615	0.54813	0.54288	0.34248	0.5685	0.11594	0.23869	0.3185	0.36504	0.4445	0.35052	0.29607	0.11335	0.3691
HOXB7	0.16274	0.14165	0.34471	0.40856	0.19152	-0.00633	0.14887	0.11185	0.45458	0.42594	0.33283	0.08854	0.3564	0.66364	0.45926	0.69141	0.79175	1	0.59986	0.63467	0.47083	0.31125	0.33197	0.35208	0.23441	0.48859	0.50176	0.43426	0.2316	0.58119	0.08846	0.21483	0.29808	0.32732	0.54275	0.45175	0.29726	0.07609	0.31221
HOXB8	0.1716	0.20248	0.21693	0.16543	0.0769	-0.01296	0.18305	0.19296	0.38195	0.43248	0.32402	0.15839	0.34185	0.39365	0.36611	0.58675	0.63634	0.59986	1	0.63778	0.19421	0.03683	0.12032	0.16105	0.22147	0.24791	0.28368	0.32372	0.27496	0.33255	0.2484	-0.02672	0.0904	0.37061	0.44097	0.2994	0.34927	0.09743	0.36844
HOXB9	0.19879	0.14618	0.35418	0.29047	0.17999	0.02621	0.28244	0.28619	0.44082	0.43208	0.3175	0.10435	0.33608	0.37347	0.10759	0.57456	0.59143	0.63467	0.63778	1	0.51551	0.30289	0.25831	0.29577	0.26524	-0.05282	0.40486	0.56358	0.52556	0.40701	0.62935	-0.05225	0.28753	0.21325	0.1712	0.24312	0.34061	0.12606	0.20678
HOXB13	0.1631	-0.06404	0.14018	0.23836	0.03109	0.08414	0.19512	0.10795	0.28281	0.30691	0.29464	0.0556	-0.07392	0.16757	0.04633	0.29404	0.44766	0.47083	0.19421	0.51551	1	0.41415	0.20577	0.26524	-0.05282	0.40486	0.56358	0.52556	0.40701	0.62935	-0.05225	0.28753	0.21325	0.1712	0.24312	0.34061	0.12606	0.20678	0.52369
HOXC4	0.23555	0.13385	0.28678	0.35333	0.16869	0.25507	0.29748	0.14335	0.29685	0.14409	0.32916	0.09052	0.03767	0.20063	0.12266	0.16007	0.18302	0.31125	0.03683	0.30289	0.41415	1	0.63919	0.60868	0.40904	0.64478	0.47169	0.30937	0.16895	0.43143	0.16738	0.27479	0.17889	0.13449	0.11563	0.13147	0.14838	0.03838	0.25961
HOXC5	0.1138	0.26637	0.35863	0.32679	0.13849	0.06227	0.2389	0.25308	0.32177	0.25719	0.21326	0.15872	0.10467	0.27717	0.18047	0.27523	0.33408	0.33197	0.12032	0.25831	0.20577	0.63919	1	0.85104	0.75831	0.72881	0.53188	0.36767	0.22317	0.41917	0.13424	0.94251	0.29964	0.15275	0.10463	0.10264	0.1627	-0.02099	0.17393
HOXC6	0.14199	0.29954	0.32274	0.26486	0.21224	0.10071	0.28491	0.25792	0.29081	0.23156	0.21396	0.11982	0.09047	0.32384	0.17237	0.33165	0.38959	0.35206	0.16105	0.24173	0.26524	0.60868	0.85104	1	0.74485	0.7398	0.56637	0.37446	0.1702	0.39001	0.14561	0.48252	0.44609	0.18966	0.19061	0.16055	0.13741	-0.00458	0.15523
HOXC8	0.20115	0.35714	0.38876	0.28921	0.19052	0.04549	0.21	0.14809	0.24713	0.2558	0.14773	0.15808	0.17525	0.30551	0.18068	0.29232	0.26199	0.23441	0.22147	0.22229	-0.05282	0.40904	0.75831	0.74485	1	0.61652	0.3814	0.2357	0.14526	0.15872	0.15418	0.30198	0.33631	0.10591	0.10998	0.09443	0.13704	-0.02623	0.08091
HOXC9	0.26927	0.17633	0.32987	0.33926	0.03712	-0.07121	0.23754	0.24399	0.34792	0.39733	0.22195	0.16946	0.08926	0.37616	0.15142	0.45249	0.50615	0.48859	0.24791	0.37749	0.40486	0.64478	0.72881	0.7398	0.61652	1	0.76745	0.57721	0.29111	0.56883	0.12924	0.38688	0.33644	0.1696	0.31175	0.31811	0.24308	0.00052	0.36476
HOXC10	0.29635	0.1095	0.32633	0.38691	0.08034	0.00402	0.27235	0.3272	0.38737	0.5295	0.35346	0.13539	-0.07821	0.21743	0.05355	0.37585	0.54813	0.50176	0.28368	0.47483	0.56358	0.47169	0.53188	0.56637	0.3814	0.76745	1	0.67573	0.44955	0.69699	0.0245	0.38011	0.30344	0.12748	0.25542	0.40861	0.31961	0.11434	0.45981
HOXC11	0.25792	0.1568	0.2431	0.26615	-0.11694	-0.18076	0.04683	0.24277	0.38945	0.51502	0.27291	0.00772	0.04262	0.30489	0.11391	0.48163	0.54288	0.43426	0.32172	0.47236	0.52556	0.40904	0.75831	0.74485	0.61652	0.57721	0.67573	1	0.71188	0.77485	0.11679	0.23618	0.17825	0.09072	0.17189	0.23674	0.27116	0.18107	0.65593
HOXC12	0.17351	0.03744	0.18347	0.18437	-0.00904	0.03989	0.13278	0.13939	0.27083	0.38076	0.25283	-0.04519	-0.0958	0.09976	0.0032	0.30876	0.34248	0.2316	0.27496	0.27005	0.40701	0.16895	0.23117	0.17702	0.14526	0.29111	0.44955	0.71188	1	0.59676	0.19637	0.07715	0.06397	0.18182	0.12122	0.14211	0.18535	-0.00386	0.65073
HOXC13	0.22406	0.05645	0.14542	0.34821	0.07515	0.02969	0.21782	0.30182	0.48296	0.45556	0.44117	0.13702	0.03735	0.32015	0.21064	0.50049	0.5685	0.58119	0.32515	0.45555	0.62935	0.45143	0.41917	0.39001	0.15872	0.56883	0.69699	0.77485	0.59676	1	0.08314	0.17825	0.14775	0.19421	0.30495	0.3	0.2265	0.03488	0.45105
HOXD1	0.25026	0.09858	0.16701	-0.02216	-0.15035	0.0017	-0.03008	0.04356	0.04961	-0.00801	-0.009	-0.0987	0.15862	0.07458	0.14269	0.06437	0.11594	0.06846	0.2484	0.11528	-0.05225	0.16738	0.13424	0.14561	0.15418	0.12924	0.0245	0.11679	0.19637	0.00814	1	0.0697	-0.05521	0.26388	0.12133	0.08901	0.14615	0.23741	0.23162
HOXD3	0.0956	0.23201	0.24595	0.13028	0.13505	0.22616	0.16013	0.18595	0.19958	0.37226	0.2709	0.0015	-0.08275	0.14402	0.0193	0.0766	0.23869	0.21483	-0.02672	0.07759	0.28753	0.27479	0.94251	0.48252	0.33188	0.38688	0.38011	0.23618	0.07715	0.17825	0.0697	1	0.81831	0.39938	0.35244	0.19225	0.16739	0.12948	0.14925
HOXD4	0.01329	0.2332	0.20836	0.14419	0.21	0.11117	0.12399	0.2281	0.15126	0.3517	0.18927	0.00512	0.02849	0.25893	0.05085	0.23923	0.3185	0.29808	0.0904	0.187	0.21325	0.17889	0.29964	0.44609	0.33631	0.33644	0.30344	0.17825	0.06397	0.14775	-0.05521	0.81831	1	0.53852	0.44743	0.08627	0.06797	0.06407	0.12619
HOXD8	0.02512	0.07387	0.19778	0.04838	0.2402	0.16986	0.27838	0.22931	0.16562	0.25057	0.27376	-0.02616	0.11195	0.17467	0.0449	0.36844	0.36504	0.32732	0.37061	0.44766	0.62935	0.43143	0.41917	0.39001	0.15872	0.56883	0.69699	0.77485	0.59676	0.19421	0.14775	0.19421	0.30495	0.53852	1	0.44951	0.2046	0.17141	0.15427
HOXD9	0.15966	0.09607	0.15263	0.15465	0.17023	0.15522	0.38383	0.37276	0.36387	0.43208	0.36876	0.01108	0.14238	0.27127	0.23974	0.34188	0.4445	0.54275	0.44097	0.37909	0.24312	0.11563	0.10463	0.19061	0.10998	0.13175	0.25542	0.17189	0.12122	0.30495	0.12133	0.35244	0.44743	0.64951	1	0.6533	0.35924	0.03619	0.242
HOXD10	0.2157	0.01561	0.07191	0.124	-0.06674	0.00861	0.29394	0.34349	0.31914	0.44572	0.26143	0.03376	0.11279	0.1981	0.26396	0.20169	0.35052	0.45175	0.2994	0.37434	0.34061	0.13147	0.10264	0.16055	0.09443	0.31811	0.40861	0.23674	0.14211	0.3	0.08901	0.19225	0.08627	0.2046	0.6533	1	0.58416	0.21583	0.29669
HOXD11	0.11424	0.14528	0.12141	0.08885	-0.07928	0.02574	0.1651	0.34218	0.30643	0.38938	0.26638	-0.096	0.11158	0.13362	0.15329	0.1668	0.29907	0.29726	0.34927	0.26593	0.12606	0.14838	0.1627	0.13741	0.13704	0.24308	0.31961	0.27116	0.18535	0.2265	0.14615	0.16739	0.06797	0.17141	0.35924	0.58416	1	0.25588	0.44056
HOXD12	0.11239	0.11306	0.05243	0.00243	-0.14225	-0.0603	-0.08918	-0.04561	0.08721	0.14766	0.05079	-0.06268	0.0244	0.00628	-0.08329	0.08875	0.11335	0.07609	0.09743	0.22886	0.20678	0.03838	-0.02099	-0.00458	-0.02623	0.00052	0.11434	0.18107	-0.00386	0.03488	0.23741	0.12948	0.06407	0.15427	0.03619	0.21583	0.25588	1	0.40892
HOXD13	0.10398	-0.0228	0.15167	0.11441	-0.11019	-0.08524	0.10411	0.08825	0.2771	0.41695	0.27771	-0.07021	0.09401	0.13071	-0.02007	0.29045	0.3691	0.31221	0.36844	0.45357	0.52369	0.25961	0.17393	0.15523	0.08591	0.36476	0.45981	0.65563	0.65073	0.45105	0.23162	0.14925	0.12619	0.29506	0.242	0.29669	0.44056	0.40892	1
MKI67	0.13951	0.01471	0.10336	-0.00339	-0.17526	-0.09376	0.08809	0.18849	0.23795	0.33743	0.13956	0.03758	-0.21186	-0.05729	-0.1388	0.16742	0.16956	0.14928	0.03541	0.17483	0.28013	0.29875	0.16826	0.14275	0.0975	0.3634	0.4476	0.47243	0.34	0.3462	0.04662	0.13032	0.06211	0.00771	-0.08795	0.14469	0.17588	0.20752	0.47344
ProliferanS	0.1428	0.04582	0.08842	0.04808	-0.18191	-0.13002	0.05578	0.20708	0.29624	0.38083	0.22569	0.0838	-0.1469	-0.00327	-0.01337	0.17702	0.24288	0.23006	0.19246	0.21818	0.33995	0.33461	0.19745	0.16742	0.06802	0.37748	0.48325	0.55665	0.39319	0.44078	0.06085	0.09946	0.02989	-0.00253	-0.03751	0.15313	0.23459	0.15921	0.54112
CCNE1	0.13236	-0.05553	0.05831	0.11945	-0.17832	-0.05401	0.10747	0.37105	0.287	0.36309	0.31846	0.10305	-0.1704	0.04454	0.09803	0.17101	0.2115	0.18282	0.05637	0.20322	0.4784	0.33563	0.22216	0.33106	0.08974	0.43201	0.46981	0.51607	0.30481	0.49312	0.03655	0.1945	0.078	-0.08425	0.06602	0.18024	0.07029	-0.05096	0.25406

Table S8. Pearson p values of HOX gene expression correlation with the expression of other HOX genes and proliferation markers in TCGA ACC samples

	HOXA1	HOXA2	HOXA3	HOXA4	HOXA5	HOXA6	HOXA7	HOXA9	HOXA10	HOXA11	HOXA13	HOXB1	HOXB2	HOXB3	HOXB4	HOXB5	HOXB6	HOXB7	HOXB8	HOXB9	HOXB13	HOXC4	HOXC5	HOXC6	HOXC8	HOXC9	HOXC10	HOXC11	HOXC12	HOXC13	HOXD1	HOXD3	HOXD4	HOXD8	HOXD9	HOXD10	HOXD11	HOXD12	HOXD13
HOXA1	NA	9.13E-05	4.80E-06	1.54E-07	0.000516	0.032443	0.00409	0.570069	0.000181	0.024634	0.006893	0.687529	0.857906	0.576505	0.555957	0.549967	0.473285	0.151872	0.130491	0.079339	0.150939	0.036641	0.318007	0.211927	0.075174	0.016408	0.008005	0.021739	0.126203	0.046836	0.026121	0.401984	0.907451	0.826053	0.159875	0.056241	0.316113	0.324049	0.361786
HOXA2	9.13E-05	NA	6.62E-07	0.004805	0.003191	0.959343	0.447436	0.410268	0.232403	0.211264	0.742166	0.91864	0.675947	0.138941	0.195825	0.160805	0.056591	0.213045	0.075324	0.198636	0.575014	0.239591	0.017651	0.007323	0.001294	0.120074	0.336747	0.167573	0.745244	0.621187	0.387401	0.059645	0.038615	0.517623	0.199669	0.891424	0.201439	0.321179	0.841892
HOXA3	4.80E-06	6.62E-07	NA	2.16E-09	9.79E-08	0.009327	9.86E-06	0.056814	0.005788	0.010619	0.074335	0.543419	0.315036	0.168428	0.785032	0.030598	0.009271	0.001865	0.054823	0.001363	0.217894	0.010394	0.001173	0.00372	0.000399	0.00299	0.003335	0.03087	0.105556	0.200997	0.14127	0.028899	0.065367	0.088596	0.179397	0.528852	0.286468	0.646311	0.182108
HOXA4	1.54E-07	0.004805	2.16E-09	NA	2.59E-09	0.013319	1.08E-06	0.095326	1.65E-05	0.013305	0.008195	0.409604	0.397812	0.017059	0.835966	0.025418	0.006909	0.000186	0.145108	0.009407	0.034395	0.001403	0.001388	0.018329	0.009737	0.002224	0.000428	0.017749	0.103836	0.001663	0.846295	0.252463	0.204876	0.671972	0.173556	0.276271	0.436165	0.983063	0.31537
HOXA5	0.000516	0.003191	9.79E-08	2.59E-09	NA	1.47E-08	9.27E-12	0.528396	0.029067	0.477361	0.045083	0.61317	0.729311	0.300635	0.77242	0.084109	0.276379	0.090865	0.500588	0.112443	0.785602	0.137251	0.22354	0.068405	0.692602	0.745373	0.481524	0.304726	0.936974	0.51039	0.185992	0.235367	0.063238	0.03299	0.113651	0.558962	0.487336	0.211091	0.333668
HOXA6	0.032443	0.959343	0.009327	0.013319	1.47E-08	NA	2.59E-09	0.294982	0.011749	0.13993	0.000193	0.475123	0.230083	0.301277	0.409404	0.583184	0.969776	0.851865	0.054823	0.001363	0.217894	0.010394	0.001173	0.00372	0.000399	0.00299	0.003335	0.03087	0.105556	0.200997	0.14127	0.028899	0.065367	0.088596	0.179397	0.528852	0.286468	0.646311	0.182108
HOXA7	0.00409	0.447436	9.86E-06	1.08E-06	9.27E-12	2.59E-09	NA	0.001996	9.02E-06	0.000242	0.000203	0.852827	0.781703	0.409076	0.952865	0.068471	0.058006	0.00167	0.106374	0.011672	0.084854	0.007758	0.033216	0.01093	0.06134	0.035038	0.015175	0.683956	0.24341	0.053805	0.792452	0.158628	0.276285	0.012986	0.00048	0.008556	0.145924	0.434455	0.361185
HOXA9	0.570069	0.410268	0.056814	0.095326	0.528396	0.294982	0.001996	NA	9.93E-08	2.77E-07	5.10E-05	0.961572	0.877195	0.213602	0.207429	0.099534	0.003877	0.005145	0.088418	0.010563	0.344536	0.207551	0.024427	0.021741	0.192766	0.030237	0.003247	0.031102	0.220514	0.006868	0.703063	0.100844	0.043191	0.042065	0.000717	0.00194	0.002024	0.689769	0.439271
HOXA10	0.000181	0.232403	0.005788	1.65E-05	0.029067	0.011749	9.02E-06	9.99E-08	NA	9.66E-13	1.00E-12	0.252932	0.312281	0.099309	0.226886	0.048315	0.000757	6.28E-05	0.000514	8.83E-05	0.011557	0.007893	0.003832	0.009321	0.028111	0.003679	0.00042	0.003389	0.015772	6.55E-06	0.664167	0.007829	0.18329	0.144662	0.000979	0.004148	0.006022	0.447723	0.013425
HOXA11	0.024634	0.211264	0.010619	0.013305	0.477361	0.13993	0.000242	2.77E-07	9.66E-13	NA	1.71E-10	0.200595	0.701738	0.065987	0.088423	0.000306	1.29E-06	9.09E-05	6.88E-05	7.00E-05	0.00594	0.205195	0.022126	0.003629	0.022883	0.003288	5.24E-07	1.20E-06	0.000537	2.46E-05	0.944111	0.000729	0.001481	0.02593	6.82E-05	1.52E-05	0.00039	0.194079	0.000132
HOXA13	0.006883	0.742166	0.074335	0.000195	0.045083	0.000193	0.000203	5.10E-05	1.00E-12	1.71E-10	NA	0.629049	0.6925	0.215515	0.110851	0.267833	0.010068	0.002726	0.003579	0.004356	0.008391	0.003056	0.059156	0.058304	0.193847	0.049311	0.001396	0.014958	0.024571	4.71E-05	0.937229	0.015746	0.094794	0.014637	0.000886	0.019953	0.017646	0.656665	0.013215
HOXB1	0.687529	0.91864	0.543419	0.409604	0.61317	0.475123	0.858257	0.961572	0.252592	0.200595	0.629049	NA	0.114645	0.396827	0.544066	0.414942	0.298498	0.43778	0.163268	0.360091	0.626461	0.427562	0.162389	0.292888	0.164098	0.135426	0.234172	0.946172	0.692482	0.228541	0.386812	0.989551	0.964308	0.818995	0.922808	0.767688	0.399989	0.583138	0.538641
HOXB2	0.857906	0.675947	0.315036	0.397812	0.729311	0.230083	0.781703	0.877195	0.312281	0.701738	0.6925	0.114645	NA	6.53E-12	2.40E-05	0.014237	0.003708	0.001265	0.002046	0.00246	0.517341	0.74169	0.358623	0.427829	0.122383	0.434603	0.493271	0.709186	0.401007	0.743826	0.162857	0.468458	0.803202	0.325956	0.210659	0.322346	0.32756	0.830388	0.575158
HOXB3	0.576505	0.138941	0.168428	0.017059	0.300635	0.301277	0.409976	0.213602	0.099309	0.065987	0.215515	0.396827	6.53E-12	NA	4.91E-10	6.64E-10	1.58E-11	2.64E-11	0.000332	0.000699	0.139913	0.070242	0.013402	0.003599	0.004203	0.051931	0.205395	0.021211	0.123663	0.015599	0.880104	0.240394	0.956179	0.259895
HOXB4	0.555957	0.195805	0.785032	0.031966	0.77242	0.404904	0.952865	0.207429	0.226686	0.088423	0.110851	0.544066	2.40E-05	4.91E-10	NA	0.012505	3.30E-05	2.17E-05	0.000906	0.345303	0.685166	0.281519	0.11147	0.128756	0.111063	0.182827	0.61926	0.239368	0.977637	0.063417	0.209671	0.865933	0.656259	0.694395	0.033337	0.018743	0.177409	0.465525	0.860608
HOXB5	0.549967	0.160805	0.030598	0.025418	0.084109	0.583114	0.068471	0.099534	0.048315	0.000306	0.267833	0.414942	0.014237	6.64E-10	0.012505	NA	1.00E-12	1.74E-12	1.33E-08	3.68E-08	0.008532	0.158793	0.012698	0.002828	0.008945	2.83E-05	0.000941	7.00E-06	0.005631	2.64E-06	0.573946	0.502347	0.033723	0.000835	0.002494	0.074672	0.141776	0.436711	0.009414
HOXB6	0.473285	0.056591	0.009271	0.008909	0.276379	0.591588	0.058006	0.003877	0.000757	1.29E-06	0.010068	0.298498	0.003708	1.58E-11	3.30E-05	1.00E-12	NA	1.00E-12	2.93E-10	9.51E-09	3.52E-05	0.106426	0.002621	0.000387	0.019682	1.95E-06	1.71E-07	2.38E-07	0.002005	4.62E-08	0.308896	0.034137	0.004228	0.00094	4.06E-05	0.00154	0.008066	0.319914	0.000816
HOXB7	0.151872	0.213045	0.001865	0.000186	0.090865	0.95584	0.00167	0.005145	6.28E-05	9.09E-05	0.002726	2.64E-11	2.17E-05	1.74E-12	1.00E-12	NA	5.16E-09	3.37E-10	1.19E-05	0.005238	0.002801	0.001463	0.037587	4.92E-06	2.47E-06	8.37E-05	0.040007	1.96E-08	0.548852	0.057367	0.007629	0.003235	2.37E-07	2.93E-05	0.007804	0.505113	0.005093
HOXB8	0.130491	0.075324	0.054823	0.145108	0.500588	0.909776	0.106374	0.088418	0.000514	6.88E-05	0.003579	0.163268	0.002046	0.000332	0.000906	1.33E-08	2.93E-10	5.16E-09	NA	2.60E-10	0.088339	0.747256	0.290868	0.156211	0.049816	0.027606	0.011294	0.003612	0.01419	0.00275	0.027292	0.815166	0.428174	0.000774	4.75E-05	0.007352	0.001605	0.393012	0.000835
HOXB9	0.079039	0.198636	0.001363	0.009407	0.112443	0.818641	0.031672	0.010563	4.83E-05	7.00E-05	0.004356	0.360091	0.00246	0.000699	0.345303	3.08E-08	9.51E-09	3.37E-10	2.60E-10	NA	1.16E-06	0.000662	0.021536	0.031853	0.048958	0.000605	9.81E-06	8.68E-05	0.016089	2.46E-05	0.313673	0.494469	0.098906	0.003303	0.000571	0.003677	0.017847	0.042482	2.69E-05
HOXB13	0.150393	0.575014	0.217894	0.034395	0.785602	0.460978	0.084854	0.344536	0.011557	0.00594	0.008391	0.626461	0.517341	0.139913	0.685166	0.008532	3.52E-05	1.19E-05	0.088339	1.16E-06	NA	0.000148	0.068869	0.018157	0.64385	0.000215	6.38E-08	6.58E-07	0.000197	5.22E-10	0.647462	0.003186	0.059163	0.13141	0.030851	0.002129	0.268299	0.007489	7.33E-07
HOXC4	0.036641	0.239591	0.010394	0.001403	0.137251	0.023288	0.007758	0.207551	0.007893	0.205195	0.003056	0.427562	0.74169	0.076242	0.281519	0.158793	0.106426	0.005238	0.747256	0.090662	0.000148	NA	2.30E-10	2.66E-09	0.000182	1.43E-10	1.14E-05	0.005531	0.136631	7.20E-05	0.140362	0.014251	0.114698	0.237338	0.310217	0.24813	0.191866	0.737042	0.020864
HOXC5	0.318007	0.017651	0.001173	0.001388	0.22354	0.585629	0.033216	0.024427	0.003832	0.022126	0.059156	0.162389	0.358623	0.013402	0.11147	0.012698	0.002621	0.002801	0.290868	0.021536	0.068869	2.30E-10	NA	1.00E-12	4.44E-16	2.66E-14	4.55E-07	0.000858	0.048047	0.000121	0.230205	0.002003	0.007303	0.178979	0.358784	0.368035	0.151954	0.854344	0.125286
HOXC6	0.211927	0.007323	0.00372	0.013329	0.692602	0.460405	0.377165	0.01093	0.021741	0.009321	0.003629	0.358304	0.292888	0.427829	0.003599	0.128756	0.002828	0.000387	0.001463	0.156211	0.031853	0.018157	2.66E-09	1.00E-12	NA	3.55E-15	6.66E-15	5.32E-08	0.000674	0.133718	0.000181	0.200404	6.69E-06	3.78E-05	0.094109	0.092418	0.157538	0.227211	0.968026	0.171936
HOXC8	0.075174	0.001294	0.000399	0.000737	0.692602	0.690555	0.06124	0.192766	0.028111	0.022883	0.193847	0.164098	0.122383	0.006183	0.111063	0.008945	0.010682	0.037587	0.049816	0.048958	0.64385	0.000182	4.44E-16	3.55E-15	NA	1.46E-09	0.000524	0.036514	0.201491	0.162387	0.174876	0.0228	0.002443	0.352893	0.194593	0.407793	0.228462	0.81448	0.563849
HOXC9	0.016408	0.120074	0.00299	0.002224	0.745373	0.532843	0.035038	0.030237	0.001679	0.000288	0.049311	0.135426	0.434603	0.006634	0.182827	2.83E-05	1.95E-06	4.92E-06	0.027606	0.000605	0.000215	1.43E-10	2.66E-14	6.66E-15	1.46E-09	NA	1.00E-12	2.57E-08	0.009246	4.52E-08	0.256288	0.000428	0.002433	0.135111	0.005161	0.004278	0.030884	0.996163	0.00095
HOXC10	0.008005	0.336747	0.003335	0.000428	0.481524	0.967781	0.015175	0.003247	0.00042	5.24E-07	0.001396	0.234172	0.493271	0.054243	0.61926	0.000941	1.71E-07	2.47E-06	0.011294	9.81E-06	6.38E-08	1.14E-05	4.55E-07	5.32E-08	0.000524	1.00E-12	NA	8.38E-12	3.24E-05	9.71E-13	0.830305	0.00055	0.006558	0.262885	0.023093	0.000185	0.00409	0.315663	2.02E-05
HOXC11	0.021739	0.167573	0.03087	0.017749	0.304726	0.1105	0.683956	0.011022	0.000389	1.20E-06	0.014958	0.946172	0.709186	0.006293	0.239368	7.00E-06	2.38E-07	6.37E-05	0.009612	8.60E-05	6.58E-07	0.000181	0.000674	0.036514	2.57E-08	8.38E-12	NA	1.91E-11	1.00E-12	0.305171	0.006124	0.116031	0.426541	0.129843	0.035672	0.015642	0.110265	5.48E-11
HOXC12	0.126203	0.743244	0.105556	0.103836	0.936974	0.72703	0.24341	0.220514	0.015772	0.000537	0.024571	0.692482	0.401007	0.381725	0.977637	0.005631	0.002005	0.040007	0.01419	0.016089	0.000197	0.136631	0.048047	0.133718	0.201491	0.009246	3.24E-05	1.91E-11	NA	6.47E-09	0.082831	0.499139	0.575422	0.108783	0.287253	0.211533	0.101968	0.973046	8.49E-11
HOXC13	0.046836	0.621187	0.200997	0.001663	0.51039	0.795073	0.053805	0.006868	6.55E-06	2.46E-05	4.71E-05	0.228541	0.743826	0.004023	0.062417	2.64E-06	4.62E-08	1.96E-08	0.00275	2.46E-05	5.22E-10	7.20E-05	0.000121	0.000181	0.162387	4.52E-08	9.71E-13	1.00E-12	6.47E-09	NA	0.94125	0.116021	0.193789	0.088337	0.006282	0.007229	0.044723	0.760267	3.02E-05
HOXD1	0.026121	0.387401	0.14127	0.846295	0.185992	0.988153	0.792452	0.703063	0.664167	0.944111	0.937229	0.386812	0.162857	0.513591	0.205971	0.573046	0.308896	0.548852	0.027292	0.313673	0.647462	0.140362	0.230205	0.200404	0.174876	0.256288	0.830305	0.305371	0.082831	0.94325	NA	0.541615	0.628394	0.018782	0.286817	0.43532	0.198714	0.035139	0.039983
HOXD3	0.401984	0.059645	0.028899	0.252463	0.235367	0.045256	0.158628	0.100844	0.077829	0.000729	0.015746	0.989551	0.468458	0.205395	0.865933	0.502347	0.034137	0.057367	0.815166	0.494469	0.010186	0.014251	0.002003	6.69E-06	0.0228	0.000428	0.00055	0.006124	0.499139	0.116021	0.541615	NA	1.00E-12	0.000266	0.001445	0.089618	0.140335	0.255424	0.189263
HOXD4	0.907451	0.038615	0.065367	0.204876	0.863238	0.32938	0.276285	0.043191	0.18329	0.001481	0.094794	0.964308	0.803202	0.021211	0.656259	0.033723	0.004228	0.007629	0.428174	0.098906	0.059163	0.114698	0.007303	3.78E-05	0.002443	0.002443	0.006558	0.116031	0.575422	0.193789	0.628394	1.00E-12	NA	3.07E-07	3.56E-05	0.44969	0.551715	0.57484	0.267777
HOXD8	0.826053	0.517623	0.088596	0.673972	0.03299	0.134502	0.012986	0.042065	0.144662	0.02593	0.014637	0.818995	0.325956	0.123663	0.694395	0.000835	0.00094	0.003235	0.000774	0.003303	0.13141	0.237338	0.178979	0.094109	0.952893	0.135111	0.262885	0.426541	0.108783	0.086337	0.018782	0.000266	3.07E-07	NA	9.45E-11	0.070496	0.130922	0.174636	0.008301
HOXD9	0.159875	0.399669	0.179307	0.173556	0.133651	0.171954	0.00048	0.000717	0.000979	6.82E-05	0.000886	0.922808	0.210659	0.015599	0.033337	0.002494	4.06E-05	2.37E-07	4.75E-05	0.000571	0.030851	0.310217	0.358784	0.092418	0.194593	0.005161	0.023093	0.129843	0.287253	0.006282	0.286817	0.001445	3.56E-05	9.45E-11	NA	6.76E-11	0.001148	0.751489	0.031654
HOXD10	0.056241	0.891424	0.528852	0.276271	0.558962	0.93994	0.008556	0.00194	0.004148	1.52E-05	0.019953	0.767688	0.322346	0.880104	0.018743	0.074672	0.00154	2.93E-05	0.007352	0.000677	0.002129	0.24813	0.368035	0.157538	0.407793	0.004278	0.000185	0.035672	0.211533	0.007229	0.43532	0.089618	0.44969	0.070496	6.76E-11	NA	1.59E-08	0.050289	0.007929
HOXD11	0.316113	0.201439	0.286468	0.436165	0.487336	0.821842	0.145924	0.002024	0.006022	0.00039	0.017646	0.399989	0.32756	0.240394	0.177409	0.141776	0.009414	0.002494	0.074672	0.042482	0.268299	0.191866	0.151954	0.227211	0.228462	0.030884	0.00409	0.015642	0.101968	0.044723	0.198714	0.140335	0.551715	0.130922	0.001148	1.59E-08	NA	0.02284	4.84E-05
HOXD12	0.324049	0.321179	0.646311	0.983063	0.211091	0.597557	0.434455	0.689769	0.444723	0.194079	0.656665	0.583138	0.830988	0.956179	0.465525	0.436711	0.319914	0.505113	0.393012	0.042482	0.067489	0.737042	0.854344	0.968026	0.81848	0.996363	0.315663	0.110265	0.973046	0.760267	0.035139	0.255424	0.57484	0.174636	0.751489	0.050289	0.02284	NA	0.000183
HOXD13	0.361786	0.841892	0.182108	0.31537	0.333668	0.455131	0.361185	0.439271	0.013425	0.000132	0.013215	0.538641	0.575158	0.259895	0.860608	0.009414	0.000816	0.005093	0.000835	2.69E-05	7.33E-07	0.020864	0.125286	0.171936	0.563849	0.00095	2.02E-05	5.48E-11	8.49E-11	3.02E-05	0.039983	0.189263	0.267777	0.008301	0.031654	0.007929	4.84E-05	0.000183	NA
MKI67	0.220096	0.897599	0.364706	0.97838	0.122366	0.411151	0.440131	0.096197	0.034718	0.002358	0.219958	0.742292	0.060883	0.616048	0.222488	0.14027	0.135207	0.189168	0.756686	0.123299	0.012405	0.007486	0.138259	0.209479	0.992637	0.000996	3.54E-05	1.10E-05	0.002171	0.001776	0.683267	0.252335	0.586591	0.946263	0.440835	0.203278	0.121029	0.06649	1.05E-05
Proliferation	0.209327	0.688407	0.438385	0.686731	0.168614	0.253403	0.625359	0.067078	0.008029	0.000535	0.045515	0.462801	0.196393	0.977198	0.90688	0.118595	0.031026	0.041382	0.244557	0.053402	0.002175	0.002577	0.081117	0.140279	0.546145	0.000605	6.45E-06	9.99E-08	0.000338	4.79E-05	0.594231	0.383107	0.793709	0.982371	0.742735	0.179864	0.937434	0.161057	2.62E-07
CCNE1	0.244912	0.626903	0.609757	0.28849	0.115883	0.636415	0.545833	0.000761	0.010333	0.001006	0.004233	0.17084	0.133252	0.696685	0.39009	0.113837	0.061324	0.106823	0.621736	0.07245	8.22E-06	0.002406	0.049087	0.002881	0.431582	0.000175	1.25E-05	1.13E-06	0.008398	4.79E-05	0.594231	0.383107	0.793709	0.982371	0.742735	0.179864	0.937434	0.161057	2.62E-07
